# Targeted Chemical Functionalization of the Amorphous Phase in Polyolefins via Controlled Solvent Swelling

**DOI:** 10.1021/acsmacrolett.6c00383

**Published:** 2026-08-31

**Authors:** Jin Qian, Tianli Ren, Peiran Wei, Wisdom Kemakolam, Zhe Qiang

**Affiliations:** † School of Polymer Science and Engineering, 5104 The University of Southern Mississippi, 118 College Drive, Hattiesburg, Mississippi 39406, United States; ‡ Mississippi Polymer Institute, The University of Southern Mississippi, Hattiesburg, Mississippi 39402, United States; § Soft Matter Facility, Department of Materials Science and Engineering, Texas A&M University, College Station, Texas 77845, United States

## Abstract

Postpolymerization modification of polyolefins enables direct introduction of functionality into high-volume, low-cost commodity materials, affording access to functional materials that are otherwise challenging to synthesize directly from monomers. However, traditional postpolymerization modification strategies result in randomly distributed functionalization along the polyolefin backbone, which can disrupt crystallinity and degrade the thermomechanical properties of the derived polymers. Crystallite-templated postpolymerization functionalization has recently emerged as a promising strategy to overcome these limitations, yet existing approaches often require specially prepared semicrystalline gels. Here, we report a swelling-assisted postpolymerization functionalization strategy that directly leverages the semicrystalline morphology of melt-processed polyolefins. In this process, the chemical reagents are selectively introduced to the amorphous phase of polymers for a targeted functionalization reaction at elevated temperatures. The resulting functionalized polyolefins largely retain the melting temperature and crystallinity of their unmodified counterparts across multiple grafting monomers, highlighting a practical and versatile platform for producing functional polyolefins with a preserved semicrystalline microstructure and bulk properties directly from commercial feedstocks.

## Graphical abstract

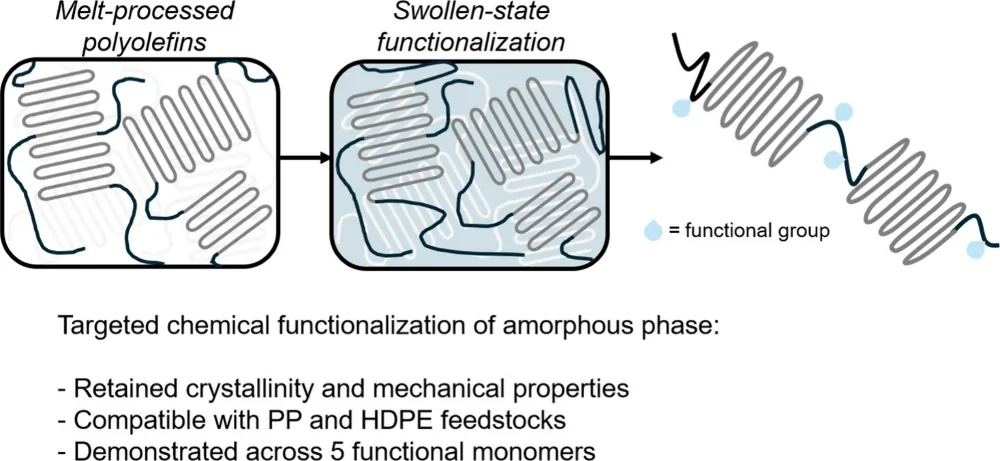

Polyolefins are the most widely used class of plastics owing to their low cost and excellent material properties. Their outstanding mechanical properties, chemical resistance, and thermal stability are largely attributed to their semicrystalline microstructures.^1,2^ Specifically, commodity polyethylene (PE) and polypropylene (PP) account for approximately 200 million metric tons of annual production, which are largely used in packaging but less suited for advanced applications such as interfacial adhesion and compatibilization, due to their lack of functional groups. While copolymerization to introduce functional groups on the polymer backbone is an effective strategy to address this limitation, persistent challenges include high cost, limited scalability, and system-to-system incompatibility across different catalyst platforms.^3–6^ Postpolymerization modification has emerged as a promising alternative, which enables the direct introduction of functionality into commercial polyolefins and affords access to a diverse range of materials that are otherwise impractical to synthesize directly. Various chemistries have been applied to post-polymerization modification of polyolefins.^7–13^ Among these, radical-induced C–H functionalization is one of the most classical, go-to approaches, as the process can be compatible with existing industrial infrastructure such as reactive extrusion.^14,15^ Traditionally, postpolymerization modification of polyolefins has relied primarily on mixing the polymer and reagents in either the solution or melt state, leading to functional groups incoporated randomly along the polymer backbone (Figure 1A).^16–18^ For semicrystalline polymers, the installed functional groups can act as defects during crystallization, reducing the crystallite size and degree of crystallinity, which consequently impact the thermal and mechanical properties of the material.^19–21^ Moreover, to ensure adequate mixing, processing temperatures typically exceed 150 °C, which promotes undesirable side reactions and rapid decomposition of radical initiators.^15^ Solid-state modification by reacting the bulk polymer directly in a functional monomer can be performed at lower temperatures, but it is limited by slow diffusion of reactants and is generally focused on polymer surface.^22^ Neidhart et al. reported gel-state C–H functionalization of polyolefins that enabled selective modification in the amorphous phase (Figure 1B).^23^ The resulting functionalized polyolefins with a “blocky” microstructure exhibited higher crystallinity and significantly improved mechanical properties compared to their randomly functionalized analogues. While this method leveraged steric protection from crystallites to achieve targeted amorphous-phase functionalization, it required the preparation of semicrystalline polyolefin gels. Furthermore, the semicrystalline morphology of the gel does not closely resemble that of standard melt-processed polyolefins. More recently, Pham et al. developed a crystallite-templated strategy for the solid-state hydrogenation of polycyclooctene (PCOE).^24^ The high chain mobility of PCOE allows catalytic hydrogenation in bulk without solvent assistance. Therefore, the amorphous domains of PCOE can be selectively hydrogenated to form polyethylene blocks while the tightly packed crystalline domains remain sterically inaccessible and are preserved. This study further demonstrated that PE block length is directly governed by crystallite morphology and can be tuned by controlling sample annealing conditions before hydrogenation, highlighting the importance of morphological control in crystallite-templated functionalization. However, direct, phase-selective functionalization of melt-processed commodity polyolefins remains a challenge.

**Figure 1:**
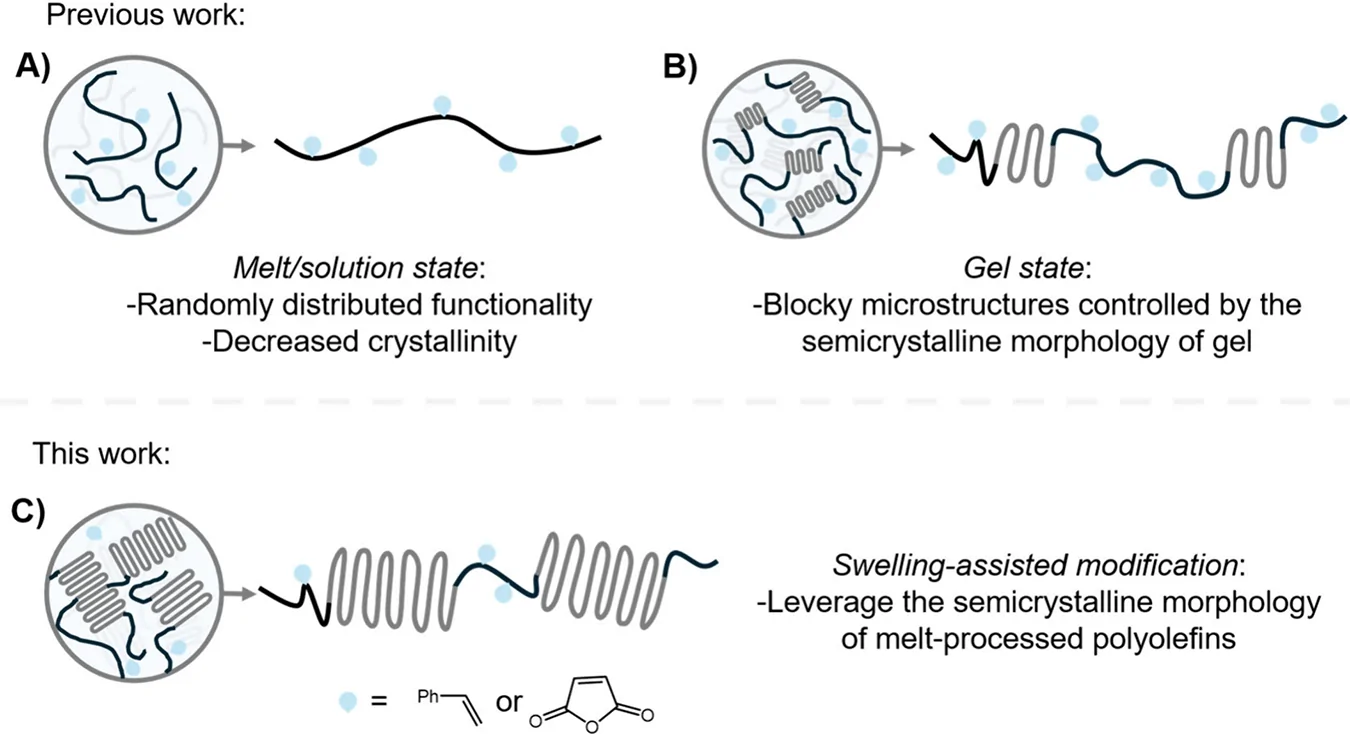
Schematic representation of postpolymerization functionalization strategies for polyolefins: (A) melt- and solution-state functionalization, which installs functional groups randomly along the polymer backbone; (B) gel-state functionalization, which yields blocky microstructures governed by the semicrystalline morphology of the gel; and (C) swelling-assisted modification, which achieves selective functionalization of the amorphous phase in melt-processed polyolefins.

In this work, we demonstrate a solvent swelling-assisted post-polymerization functionalization strategy that selectively targets the amorphous phase of bulk polyolefins with minimal disturbance to crystalline regions (Figure 1C). We started with the extrusion-grade isotactic polypropylene (PP) as the model system, which has a melting temperature of 158 °C and crystallinity of 49% (Figure S1). Melt-pressed PP films were submerged in xylene at temperatures ranging from 75 to 100 °C to evaluate their swelling behavior in the reaction solvent. Under these conditions, the samples exhibited isotropic swelling, with the dimensions expanded from 8.9 ± 0.8% at 75 °C to 15.4 ± 1.1% at 100 °C, indicating increased swelling ratios at elevated temperatures (Figure 2A). In-situ wide-angle X-ray scattering (WAXS) plots of the swollen PP show characteristic peaks at (110), (040), (130), and (111) (Figure S2).^25^ In-situ small-angle X-ray scattering (SAXS) (Figure 2B) reveals a primary ordering peak across all samples. Both results confirm that the semicrystalline structure was maintained in the swollen state, and the solvent molecules primarily penetrate and swell the amorphous domains.^26^ The long period increased from 13.9 nm for neat PP to 16.8 nm for swollen PP at 75 °C. At elevated temperatures of 85 °C and 95 °C, the long period further increases to 18.0 and 19.5 nm, corresponding to swelling degrees of 29% and 40%, respectively (Figure 2C). The apparent crystallinity of swollen PP as a function of temperature was determined from WAXS (Figure S2). This gradual decrease is consistent with the increase in long period observed over the same temperature range (Figure 2C), suggesting that the increase in swelling at elevated temperatures is attributed to partial melting of crystalline lamellae, which releases chain segments into the amorphous domains and increases the swelling-induced expansivity.^26–28^

**Figure 2:**
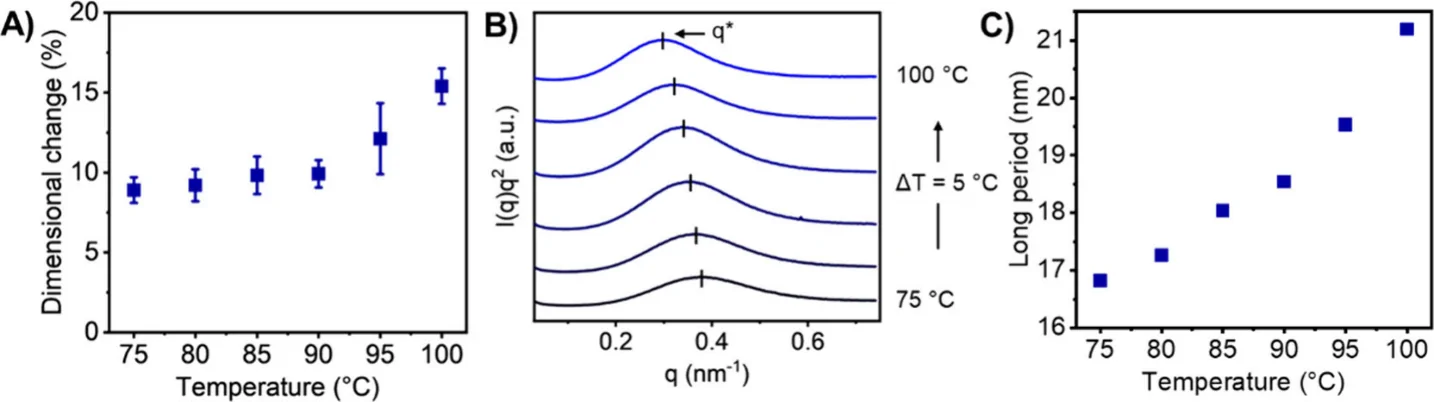
(A) Dimensional change of PP when immersed in xylene at different temperatures; (B) Lorentz-corrected in situ SAXS curves of PP in xylenes at different temperatures; and (C) Long period of swollen PP at different temperatures.

Styrene grafting using dicumyl peroxide (DCP) as the radical initiator was selected as a model system to establish proof of concept, given its well-understood chemistry that is nonetheless difficult to control in traditional melt-state functionalization.^29–31^ The proposed mechanism relies on the diffusion of DCP and styrene into the amorphous domains of solvent-swollen PP, followed by the thermal decomposition of DCP to generate cumyloxy radicals that abstract hydrogen atoms on the PP backbone, producing radicals on PP backbone that subsequently add to styrene to yield grafted chains.^32^ The functionalization was performed at 85 °C to maintain PP in a swollen state, preserving the lamellar crystallite morphology while allowing a practical rate of DCP decomposition (estimated half-life >7 h at this temperature).^33,34^ To remove residual styrene monomer and polystyrene homopolymer, the crude styrene-functionalized PP (PP-*g*-St) was purified by two cycles of dissolution in hot xylene followed by precipitation in dichloromethane. The ^1^H NMR spectrum of purified PP-*g*-St (Figure 3A) shows the characteristic signals of isotactic polypropylene alongside aromatic protons from styrene (6.6-7.1ppm). Combined analysis by heteronuclear single quantum coherence (HSQC, Figure S3) and heteronuclear multiple bond correlation (HMBC, Figure S4) spectroscopy reveals a correlation between the methyl carbon of PP (21ppm) and the tertiary C–H proton adjacent to the grafted styrene unit (2.2ppm), confirming covalent grafting of styrene onto the PP backbone. Kinetic study of styrene grafting on PP at 85 °C (styrene: 1 mL/mL xylene; DCP: 0.1 g/mL xylene) shows a steady increase in styrene content during the first 24 h, followed by a plateau after 48 h (Figures S5–S7). We attribute the slowdown of the reaction after 24 h to depletion of the radical initiator and increased viscosity arising from styrene homopolymerization. To establish comprehensive functionalization-material properties relationships, the degree of PP functionalization was systematically varied by adjusting styrene concentration in xylene. Fourier transform infrared (FT-IR) spectra of PP-*g*-St prepared at different styrene concentrations (0.3 to 2 mL/mL xylene) show a progressive increase in the characteristic aromatic C–H bending band at 750 cm^–1^, suggesting the increase of degree of functionality (Figure 3B). The styrene content in PP-*g*-St was further determined by ^1^H-NMR (Figures S8–S11) as weight percent relative to PP and plotted as a function of styrene concentration in xylene (Figure 3C). The styrene content in PP-*g*-St increases nonlinearly with monomer concentration, likely suggesting that potential homopolymerization at higher styrene loadings elevates the viscosity and limits reagent diffusion into the swollen polymer matrix. All PP-*g*-St samples showed molecular weights and dispersity values comparable to neat PP (Figure S12), indicating that chain scission and radical coupling side reactions were limited in our process. The direct comparison of GPC traces for samples that were functionalized in the solution and swollen states suggests that swelling-induced functionalization leads to more suppressed chain scission, which can be attributed to the lower reaction temperature required under swelling conditions, whereas a higher temperature (130 °C) is necessary for solution-state functionalization to maintain sufficient solubility. For the styrene system in this work, the highest functionalization achieved was 1.12 mol % (2.8 wt %), which is slightly lower compared to amidyl radical C–H functionalization in gel-state^23^ or solution-state.^35^ Our results reflect an inherent limitation of using styrene as the grafting monomer. Specifically, in the presence of DCP, styrene undergoes competing homopolymerization in addition to grafting onto the PP backbone, reducing the efficiency of monomer incorporation. These suggest that extending the swollen-state strategy to more efficient C–H functionalization chemistries can be a promising direction for achieving higher graft densities with improved selectivity.

**Figure 3:**
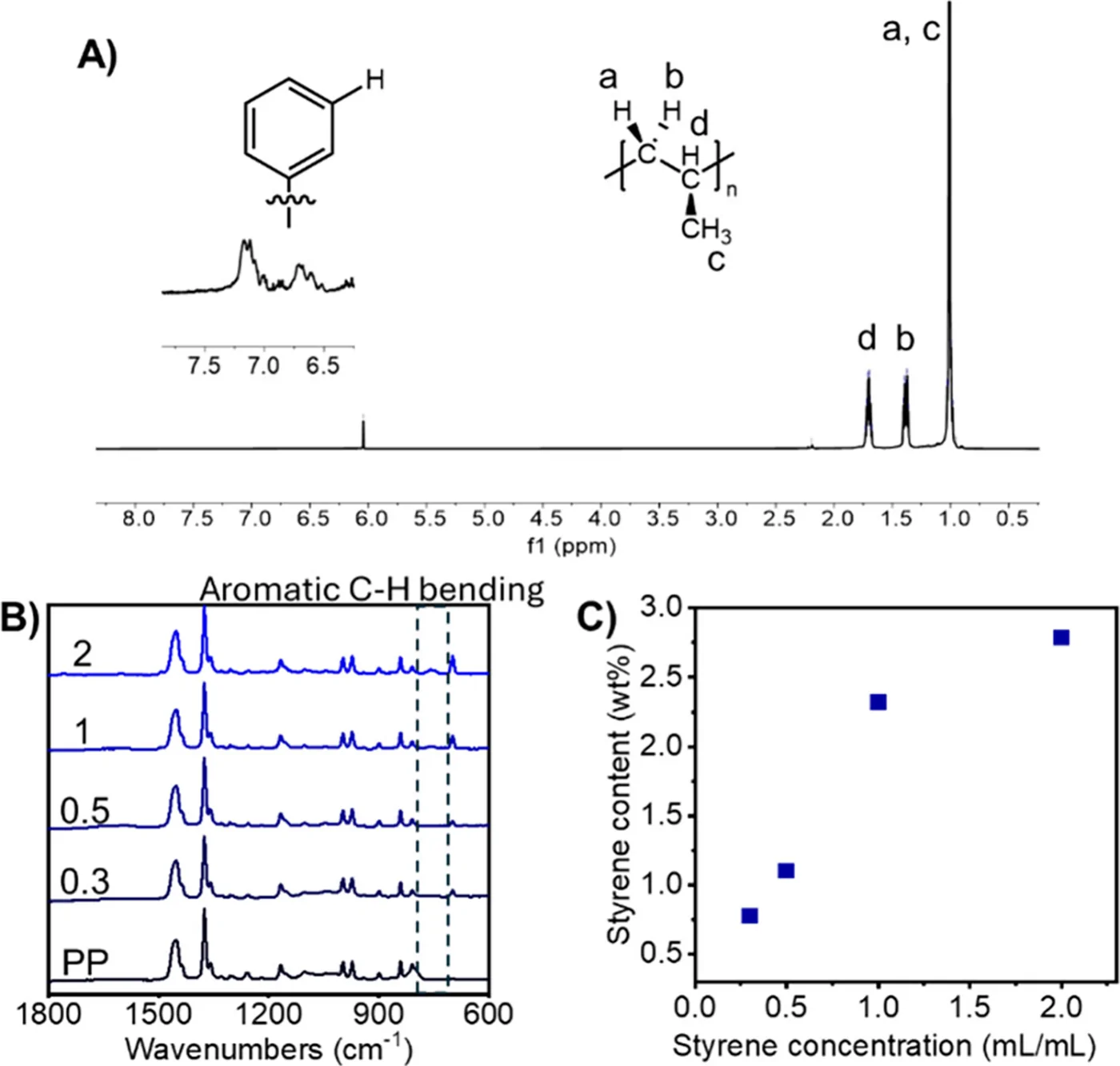
(A) ^1^H NMR of PP-*g*-St; (B) FT-IR spectra of PP-*g*-St prepared under different styrene concentrations (0.3 to 2 mL/mL xylene); (C) Styrene content of PP-*g*-St as a function of styrene concentration in the reaction.

We further examined the thermal and dynamic mechanical properties of the modified materials. PP-*g*-St exhibited a similar decomposition profile to neat PP (Figure S13). As shown in Figure 4A and Figure S14, differential scanning calorimetry (DSC) revealed that the melting temperature (*T*_m_) of all PP-*g*-St samples remained close to that of neat PP (∼158 °C), indicating that the lamellar thickness was similar upon functionalization, which is approximately 5.8 nm according to Gibbs–Thomson equation.^36,37^ WAXS plots of all PP-*g*-St with different styrene contents show characteristic peaks of neat PP (Figure S15). The overall crystallinity decreased by approximately 6% (from 49% to 43%) and remained independent of the degree of functionality after styrene content over 2.3 wt %. For comparison, functionalization of PP under homogeneous solution conditions at 130 °C (to fully dissolve the polymer), yielded a random copolymer with a comparable styrene content of 1.8 wt % (Figure S16) but reduced crystallinity (27%) and *T*_m_ (154 °C, Figure S17). To understand the impact of functionalization on the crystallization behavior, non-isothermal crystallization kinetics were analyzed using the Jeziorny-modified Avrami rate constant *K*′ for both solution- and swollen-state functionalized PP-*g*-St (Figures S18–S23).^38,39^ For both series, *K*′ decreased with increasing styrene content, consistent with pendant styrene groups acting as structural defects that shorten uninterrupted crystallizable PP sequences and disrupt lamellar packing. Notably, despite the higher styrene content (2.8 wt %), the swollen-state PP-*g*-St exhibited crystallization kinetics comparable to those of solution-state PP-*g*-St with only 1.8 wt % styrene. The retention of crystallization kinetics for the swollen-state functionalized PP-*g*-St is consistent with the proposed blocky microstructure.^23^ These results collectively support the hypothesis that swollen-state functionalization incorporates functional groups in a manner compatible with the semicrystalline morphology of melt-processed polyolefins.

**Figure 4:**
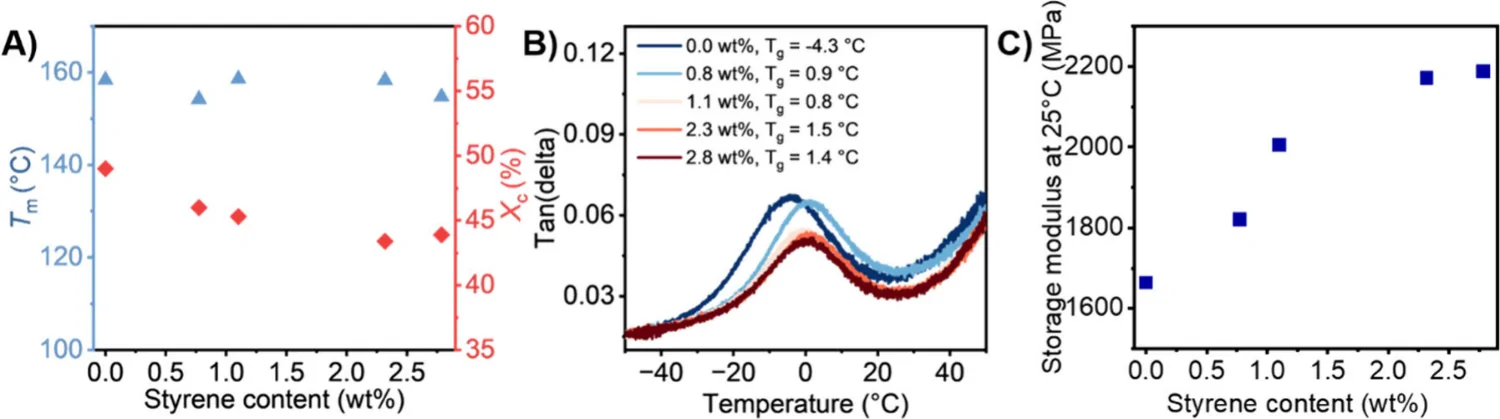
(A) Melting temperature (*T*_m_) and crystallinity (*X*_c_) of neat PP and PP-*g*-St obtained from DSC; (B) Tan *δ* and *T*_g_ of PP-*g*-St obtained from DMA; (C) Storage modulus as a function of styrene content obtained from DMA (PP with 0.0 wt % styrene was prepared by submerging PP in xylene under the same time and temperature conditions used for styrene functionalization).

As shown in Figure 4B, dynamic mechanical analysis (DMA) was performed to characterize the glass transition temperatures (*T*_g_) of these functionalized PP samples by extracting the peak position in tan δ, increased by 5–6 °C relative to PP (annealed in only xylene, styrene content = 0.0 wt %) for all PP-*g*-St samples. The limited change in the *T*_g_ across functionalized PP is anticipated due to relative low amount of styrene graft content, varying from 1.1 wt % to 2.8 wt %. This peak temperature shift occurred without significant peak broadening, indicating that styrene grafting occurs predominantly within the amorphous phase in a well-controlled manner, while preserving the structural integrity of the crystalline domains. With increasing graft density, the storage modulus increased while tan δ decreased (Figure 4C and Figure S24), consistent with restricted segmental mobility of PP chains imposed by the grafted styrene units. To investigate the impact of styrene grafting on the mechanical properties, PP-*g*-St with varying styrene contents were subjected to uniaxial tensile testing (Figure S25). Notably, PP-*g*-St retained a strain at break (∼1200%) and Young’s modulus (∼290 MPa) comparable to PP (annealed in only xylene, styrene content = 0.0 wt %), regardless of the degree of functionalization. These results indicate that the primary, load-bearing crystalline network remains intact under swollen-state modification, preserving the characteristic mechanical stiffness of PP.

To assess the generality of the swollen-state functionalization approach, we extended the method to high-density polyethylene (HDPE) using maleic anhydride (MA) as the grafting monomer and benzoyl peroxide (BPO) as the radical initiator (Figures S26–S38). Because both PP and HDPE exhibit reduced swelling in MA/xylene solutions relative to pure xylene (Figures S26 and S27), the degree of functionalization was instead modulated by varying BPO concentration. As shown in Figures S28 and S29, MA content in the modified PP and HDPE increased with BPO concentration, reaching up to 0.8 wt % for PP-*g*-MA and 2.1 wt % for HDPE-*g*-MA, the values of which are comparable to those of commercial maleated polyolefins.^40,41^ The modified PP and HDPE exhibited similar melting transitions to their unmodified counterparts during the first DSC heating cycle (Figure S30), with no significant changes in peak melting temperature (Figure S31). After recrystallizing from the melt, the degree of crystallinity measured from the second heating cycle decreased modestly (by ∼5% for PP-*g*-MA and ∼8% for HDPE-*g*-MA at the highest MA contents). Moreover, water contact angle (WCA) measurements were performed to evaluate the impact of MA grafting on the hydrophilicity of the materials (Figure S32). Neat PP and HDPE exhibited WCA values of 138.5° and 157.8°, respectively, which decreased progressively to ∼116.6° for PP-*g*-MA (0.75 wt % MA) and 119.1° for HDPE-*g*-MA (2.06 wt % MA), demonstrating that surface hydrophilicity can be tuned by controlling MA graft density. To further increase functional group loading content, we performed repeated sequential swelling cycles on both PP-*g*-MA and HDPE-*g*-MA (Figure S36). MA content increased steadily with each swelling cycle, reaching 4.5 wt % after five cycles for PP-*g*-MA (BPO = 0.2 g/mL) and 1.9 wt % for HDPE-*g*-MA (BPO = 0.05 g/mL. PP-*g*-MA after each sequential swelling exhibited melting transitions and crystallinity comparable to unmodified PP during the first DSC heating cycle (Figure S37), except at the highest MA loading (fifth cycle, BPO = 0.2 g/mL), which displayed a bimodal crystallization exotherm and reduced crystallization peak temperature; this result may reflect disrupted lamellar ordering at high MA loadings or chain scission from repeated thermal processing.

We further assessed the generalizability of our method with respect to functional group identity by grafting four distinct functional monomers onto both PP and HDPE (Figure 5A–D). All functionalized samples were purified by multiple precipitation cycles and characterized by ^1^H NMR (Figures S39–S43) and FT-IR (Figures S44–S48) to confirm grafting and quantify graft density. As shown in Figure 5D, 4-hydroxy-TEMPO grafted PP (PP-*g*-TEMPO, 1.2 mol %) exhibited a similar *T*_m_ to neat PP and a modest 7% decrease in crystallinity. Although HDPE reached a higher degree of functionality than PP for all four functional groups examined, all functionalized HDPE retained melting transition similar to the neat HDPE (Figure 5E). While functionalized HDPE showed a noticeable decrease in crystallinity relative to neat HDPE (*X*_c_ = 87%), the resulting values remained within the range reported for HDPE.^42,43^ Overall, these results demonstrate that the swollen-state functionalization strategy is broadly applicable across functional group identity, underscoring its versatility as a platform for polyolefin modification.

**Figure 5:**
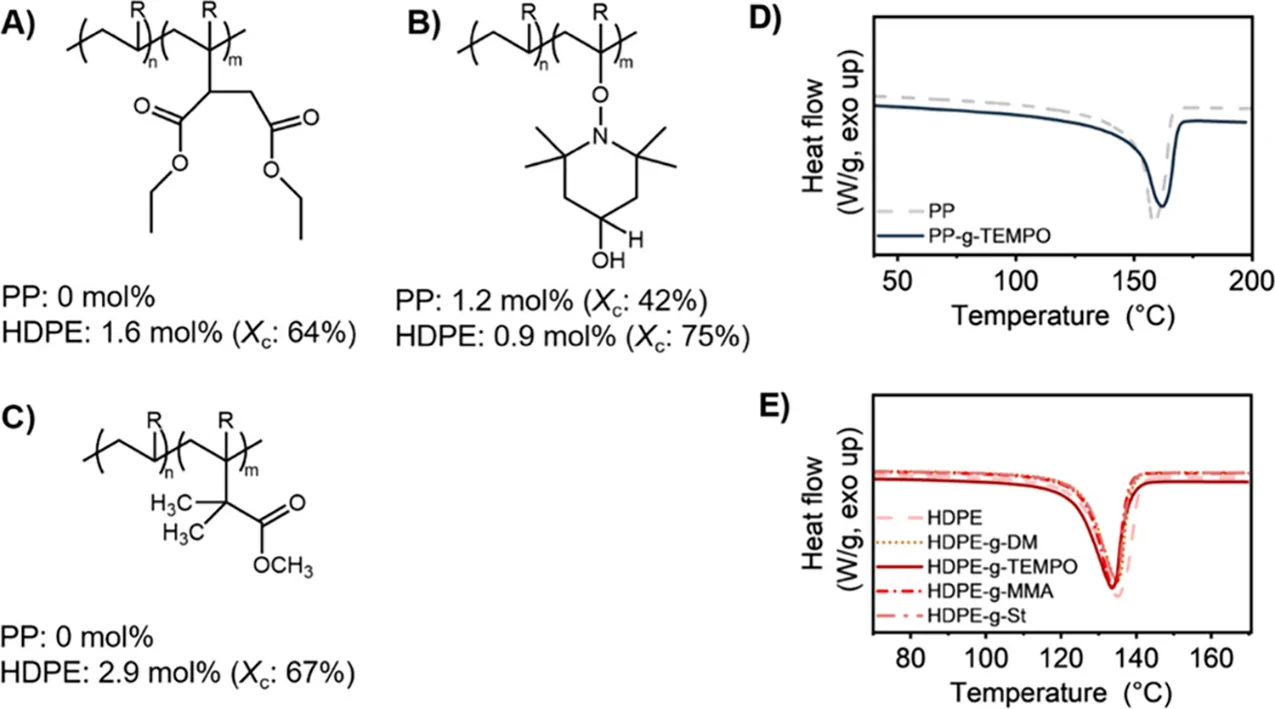
PP and HDPE functionalized with (A) diethyl maleate (DM), (B) 4-hydroxy-TEMPO (TEMPO), and (C) methyl methacrylate (MMA), as well as melting endotherms of the second heat of DSC for functionalized (D) PP and (E) HDPE.

In summary, we have demonstrated a swelling-assisted post-polymerization functionalization in the semicrystalline state of polyolefins that allowed selective modification in the amorphous regions. By leveraging the steric protection of the crystallite from melt-processed polyolefins, functional groups were grafted onto the polymer backbone exclusively within the amorphous phase, yielding functionalized materials that retained the melting temperature, degree of crystallinity, and mechanical properties of their unmodified counterparts. The approach is broadly applicable across polyolefin substrates and functional monomers. The degree of functionality can be controlled by sequential swelling cycles and concentrations of the reactants. Compared to existing post-polymerization modification methods, this strategy offers a versatile platform for producing functional polyolefins with preserved semicrystalline microstructures and bulk properties.

## Supplementary Material



## Notes

Experimental section containing materials list, sample presentation and material characterization; DSC curves of functionalized polyolefins; WAXS plots of PP in xylene at increasing temperature; HSQC and HMBC of PP-*g*-St; ^1^H NMR of functionalized polyolefins; kinetic study of styrene grafting; HT-GPC curves of PP-*g*-St; Avrami analysis of neat PP, PP-*g*-St from swollen state and solution state functionalization; DMA data; Stress–strain curves; WAXS and SAXS plots of PP-*g*-St with different styrene content; Temperature effect on MA grafting on PP and HDPE; FT-IR of functionalized polyolefins; Images of water contact angle measurements; Kinetic study of MA grafting; Incremental MA content by sequential swelling; TGA of MA grafted PP and HDPE (PDF)
