# Supplementary material for: Targeted Chemical Functionalization of the Amorphous Phase in Polyolefins via Controlled Solvent Swelling
Source: ACS Macro Lett. 2026 Aug 31;15(9):1355–61. doi: 10.1021/acsmacrolett.6c00383 (PMC13585136; doi:10.1021/acsmacrolett.6c00383)
Supplement: Supplementary file 1 [file mz6c00383_si_001.pdf]

Supporting Information For:

Targeted Chemical Functionalization of the Amorphous Phase in Polyolefins via Controlled Solvent Swelling

Jin Qian [a], Tianli Ren [b], Peiran Wei [c], Wisdom Kemakolam [a], Zhe Qiang[,a]*

[a] School of Polymer Science and Engineering, The University of Southern Mississippi, 118 College Drive, Hattiesburg, MS 39406

[b] Mississippi Polymer Institute, The University of Southern Mississippi, Hattiesburg, MS 39402

[c] Soft Matter Facility, Department of Materials Science and Engineering, Texas A&M University, College Station, Texas 77845, United States

Experimental Section

Materials

Polypropylene (extrusion-grade, Mw ~ 640,000 g/mol, Đ: ~6.4) was obtained from Muehlstein. High-density polyethylene (HDPE, Mw ~ 114,000 g/mol, Đ: ~9.9) was obtained from NOVA Chemicals (SURPASSHPs667). Xylene (99%) and dichloromethane (DCM, 99%) were obtained from Thermo Fisher Scientific. Dicumyl peroxide (DCP, 98%), benzoyl peroxide (BPO, Luperox® A75FP), styrene (99%), maleic anhydride (MA, 99%) , diethyl maleate (DM, 97%), 4-hydroxy-TEMPO (TEMPO, 95%), and methyl methacrylate (MMA, 99%) were obtained from Millipore Sigma and used as received.

Sample preparation

Polymer pellets were placed into rectangular molds (thickness = 0.75 mm) and compression molded with a Carver press at 5000 psi for 5 min at 190 ºC, then cooled on an aluminum bench with a steel heat sink on top to hold the mold together.

For the swelling ratio measurements, the width and thickness of each film were measured with calipers, and the mass of each tensile bar after swelling was recorded. Samples were submerged in xylenes and held at a temperature between 75 to 100 ºC for 24 h. Upon removal from xylenes, the dimensions of the gauge were measured again. Each film was dried in a vacuum oven at 80 ºC for 18h before the mass was recorded.

Styrene functionalization of polypropylene:

In a typical styrene functionalization protocol, styrene (0.3, 0.5, 1 or 2 mL per mL of xylene), DCP (0.1g/mL xylene), and xylene were combined in a vial and stirred at room temperature until the DCP completely dissolved. PP films were submerged in the solution mixture and heated at 85 °C for 24 h. Following the reaction, the functionalized PP films were taken out, dissolved in xylene at 130 °C, and precipitated into dichloromethane (DCM) to extract styrene homopolymer. The solid PP was recovered via filtration, and this dissolution–precipitation cycle was repeated twice. The purified polymer was then dried in a vacuum oven at 60 °C before characterization.

For mechanical and physical property evaluations (tensile testing, DMA, and X-ray scattering), melt-pressed PP films were first punch-pressed into Type V tensile bars (w = 3.25 mm, t = 0.75 mm, l = 12.25 mm). These bars were subjected to identical reaction conditions (85 °C for 24 h). Following the reaction, the functionalized PP tensile bars were removed from the reaction mixture and washed to extract styrene homopolymer by submerging them in a 95 °C xylene bath, which was replaced ten times over 48 h. The purified bars were dried under vacuum at 80 °C for 18 h before testing. Thermogravimetric analysis (TGA) was performed on the dried samples (10 °C/min heating rate under N_2 up to 600 °C) to confirm the complete removal of residual xylene.

Styrene functionalization of polypropylene under solution state:

PP pellets (2g) were dissolved in xylene (100mL) at 120 °C. DCP (0.06g) and styrene (0.06 mL) were added to the PP/xylene solution. The mixture was stirred at 120 °C for 24h before being

poured into DCM for precipitation. The solid PP was recovered via filtration, and this dissolution–precipitation cycle was repeated twice. The purified polymer was then dried in a vacuum oven at 60 °C before characterization. The styrene content of the functionalized PP was 1.8 wt% as determined from ^{1}H-NMR.

PP pellets (2g) were dissolved in xylene (100mL) at 120 °C. DCP (0.07g) and styrene (0.5 mL) were added to the PP/xylene solution. The mixture was stirred at 120 °C for 24h before being poured into DCM for precipitation. The solid PP was recovered via filtration, and this dissolution–precipitation cycle was repeated twice. The purified polymer was then dried in a vacuum oven at 60 °C before characterization. The styrene content of the functionalized PP was 5 wt% as determined from ^{1}H-NMR.

Maleic anhydride functionalization of polyolefins:

In a typical MA functionalization protocol, MA (1g/mL xylene), BPO (0.05, 0.1, 0.2 or 0.3 g/mL xylene), and xylene were combined in a vial at the specified concentrations. The polymer films were submerged in the mixture and held at the target temperatures for 24 h. Following the reaction, the functionalized polymer films were taken out from the reaction mixture and washed by submerging them in acetone at 50 °C to extract any unreacted MA. The acetone

bath was replaced three times over a 2 h period. The purified polymer was dried in a vacuum oven at 60 °C.

The MA content of the functionalized polymers was determined via titration. Polymer (~0.1 g) was refluxed in xylenes (~20 mL) at 130 °C until completely dissolved. 3 drops of water were then added to the hot polymer/xylene solution to hydrolyze the anhydride, followed by titration with 0.025 M KOH solution, using phenolphthalein as the indicator. The MA content (G), in weight percent, was calculated via the following equations:

$$A.N. = \frac{(V)(M)(M_{\mathrm{n,KOH}})}{m_{\mathrm{poly}}} \tag{1}$$

$$G = \frac{(\frac{A.N.}{1000})(M_{n,MA})}{2M_{n,KOH}} \times 100\% \tag{2}$$

where A.N. is the acid number (the mass of KOH (unit: mg) required to neutralize 1 g of polymer). V is the volume of KOH titrant added, M is the molarity of the titrant, M_n is the molar mass of the specified component, and m_{poly} is the mass of polymer used in the analyte. Note that $M_{n,KOH}$ is multiplied by two in eq 2 to account for the dual acidic groups generated per MA unit upon hydrolysis.

Diethyl maleate functionalization of polyolefins:

Diethyl maleate (1g/mL xylene), BPO (0.1 g/mL xylene), and xylene were combined in a vial. The polyolefin films were submerged in the mixture and held at 85 C for 24 h. Following the

reaction, the functionalized polyolefin films were taken out, dissolved in xylene at 130 °C, and precipitated into dichloromethane (DCM) to extract styrene homopolymer. The solid polyolefin was recovered via filtration, and this dissolution-precipitation cycle was repeated twice. The purified polymer was then dried in a vacuum oven at 60 °C before characterization.

4-hydroxy-TEMPO functionalization of polyolefins:

4-hydroxy-TEMPO (1g/mL xylene), BPO (0.1 g/mL xylene), and xylene were combined in a vial. The polyolefin films were submerged in the mixture and held at 85 C for 24 h. Following the reaction, the functionalized polyolefin films were taken out, dissolved in xylene at 130 °C, and precipitated into dichloromethane (DCM) to extract styrene homopolymer. The solid polyolefin was recovered via filtration, and this dissolution-precipitation cycle was repeated twice. The purified polymer was then dried in a vacuum oven at 60 °C before characterization.

Methyl methacrylate functionalization of polyolefins:

Methyl methacrylate (0.5g/mL xylene), DCP (0.1 g/mL xylene), and xylene were combined in a vial. The polyolefin films were submerged in the mixture and held at 85 C for 24 h. Following the reaction, the functionalized polyolefin films were taken out, dissolved in xylene at 130 °C, and precipitated into dichloromethane (DCM) to extract styrene homopolymer. The solid polyolefin was recovered via filtration, and this dissolution-precipitation cycle was repeated twice. The purified polymer was then dried in a vacuum oven at 60 °C before characterization.

Styrene functionalization of high-density polyethylene:

Styrene (1 g/mL xylene), DCP (0.1 g/mL xylene), and xylene were combined in a vial. The HDPE films were submerged in the mixture and held at 85 C for 24 h. Following the reaction, the functionalized HDPE films were taken out, dissolved in xylene at 130 °C, and precipitated into dichloromethane (DCM) to extract styrene homopolymer. The solid HDPE was recovered via filtration, and this dissolution-precipitation cycle was repeated twice. The purified polymer was then dried in a vacuum oven at 60 °C before characterization.

Materials Characterization

The melting temperatures (T_m) of samples before and after functionalization were determined using differential scanning calorimetry (DSC) with a TA Instruments Discovery DSC250. Tzero pans and lids from TA Instruments were used, and a heat-cool-heat cycle was employed with a temperature profile of 0 to 200 ºC with a ramp rate of 10 ºC/min. The degree of crystallinity (X_c) before and after functionalization was calculated using the following equation:

$$X_c = \left(\frac{\Delta H_f}{\Delta H_f^0}\right) \times 100\% \quad (3)$$

where ΔH_f is the enthalpy of fusion and ΔH_f^0 is the standard enthalpy of fusion (PP: ΔH_f^0=209 J/g; HDPE: ΔH_f^0=293 J/g).

The lamellar thickness of PP crystals was calculated with the Gibbs–Thomson equation,

$$T_m = T_m^0\left(1 - \frac{2\sigma_e}{\Delta H_f^0 L}\right) \quad (4)$$

where T_m^0 is the equilibrium melting temperature of the crystalline lamella (T_m^0 = 481 K), T_m is the measured melting temperature via DSC, ΔH_f^0 is the enthalpy of fusion of the crystalline phase of PP (ΔH_f^0= 207 J/g), and σ_e is the surface energy of the basal surface of a lamellar crystal (σ_e = 62.3 × 10^{-7} J/cm^2).[1,2]

For non-isothermal crystallization kinetics, the samples were first heated to 180 °C at 20 °C/min and held isothermally for 1 min to erase the thermal history. Then the sample was cooled to 20 °C at cooling rates (Φ). The cycle was repeated five times with Φ = 2, 5, 10, 20, and 40 °C/min.

The Avrami model can be extended to analyze non-isothermal crystallization using eq. 5.

$$X(t) = 1 - \exp(-K' t^{n'}) \quad (5)$$

where *K*' and *n*' are the Avrami constant and the Avrami exponent for non-isothermal crystallization, respectively, and the crystallization time (*t*) is,

$$t = \frac{T_{onset} - T}{\phi} \quad (6)$$

where T_{onset} is the onset temperature of the crystallization process and ϕ is the cooling rate. Due to the crystallization kinetic measurements being non-isothermal, it has been established that *K* should be corrected to obtain the Jeziorny-modified Avrami constant (*K*') at a normalized cooling rate using eq.7.

$$\log K' = \frac{\log K}{\phi} \tag{7}$$

High-temperature nuclear magnetic resonance (HT-NMR) spectroscopy was performed using a Bruker 600 MHz spectrometer equipped with a 10mm high-temperature broadband observe probe. Polyolefin samples (~20 mg) were dissolved in 0.6 mL of 1,1,2,2-tetrachloroethane-*d2* (TCE-*d2*) in a standard 5 mm outer diameter NMR tube. The sample tubes were heated in a dry block at 120 °C for 18 hours for complete polymer dissolution. All NMR measurements were performed at a constant probe temperature of 120 °C. Data processing was performed using TopSpin.

Fourier transform infrared (FT-IR) spectroscopy was performed using a Thermo-Nicolet iS50 FT-IR spectrometer with a smart iTX attachment. Scans were taken from 4000 to 600 cm^{-1} at a resolution of 4 cm^{-1} and an average of 32 scans.

Tensile bars were prepared using the same melt press procedures as sample preparation and punch-pressed into Type V tensile bars (w = 3.25 mm, t = 0.75 mm, l = 12.25 mm). Tensile tests of annealed and control samples were performed on a Mark-10 EasyMESUR motorized test stand with wedge grips and a 250 N load cell. The extension rate was held at 22 mm/min at room temperature.

Dynamic mechanical analysis (DMA) was performed using a TA Instruments discovery850 Dynamic Mechanical Analyzer with a single cantilever clamp and ACS3 cooling

accessory. The specimens were heated at a rate of 1 °C/min and at a frequency of 1 Hz and amplitude of 20 um with a temperature range of -60 to 60°C.

Both small-angle and wide-angle X-ray scattering (SAXS and WAXS) were performed using a Xeuss 3.0 system (Xenocs, Grenoble, France) equipped with a GeniX 3D beam delivery system, providing monochromatic Cu Kα radiation. Scattering patterns were collected by using a Dectris Eiger 2R 1M-pixel 2D detector and reduced into an integrated 1D profile. The sample-to-detector distance was set to 0.045 m for WAXS and 0.9 m for SAXS measurements. For in situ scattering experiments, small sections of melt-pressed samples were soaked in xylene, sealed in 1.5 mm diameter capillaries, and loaded into a Linkam stage. The temperature was increased at a rate of 20 °C/min and held for 1 h prior to recording measurements. The samples were exposed to the X-ray beam for 300 and 900 s for WAXS and SAXS measurements, respectively. Static SAXS measurements were performed at the 5-ID-D (DND-CAT) beamline of the Advanced Photon Source at the Argonne National Laboratory. A sample to detector distance of 8.5 m and an exposure time of 0.5 s were used for all measurements. Two-dimensional scattering patterns were collected with a MarMosaic 225 detector, azimuthally averaged, and reduced to one-dimensional data using software developed by beamline scientists at 5-ID-D. The long period (d), containing both the crystalline and amorphous regions of the polyolefins, was calculated using the following equation:

$$d = \frac{2\pi}{q *} \tag{8}$$

where q^* corresponds to the position of primary scattering peak.

The apparent crystallinity of swollen PP and HDPE was quantified from in situ WAXS by Gaussian deconvolution of the crystalline reflections and the amorphous halo. Because the diffuse scattering of liquid xylene contributes to the amorphous halo and cannot be subtracted, crystallinity at each temperature was compared against that of the swollen sample at 30 °C rather than against dry PP, so that the xylene scattering contribution cancels and the relative change in crystallinity reflects temperature-induced changes in polymer crystallinity alone.

For water contact angle measurements (WCA), polymer samples were dissolved in xylenes to form 2 wt % solutions, which were then drop-cast onto silicon wafers. The resulting films were dried at room temperature for 18h before vacuum drying at room temperature for 24 h. Water contact angle was measured using 4–6 μL water droplets with a Ramé-Hart 200–00 Std. goniometer. An ImageJ Drop Analysis was used to analyze the droplets and obtain static contact angle measurements. All materials were analyzed with the water contact angle measured in triplicate.

High-temperature size exclusion chromatography (HT-GPC) was performed on an HLC-8321GPC/HT system (Tosoh Bioscience, USA) equipped with a refractive index detector and two TSKgel GMHHR-H(20) HT2 columns. The measurements were conducted at 160 °C using 1,2,4-trichlorobenzene as the mobile phase at a flow rate of 1 mL min^{-1}. Polymer samples were prepared at a concentration of 4 mg mL^{-1} and heated at 160 °C for 2 h prior to analysis to ensure complete dissolution. Butylated hydroxytoluene (BHT, 0.1 wt%) was added as a

stabilizer to suppress polymer degradation at elevated temperature. Molecular weights were reported relative to polystyrene standards.

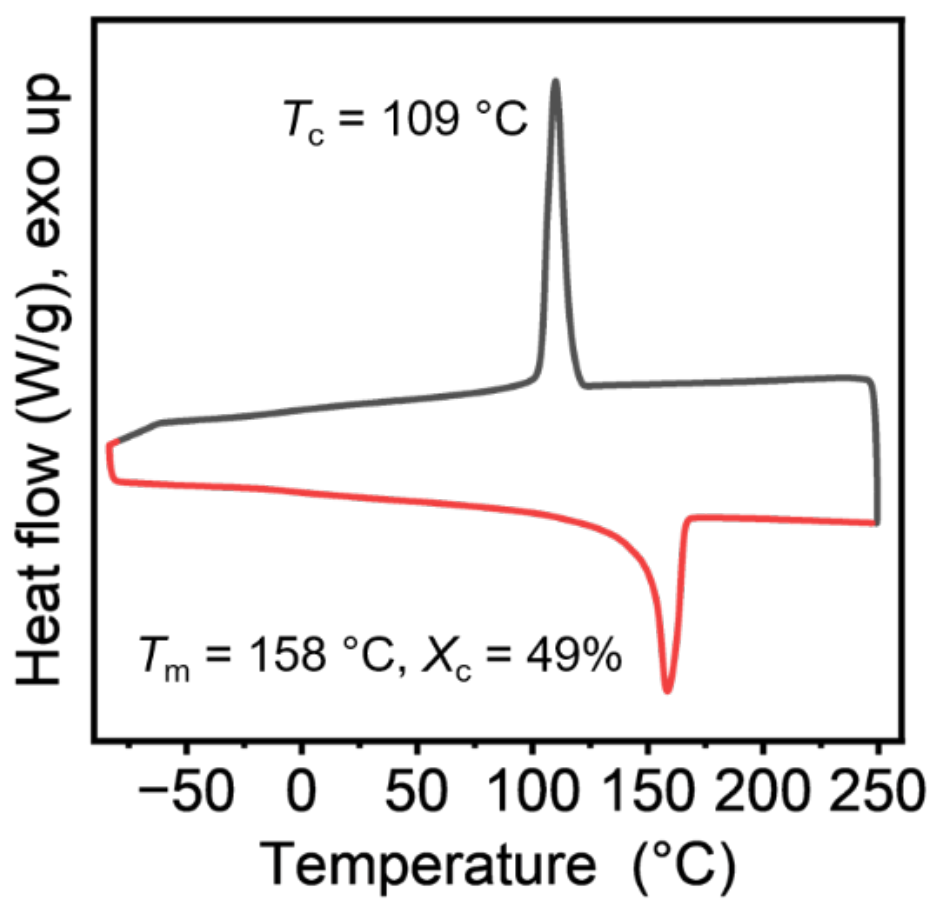

Figure S1. DSC thermograms of the cooling and the second heating cycle at a scan rate of 10 °C/min for neat PP.

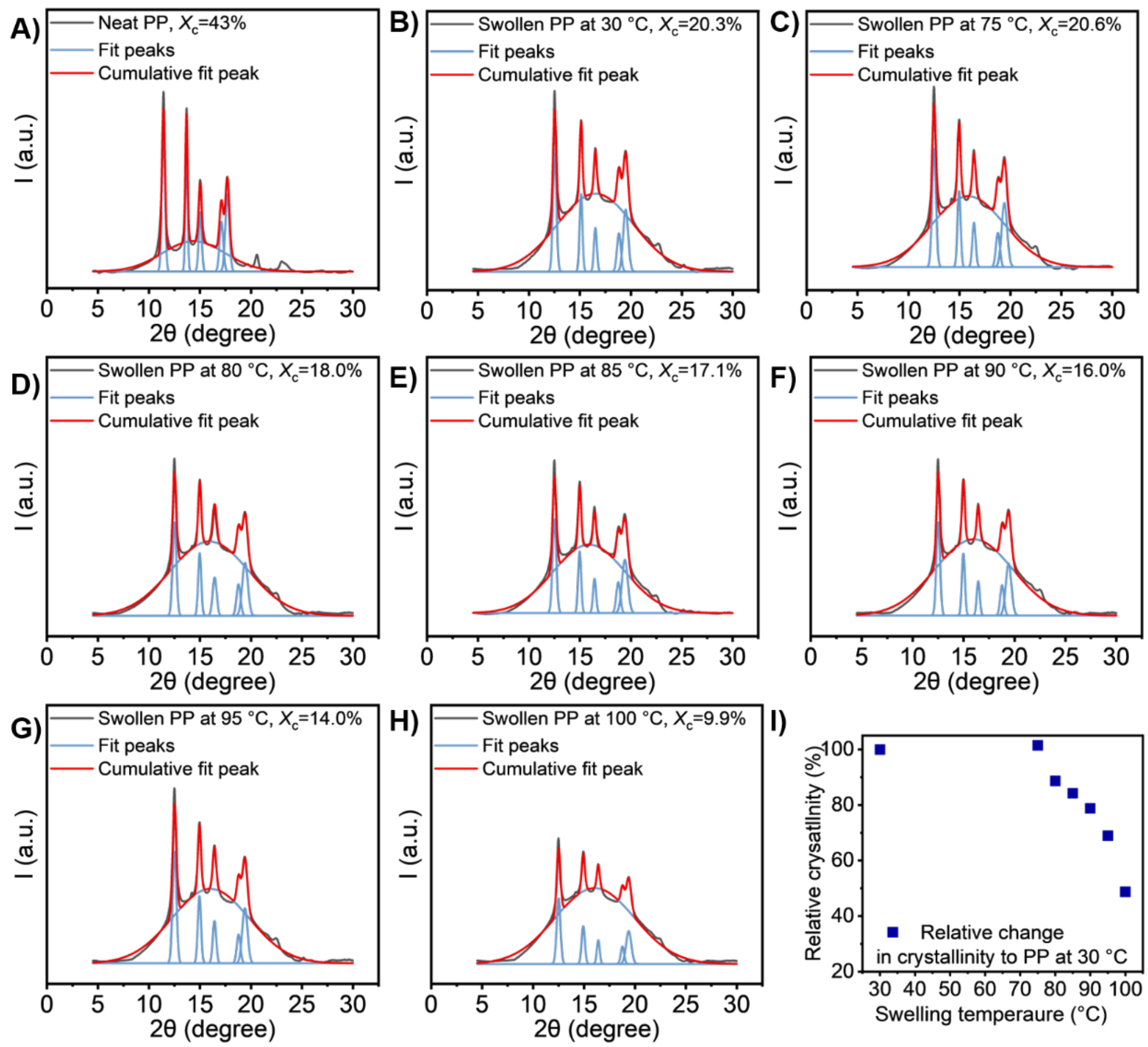

Figure S2. Deconvolution of the crystalline reflexes and the amorphous halo (Gaussian fit) for A) neat PP; B) to H) swollen PP at 30 °C to 100 °C; and I) relative change in crystallinity compared to the swollen PP at 30 °C.

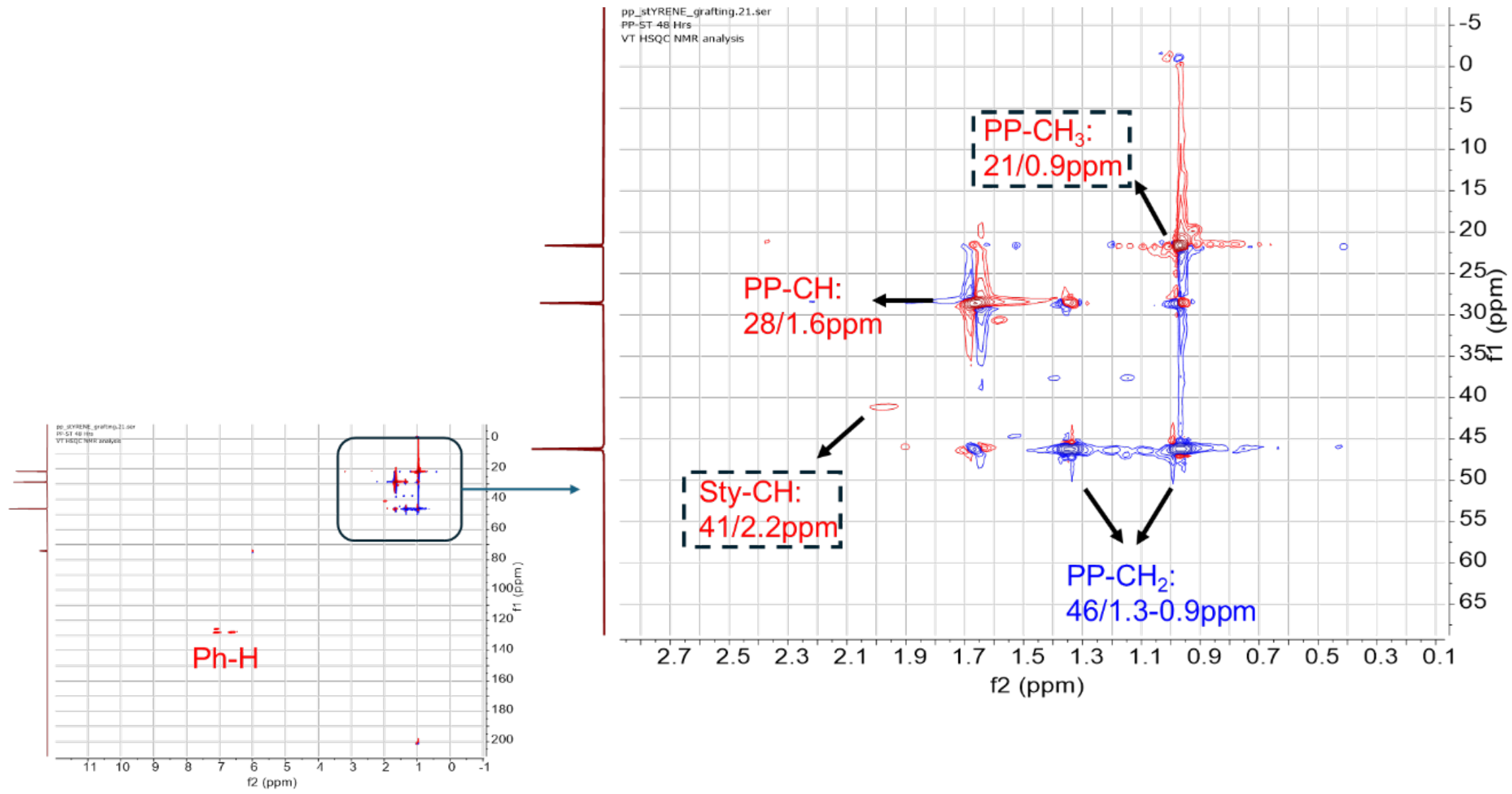

Figure S3. Heteronuclear single quantum coherence (HSQC) of PP-g-St modified with 1mL styrene/mL xylene, DCP=0.1g/mL.

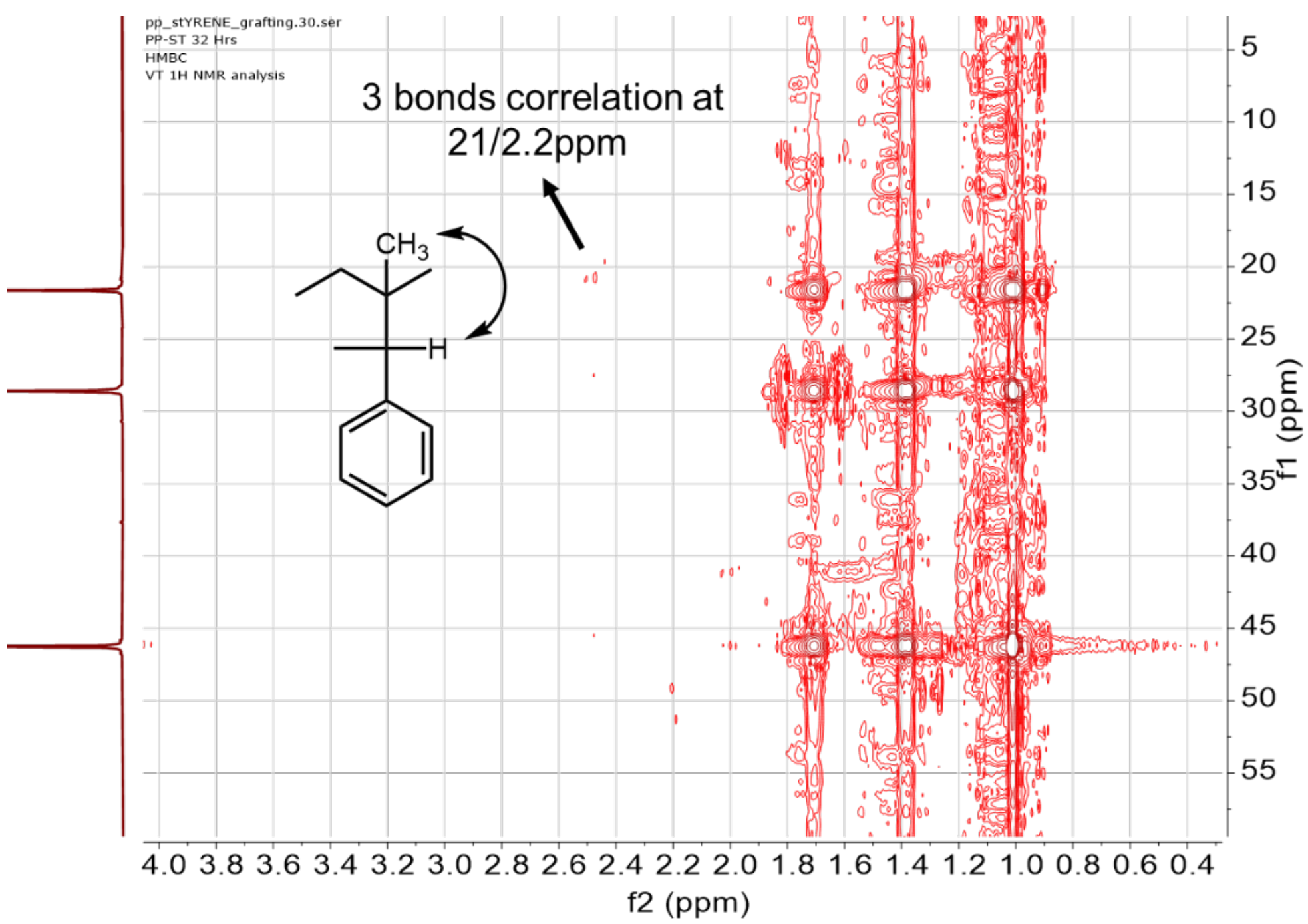

Figure S4. Heteronuclear multiple bond correlation (HMBC) of PP-g-St modified with 1mL styrene/mL xylene, DCP=0.1g/mL.

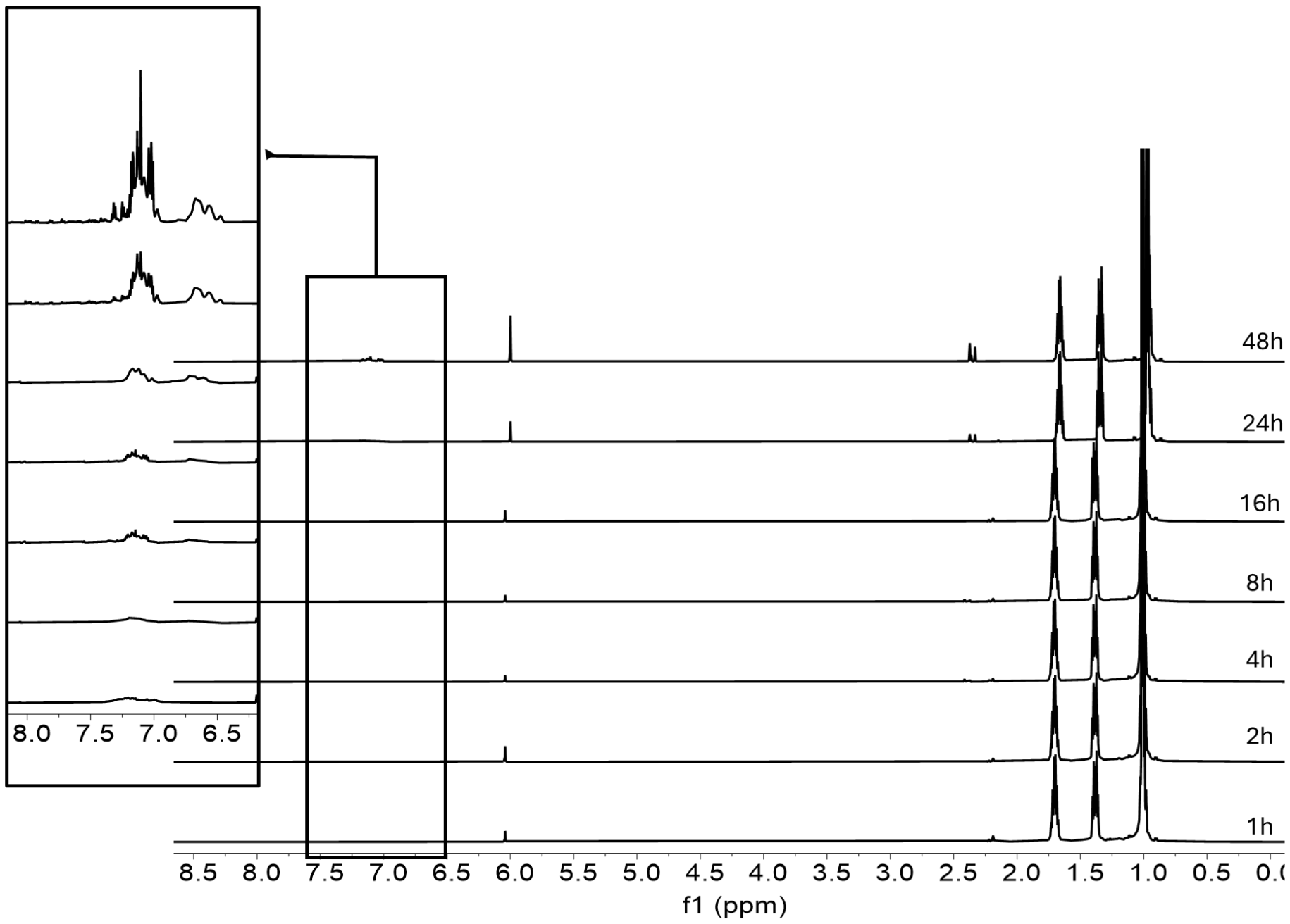

Figure S5. ^{1}H-NMR of grafting styrene on PP at 85 °C with styrene=1mL/mL xylene and DCP=0.1g /mL xylene at different temperatures.

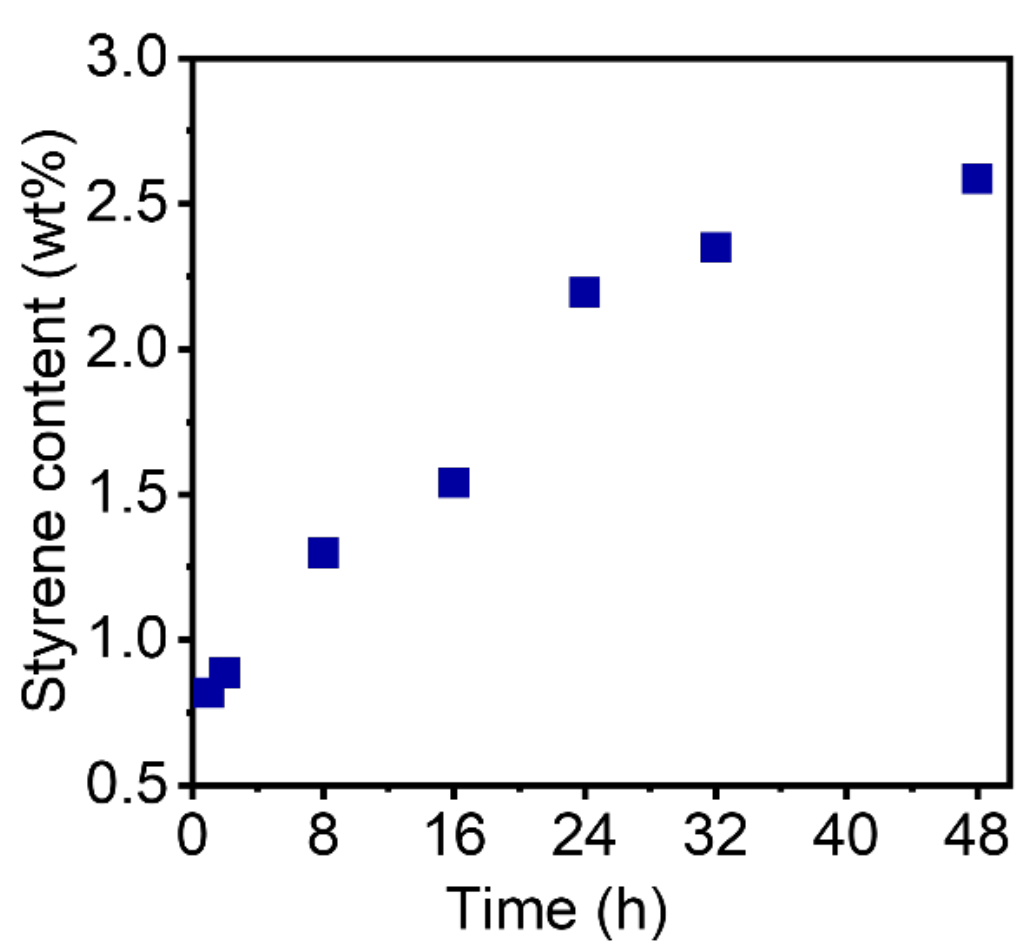

Figure S6. Kinetic study of grafting styrene on PP at 85 °C with styrene=1mL/mL xylene and DCP=0.1g /mL xylene.

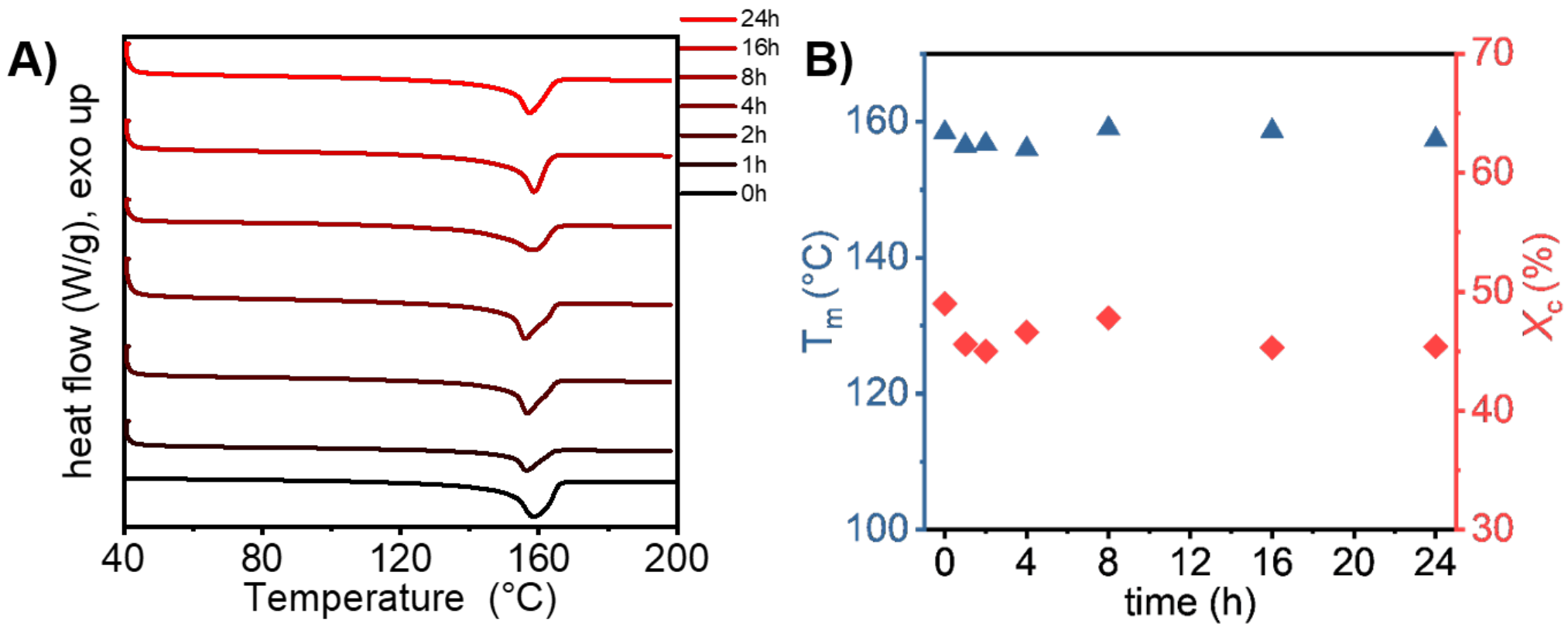

Figure S7. A) DSC thermograms of the second heating cycle at a scan rate of 10 °C/min, and B) T_m and crystallinity for kinetic studies of styrene functionalization

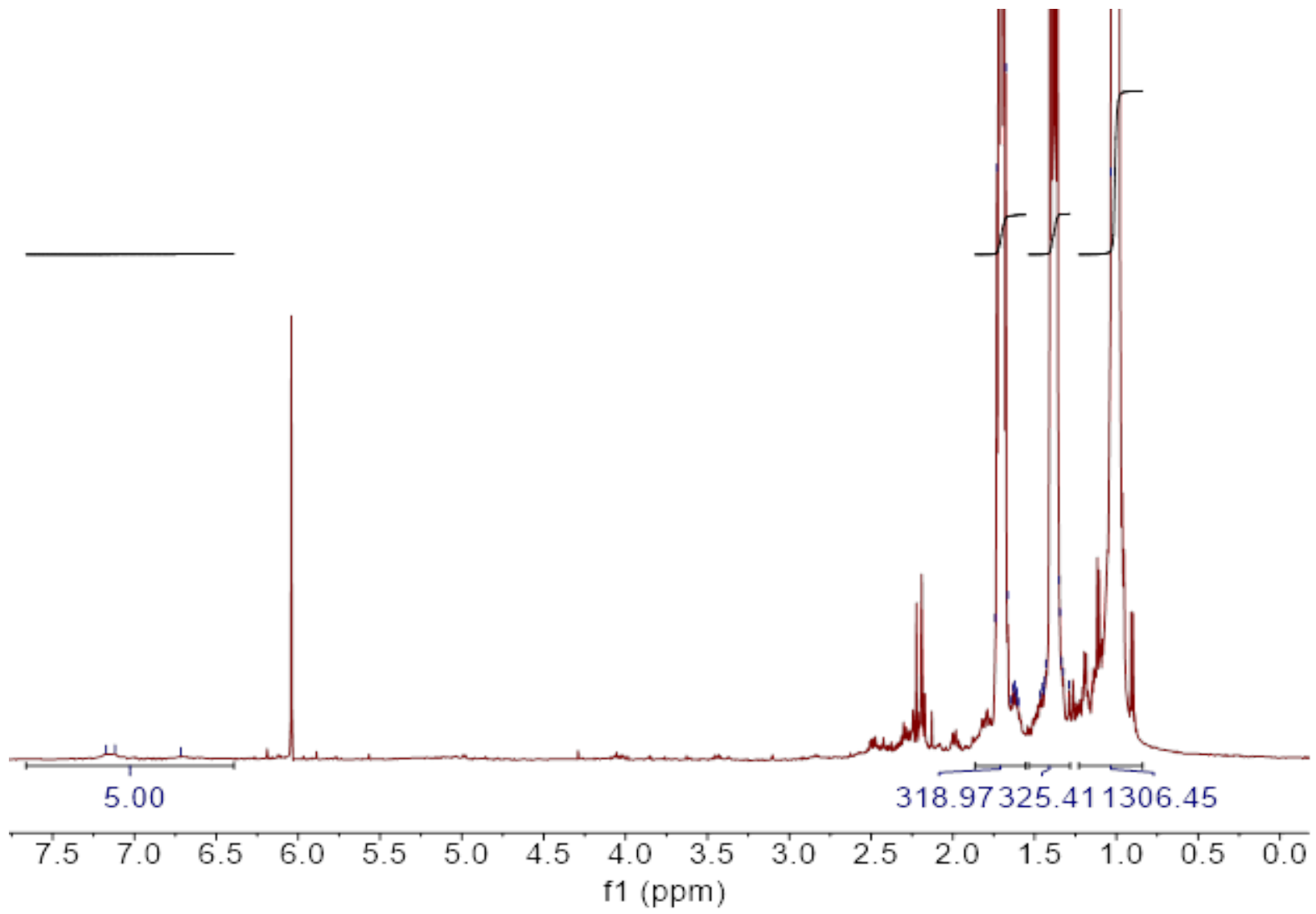

Figure S8. ^{1}H-NMR of PP-g-St modified with 0.3mL styrene/mL xylene, DCP=0.1g/mL.

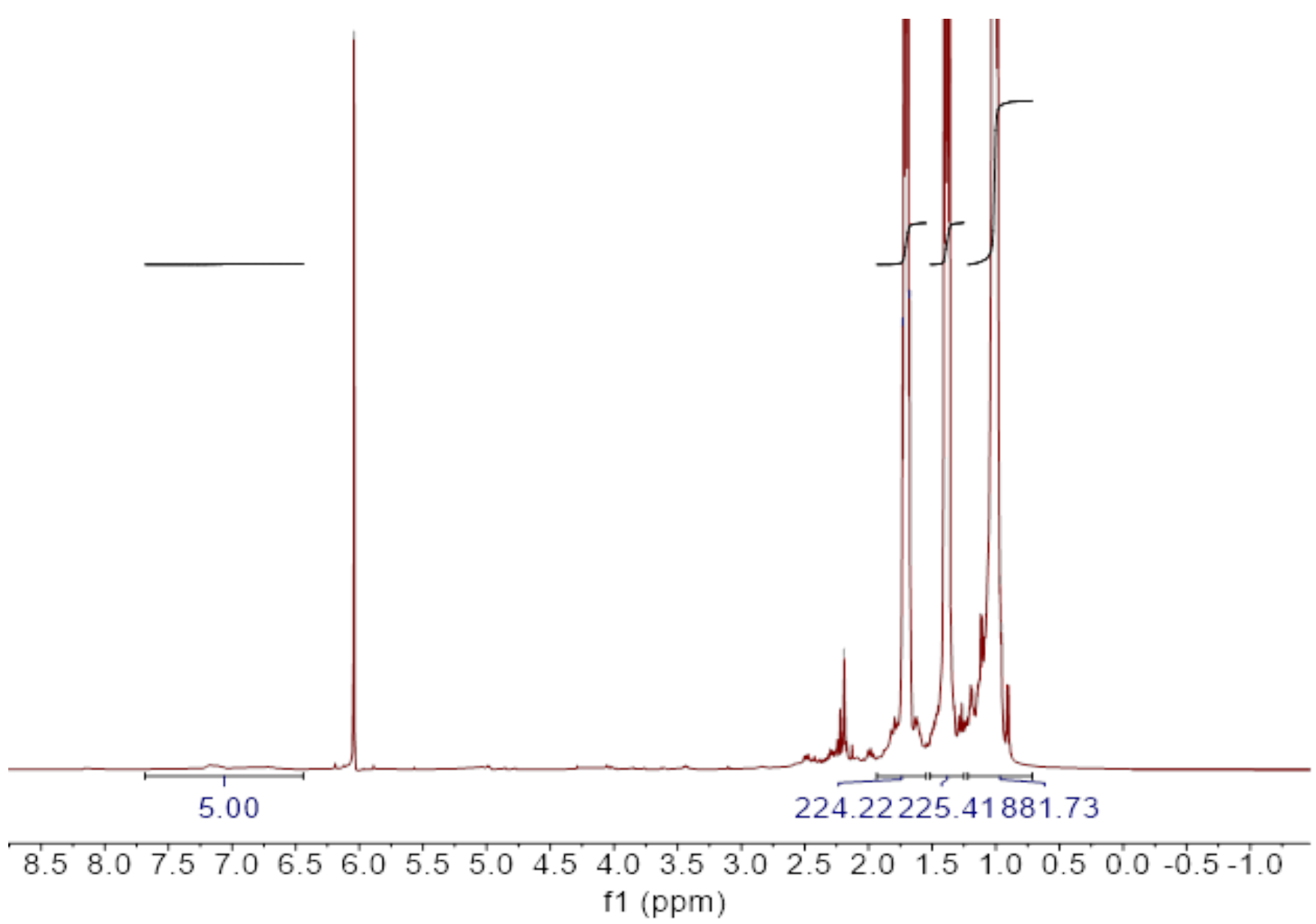

Figure S9. ^{1}H-NMR of PP-g-St modified with 0.5mL styrene/mL xylene, DCP=0.1g/mL.

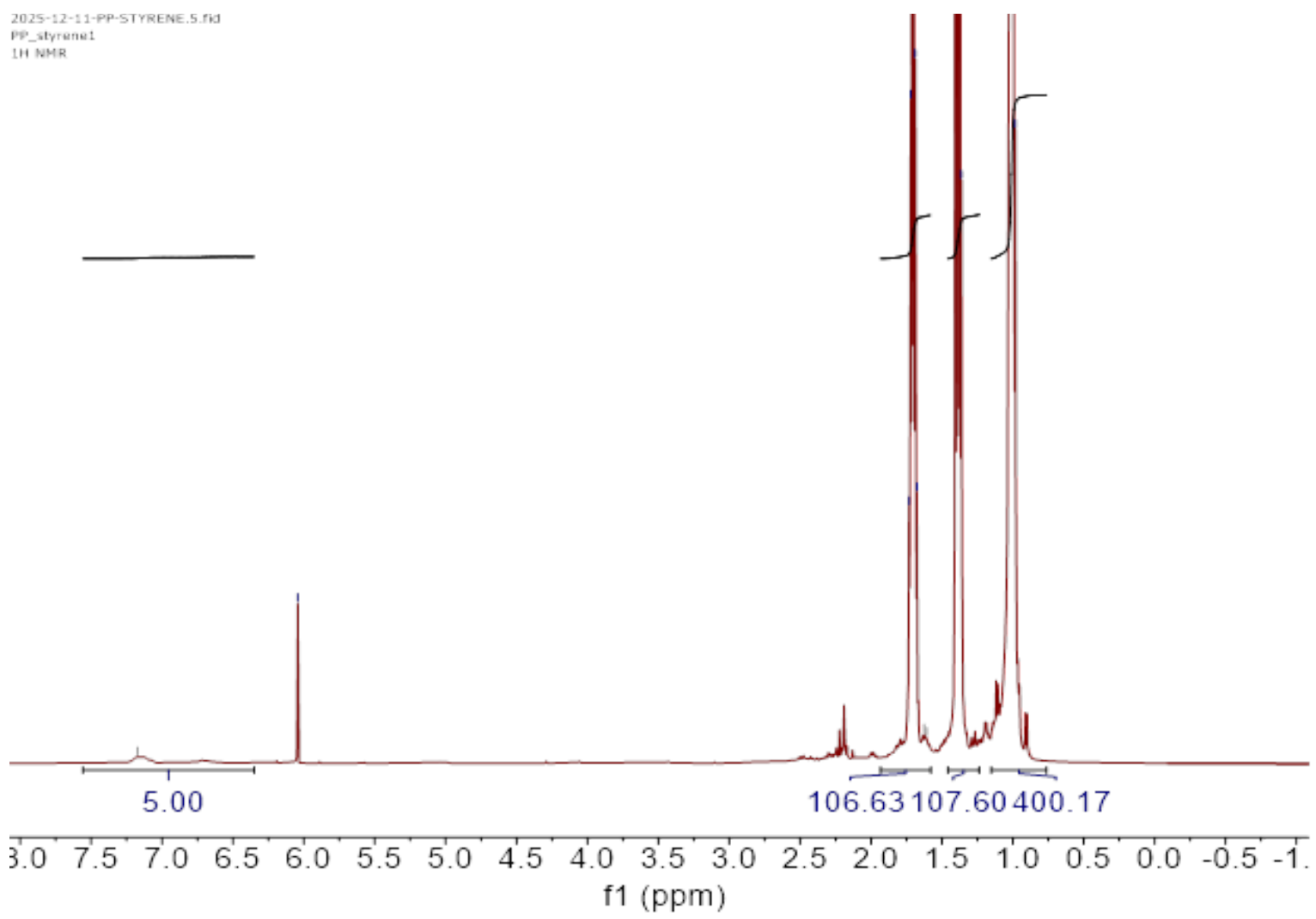

Figure S10. ^{1}H-NMR of PP-g-St modified with 1mL styrene/mL xylene, DCP=0.1g/mL.

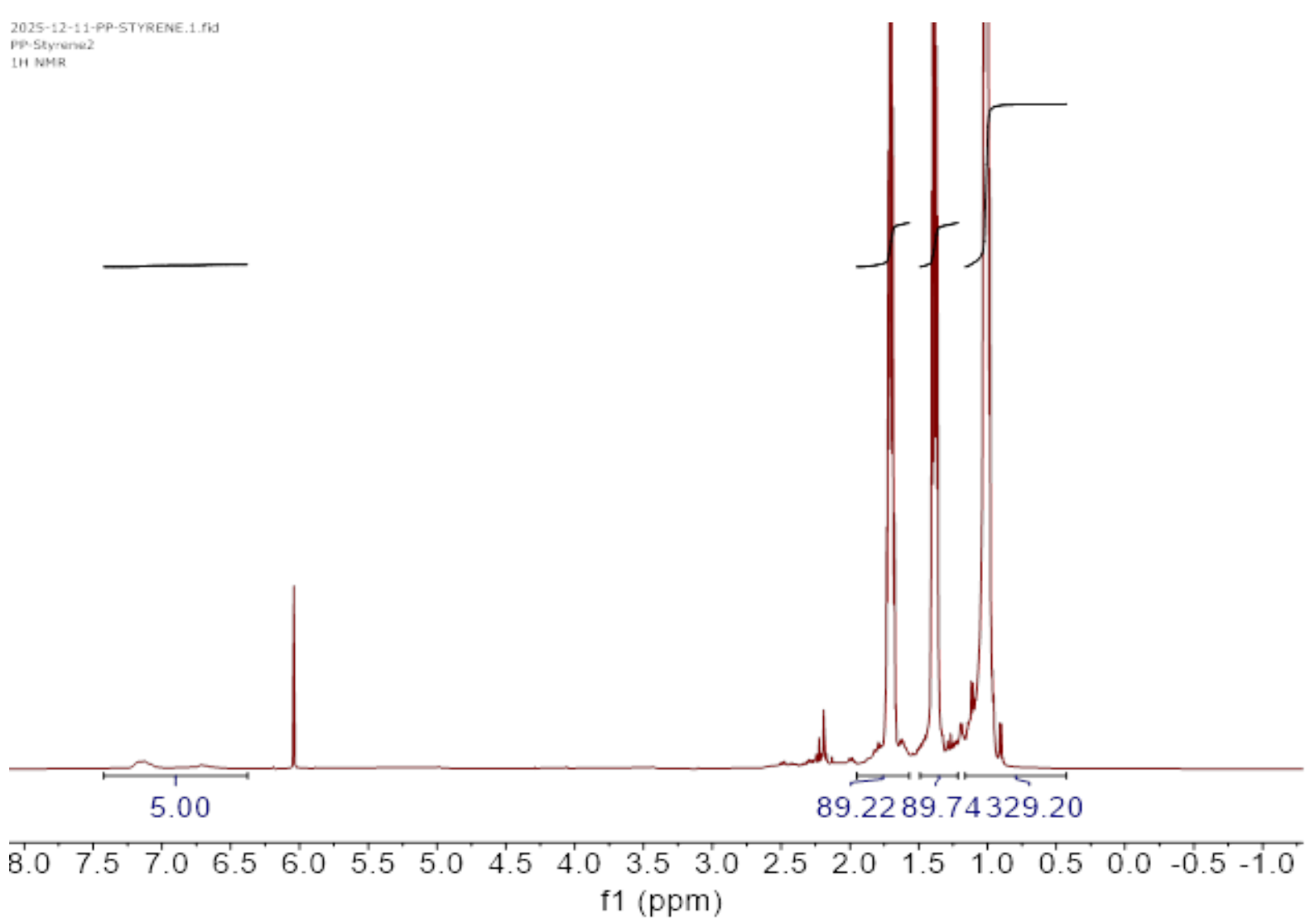

Figure S11. ^{1}H-NMR of PP-g-St modified with 2mL styrene/mL xylene, DCP=0.1g/mL.

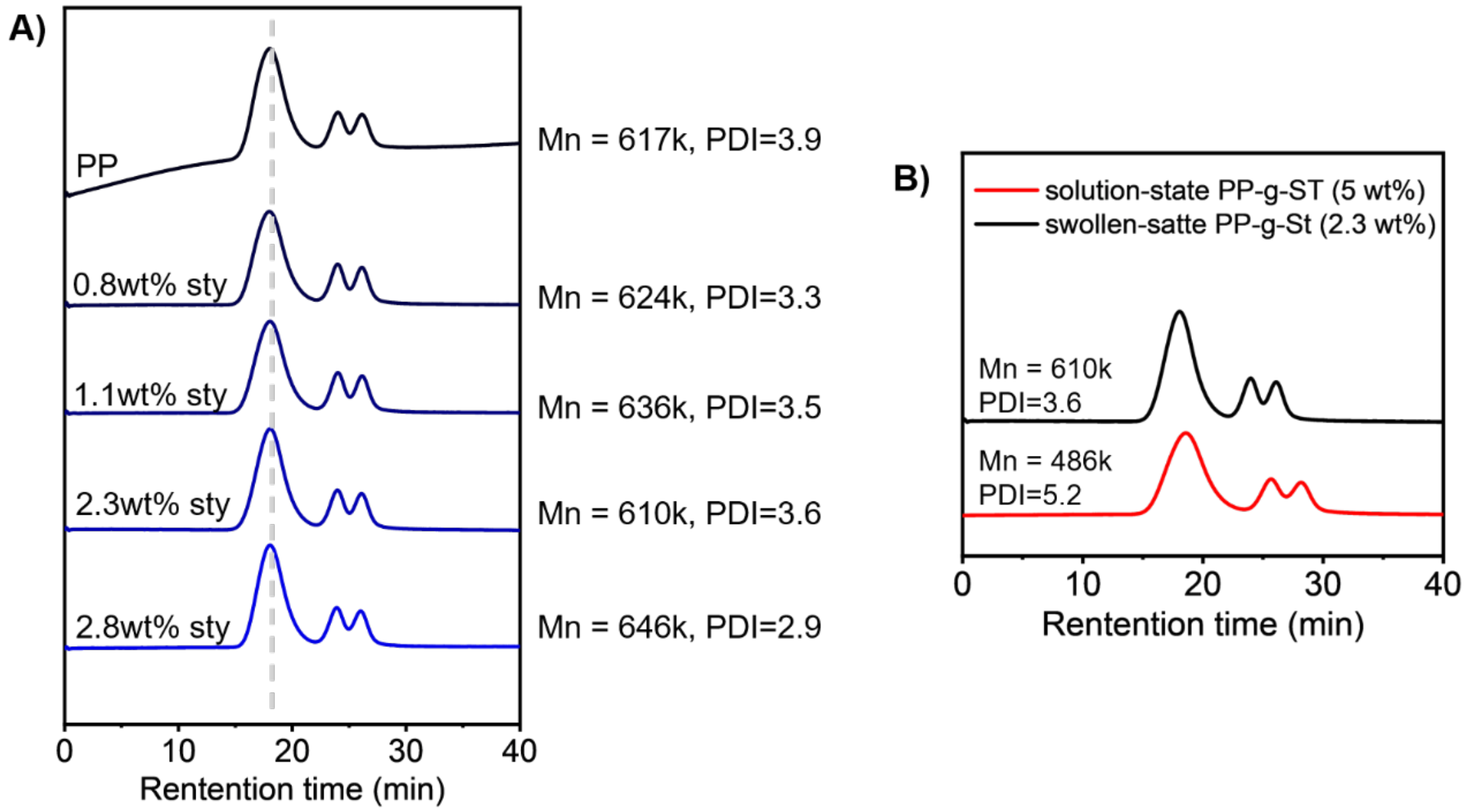

Figure S12. A) Gel permeation chromatography chromatogram of neat PP and PP-g-St with different styrene contents and B) gel permeation chromatography chromatogram of swollen-state functionalized PP (2.3 wt%, assuming swelling ratio = 10% by thickness/33% by volume and equal density of PP, xylene, and styrene, in a swollen PP, the mass ratio of PP:DCP = 1:0.03) and solution-state functionalized PP (5 wt%, mass ratio of PP: DCP=1:0.35).

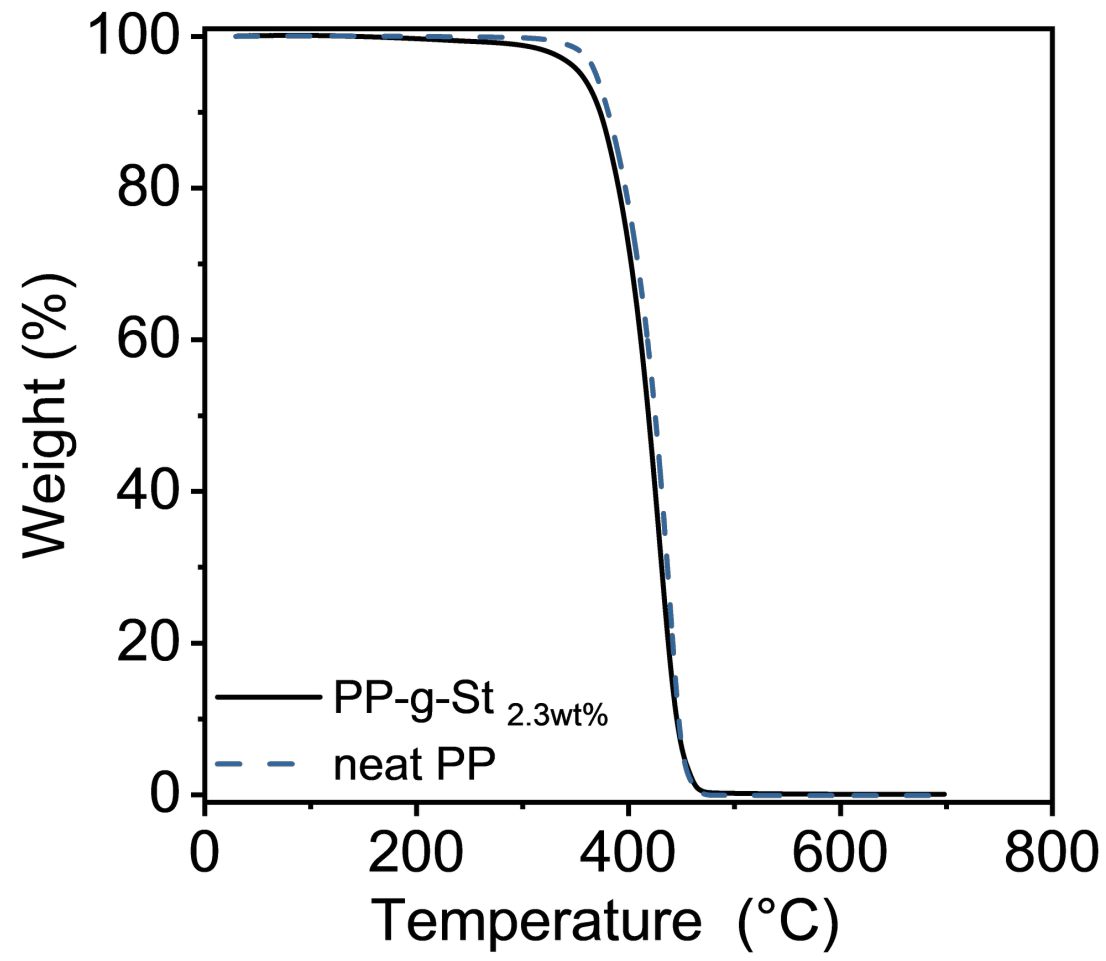

Figure S13. TGA of neat PP and PP-g-St (styrene content = 2.3 wt%).

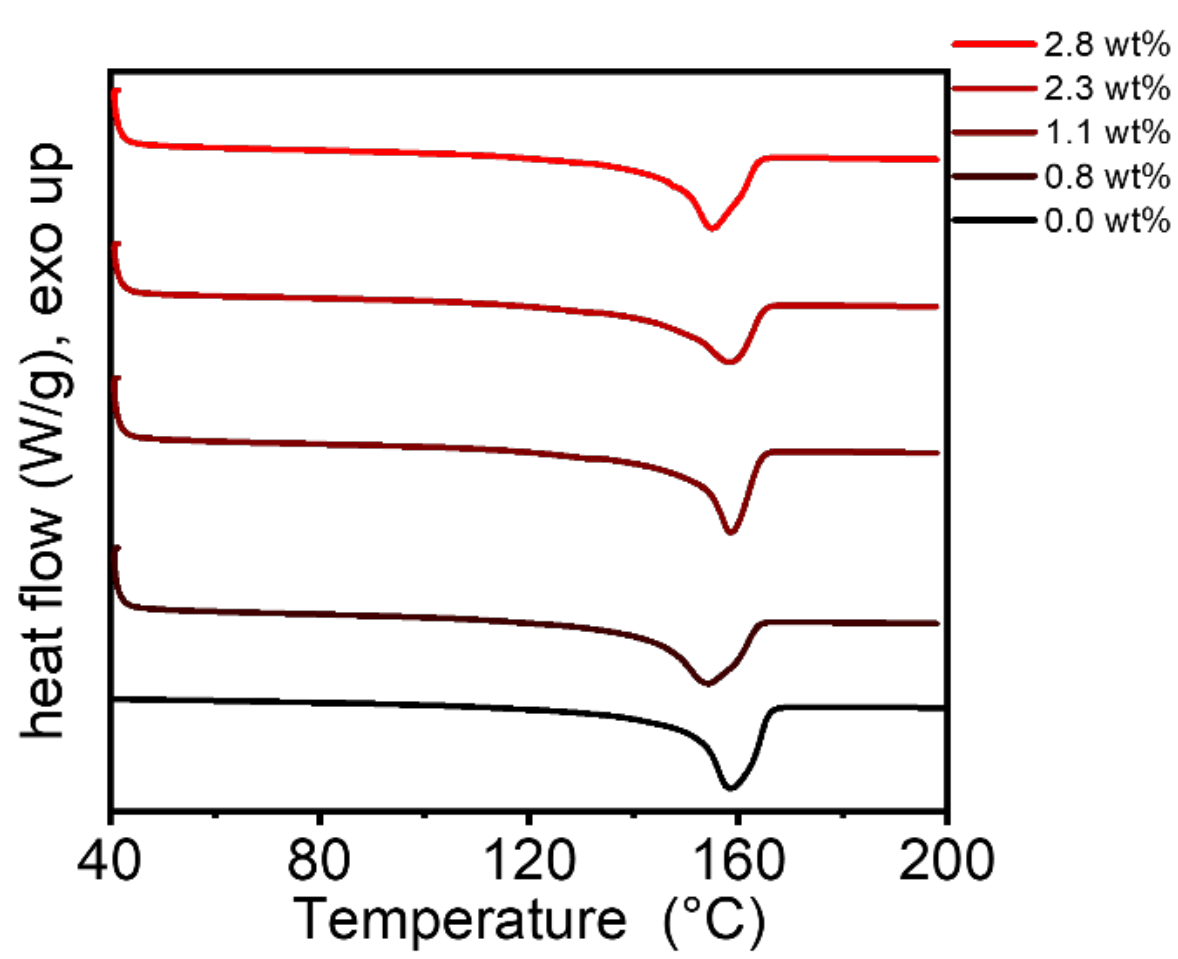

Figure S14. DSC thermograms of the second heating cycle at a scan rate of 10 °C/min for PP-g-St with different styrene content.

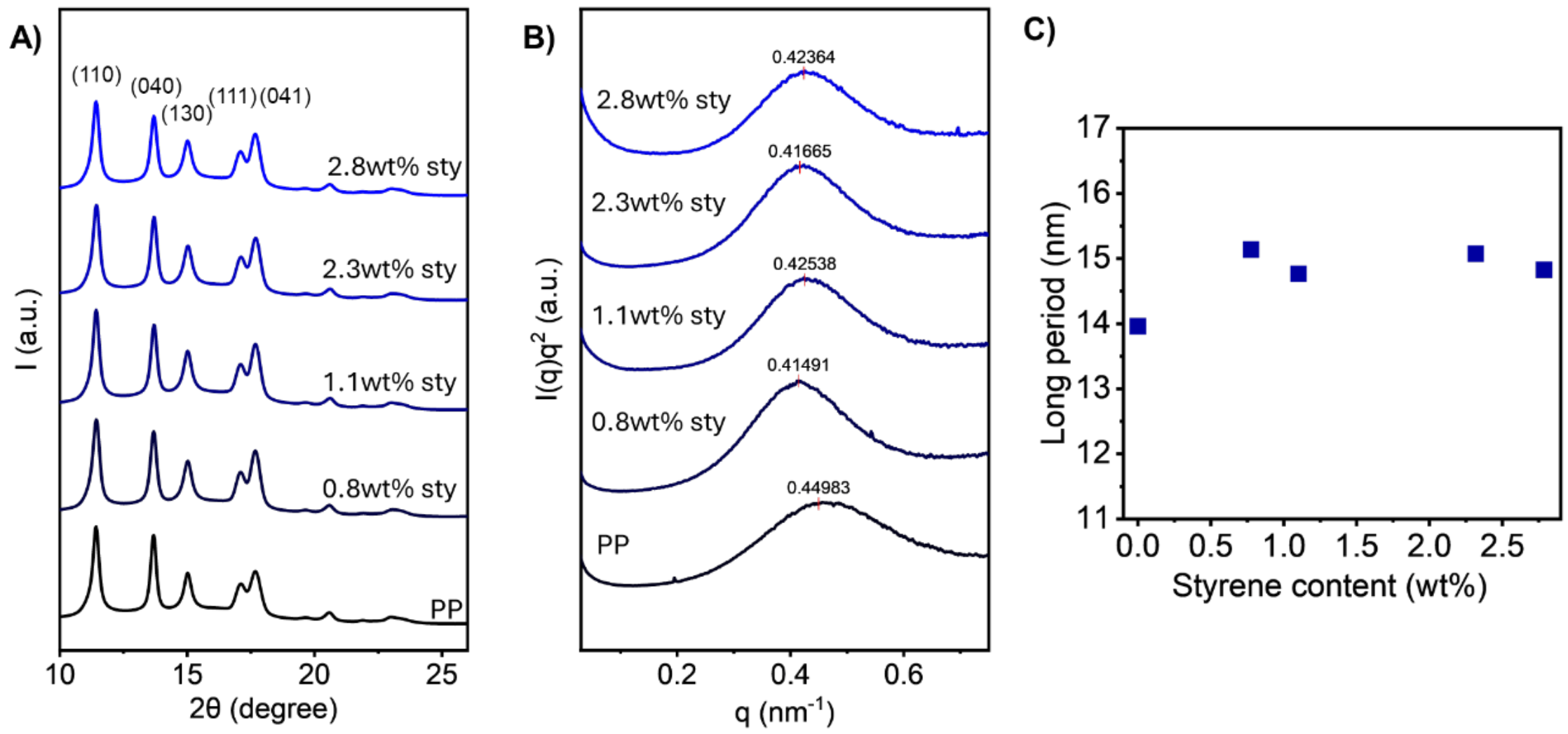

Figure S15. A) WAXS plots, B) Lorentz-corrected SAXS plots, and C) long period of neat PP and PP-g-St with different styrene content.

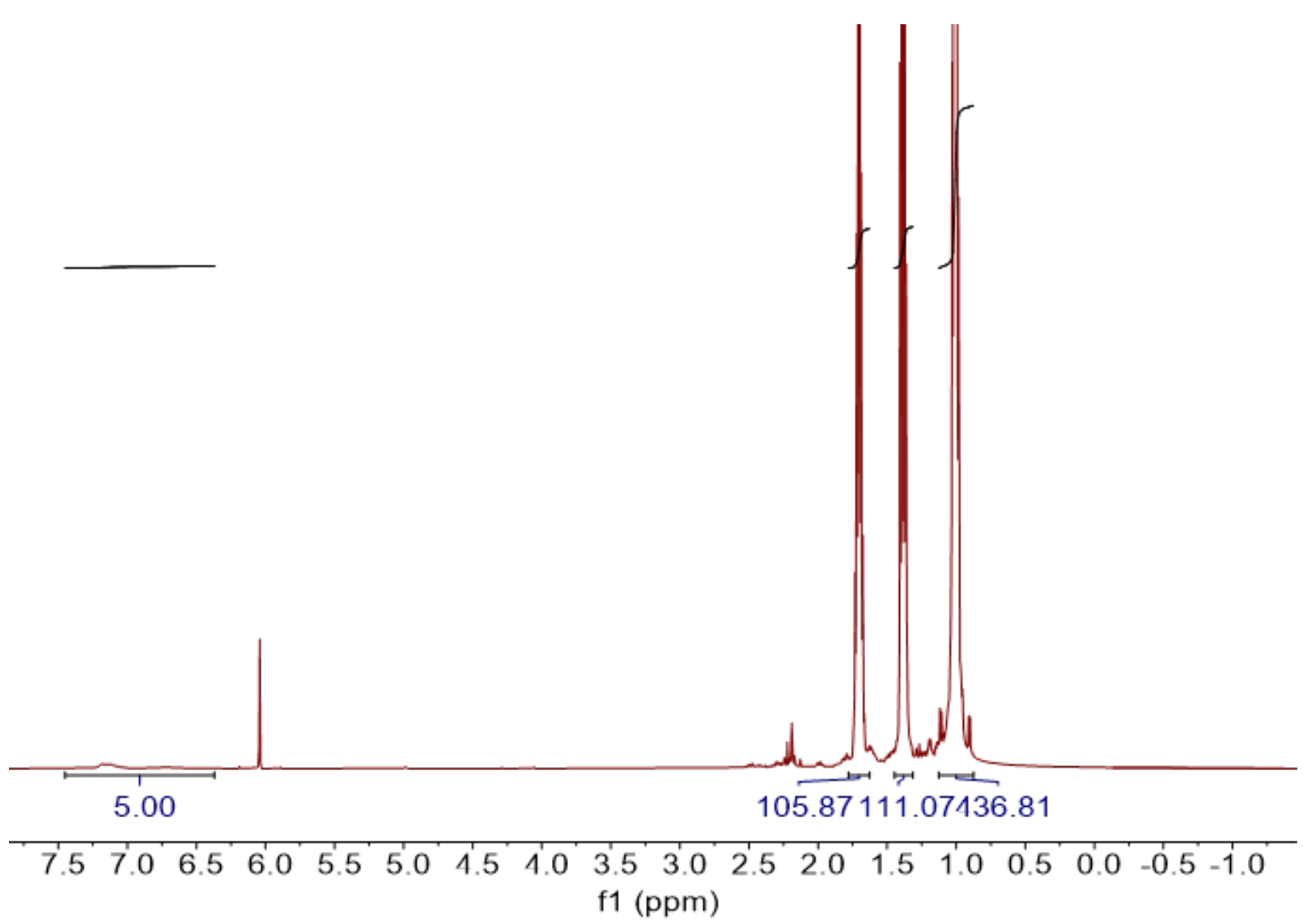

Figure S16. ^{1}H-NMR of PP-g-St obtained from solution state functionalization (2 g of PP, 100 mL of xylene, 0.06g of DCP, and 0.06 g of styrene, at 130 °C for 24h).

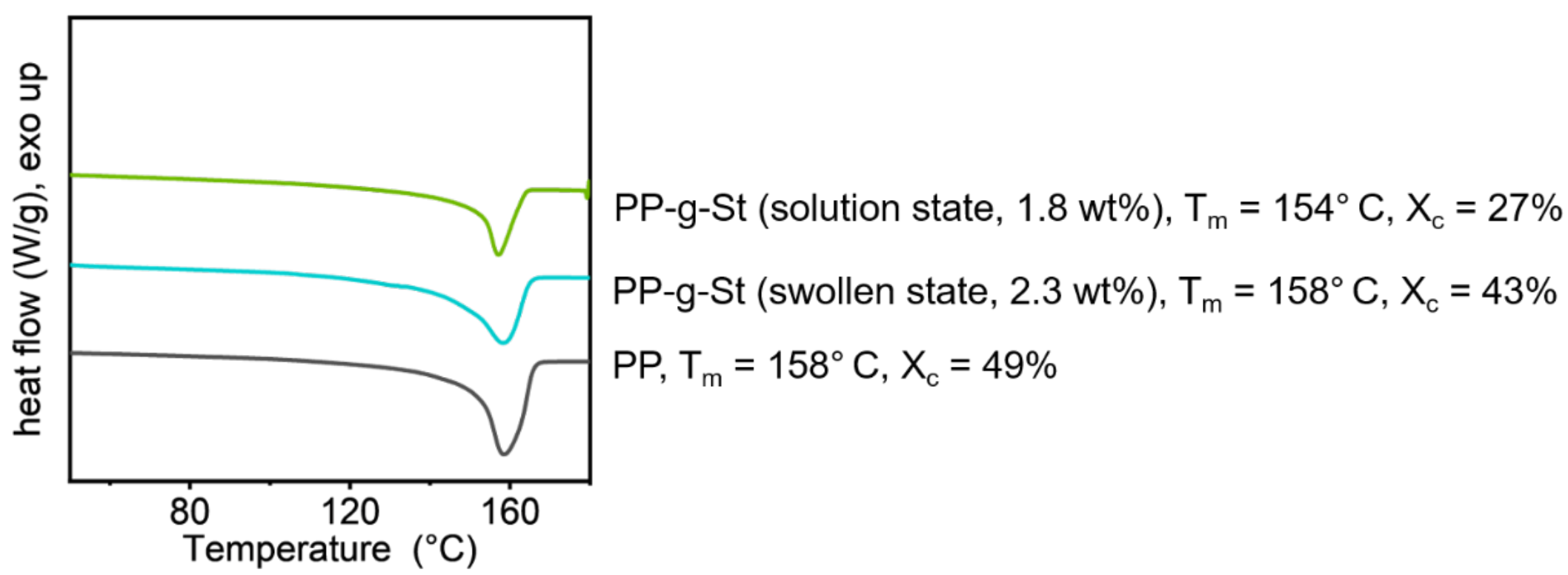

Figure S17. DSC thermograms of the second heating cycle at a scan rate of 10 °C/min for neat PP, PP-g-St (swollen state, styrene content = 2.3wt%), and PP-g-St (solution state , styrene content = 1.8 wt%).

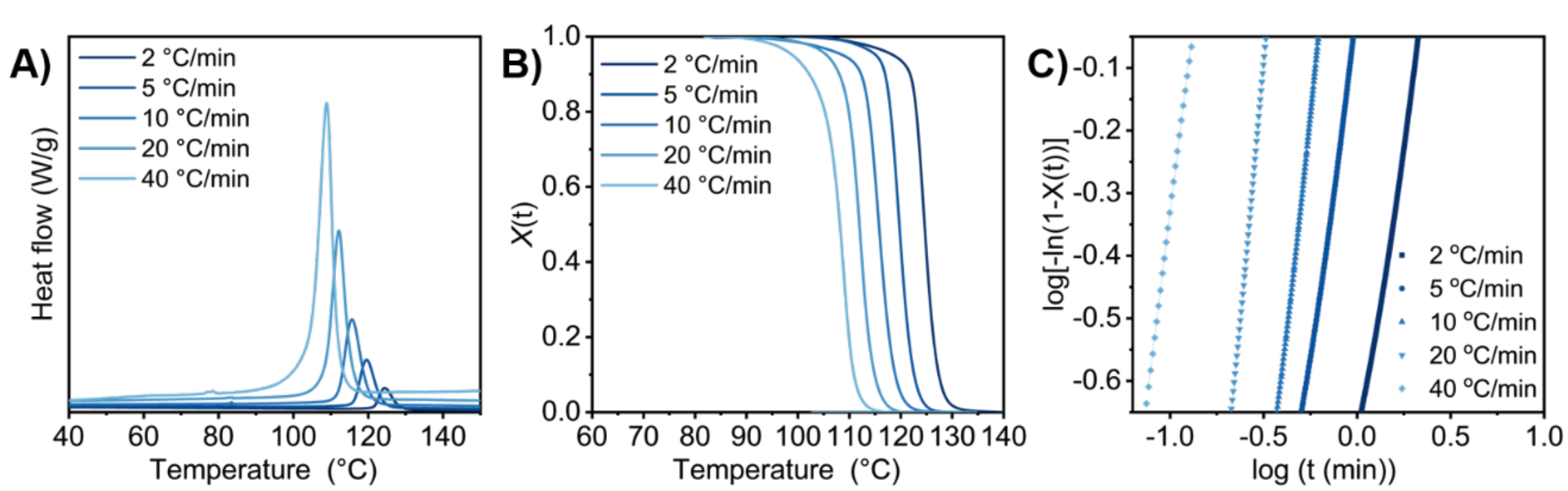

Figure S18. (A) Representative non-isothermal crystallization exotherms at different cooling rates, (B) relative crystallinity as a function of temperature for the non-isothermal crystallization, and (C) linear fit of the Avrami plot in the range of 30% ≤ X(t) ≤ 60% for neat PP.

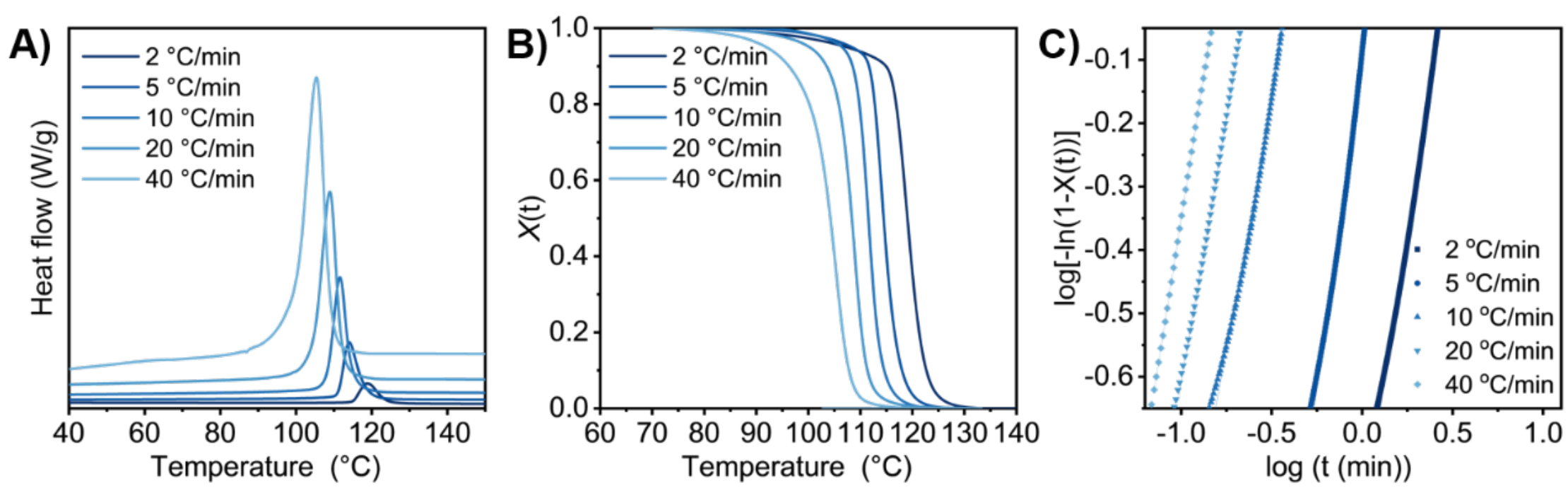

Figure S19. (A) Representative non-isothermal crystallization exotherms at different cooling rates, (B) relative crystallinity as a function of temperature for the non-isothermal crystallization, and (C) linear fit of the Avrami plot in the range of 30% ≤ X(t) ≤ 60% for PP-g-St of 1.1 wt% styrene content from swollen state modification.

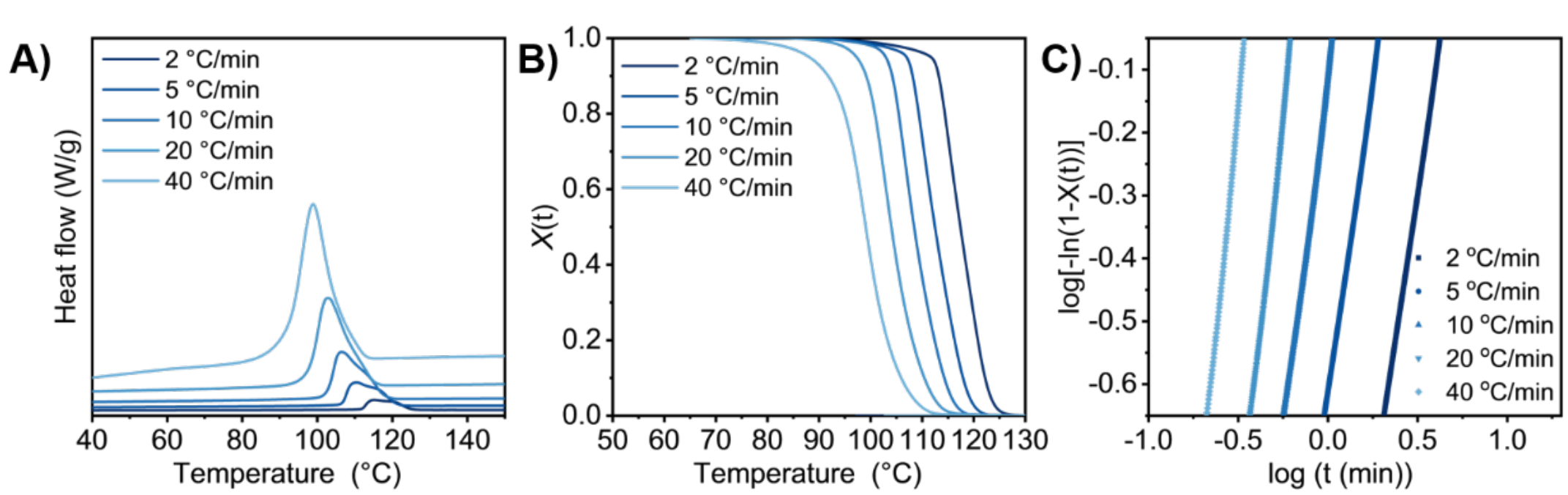

Figure S20. (A) Representative non-isothermal crystallization exotherms at different cooling rates, (B) relative crystallinity as a function of temperature for the non-isothermal crystallization, and (C) linear fit of the Avrami plot in the range of 30% ≤ X(t) ≤ 60% for PP-g-St of 2.8 wt% styrene content from swollen state modification.

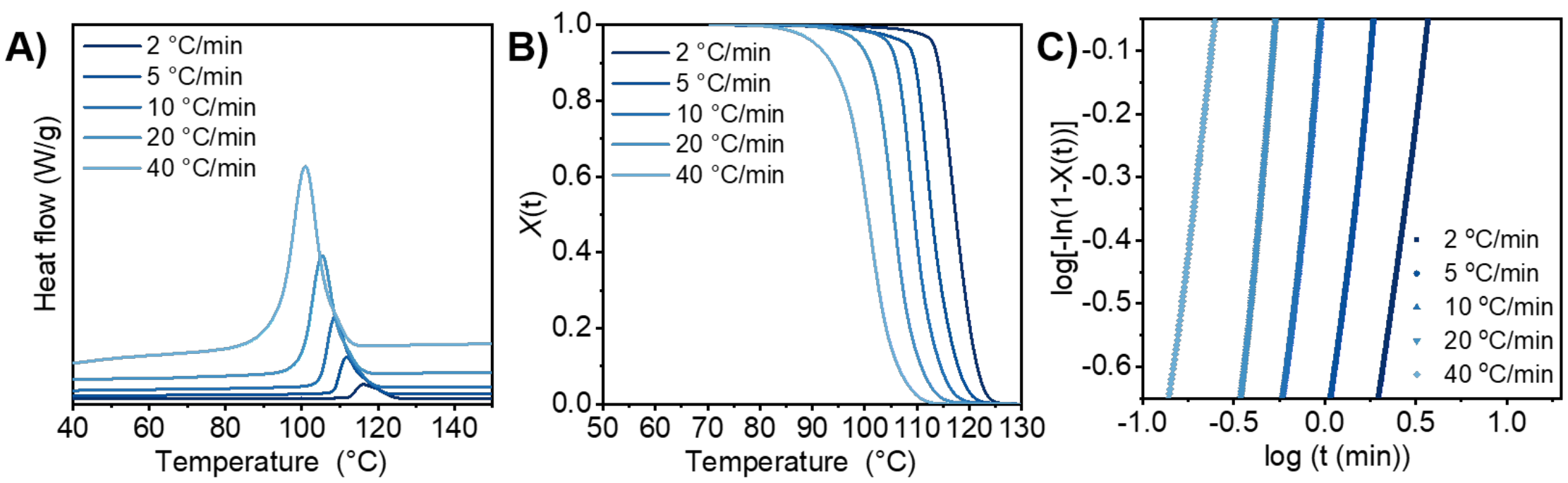

Figure S21. (A) Representative non-isothermal crystallization exotherms at different cooling rates, (B) relative crystallinity as a function of temperature for the non-isothermal crystallization, and (C) linear fit of the Avrami plot in the range of 30% ≤ X(t) ≤ 60% for PP-g-St of PP-g-St of 1.8 wt% styrene content from solution state modification.

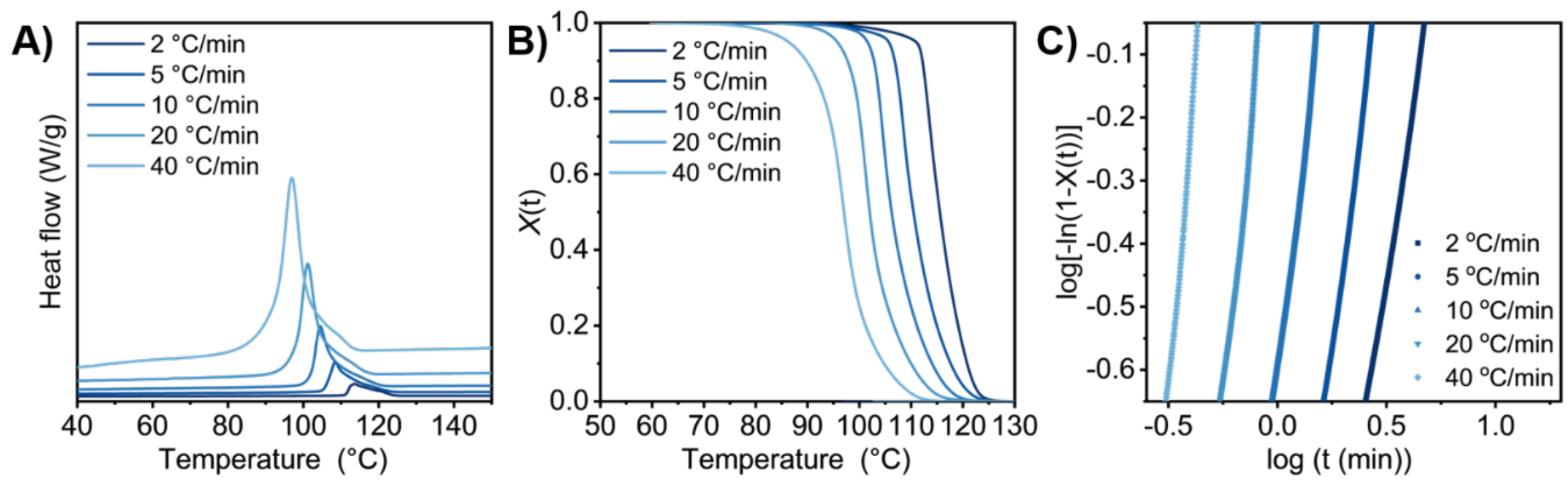

Figure S22. (A) Representative non-isothermal crystallization exotherms at different cooling rates, (B) relative crystallinity as a function of temperature for the non-isothermal crystallization, and (C) linear fit of the Avrami plot in the range of 30% ≤ X(t) ≤ 60% for PP-g-St of PP-g-St of 5.0 wt% styrene content from solution state modification.

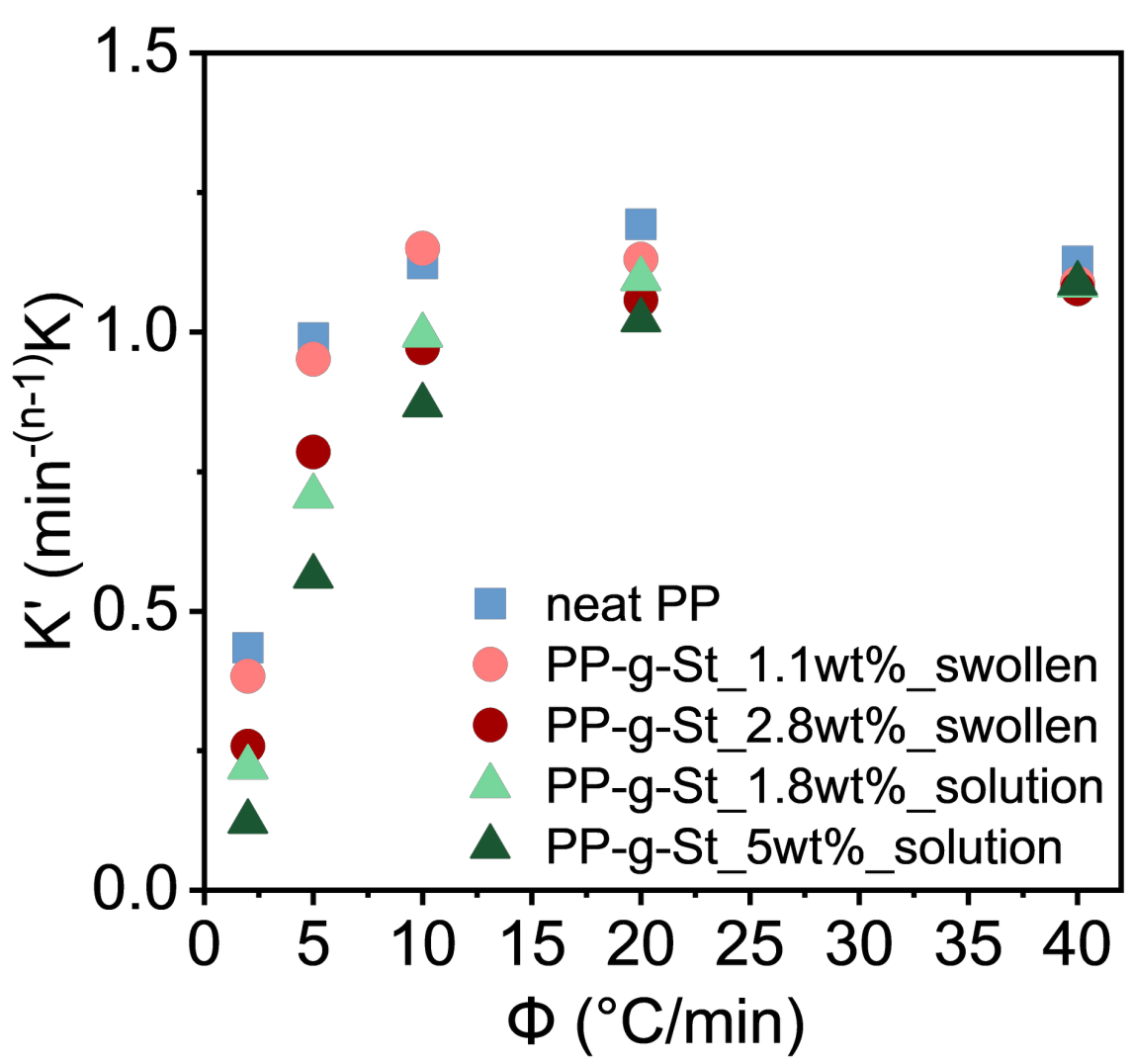

Figure S23. Jeziorny-modified Avrami constant K' as a function of cooling rate (ϕ) for neat PP, PP-g-St of 1.1 wt% and 2.8 wt% styrene contents from swollen state modification and PP-g-St of 1.8 wt% and 5.0 wt% styrene contents from solution state modification.

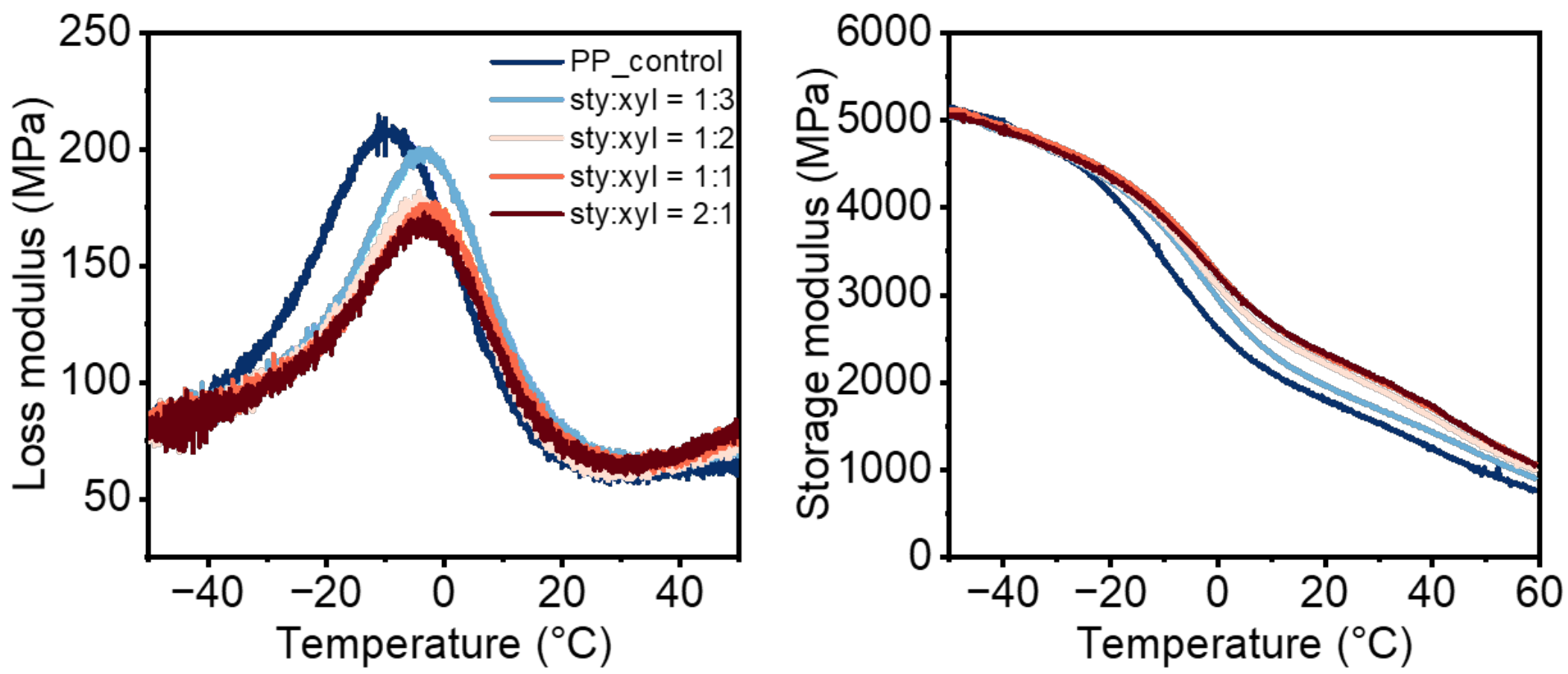

Figure S24. Loss modulus and storage modulus obtained from DMA.

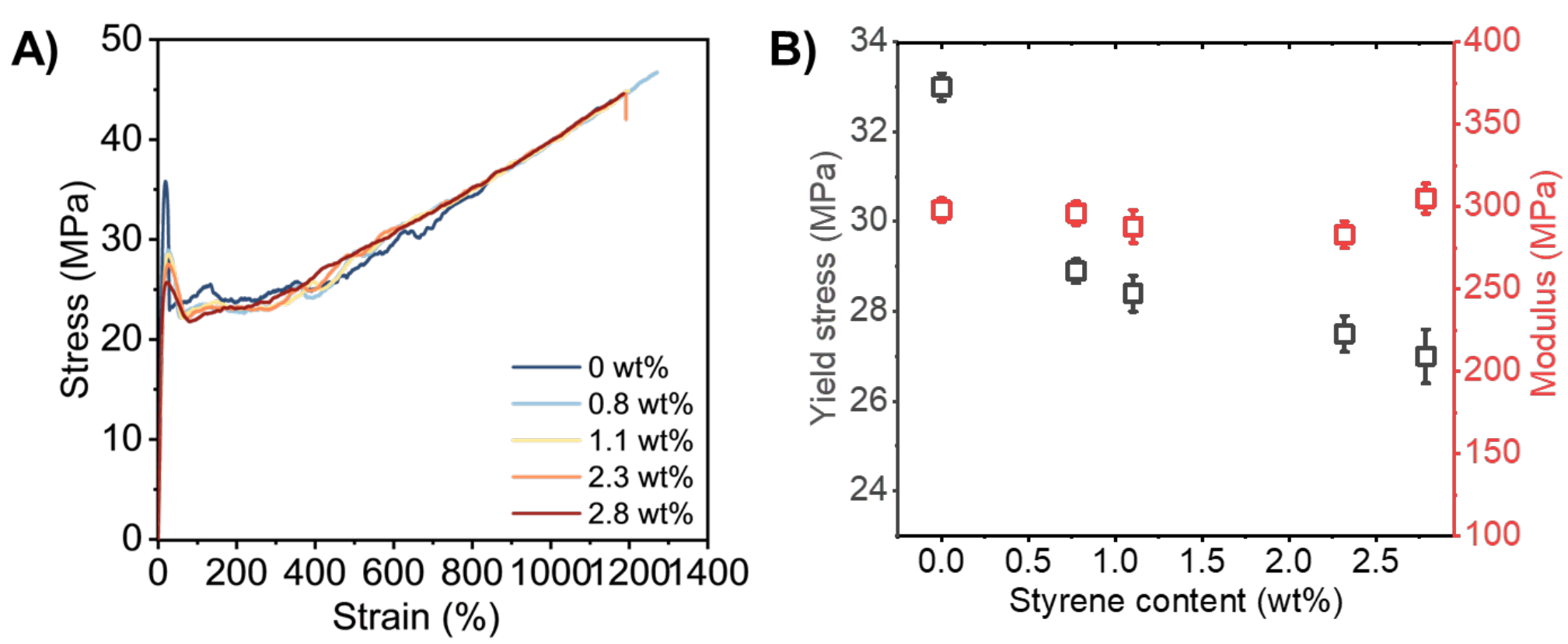

Figure S25. A) Stress-strain curves, B) Young's modulus and upper yield stress of PP-g-St of different styrene content.

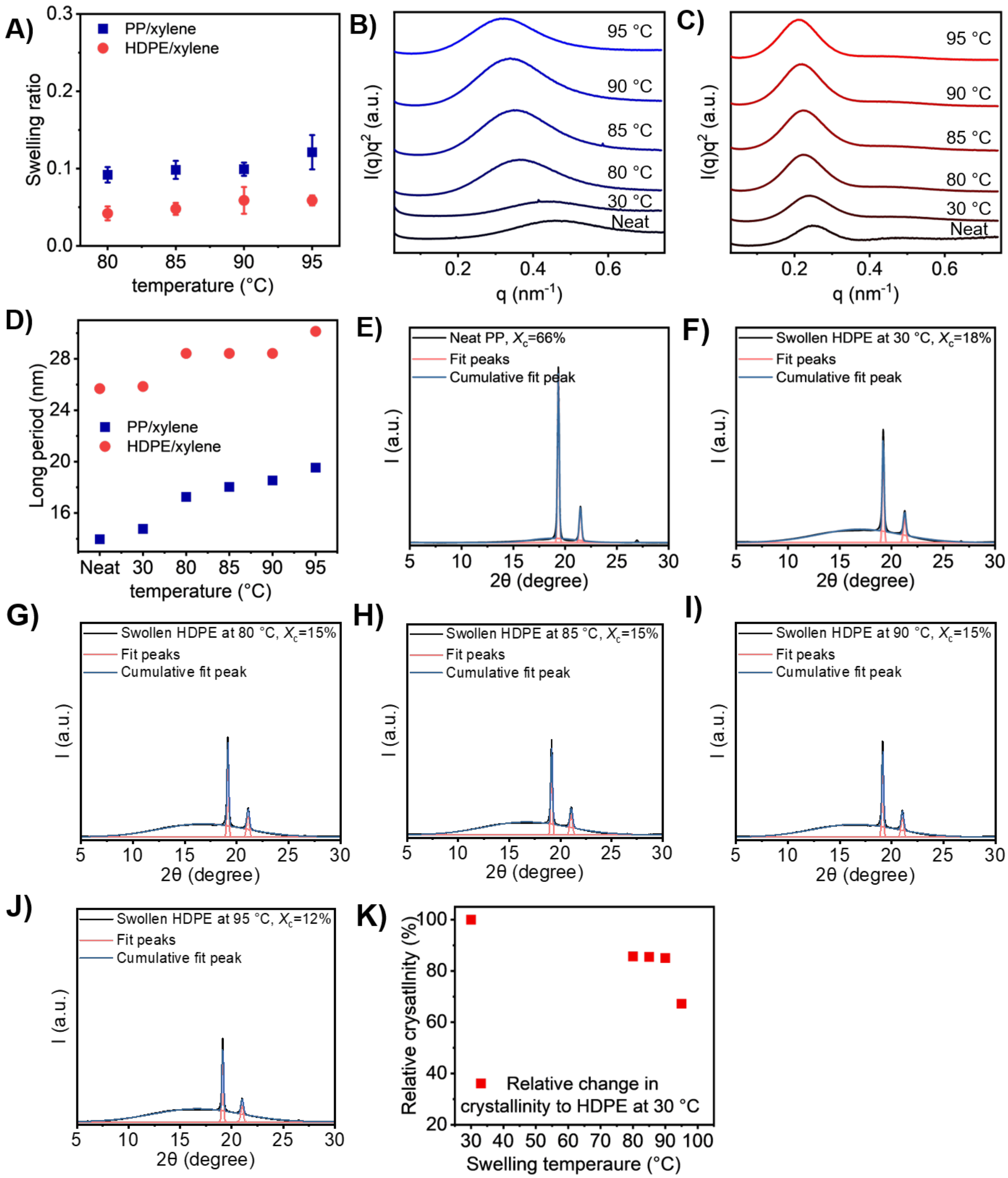

Figure S26. A) Swelling ratio of PP and HDPE in xylene as a function of temperature, and Lorentz corrected SAXS plots of B) PP, C) HDPE in xylene at different swollen temperatures, D) long period of neat and swollen PP and HDPE at different temperatures, deconvolution of

the crystalline reflexes and the amorphous halo (Gaussian fit) for E) neat HDPE; F) to J) swollen HDPE at 30 °C to 95 °C; and I) relative change in crystallinity compared to the swollen HDPE at 30 °C.

.

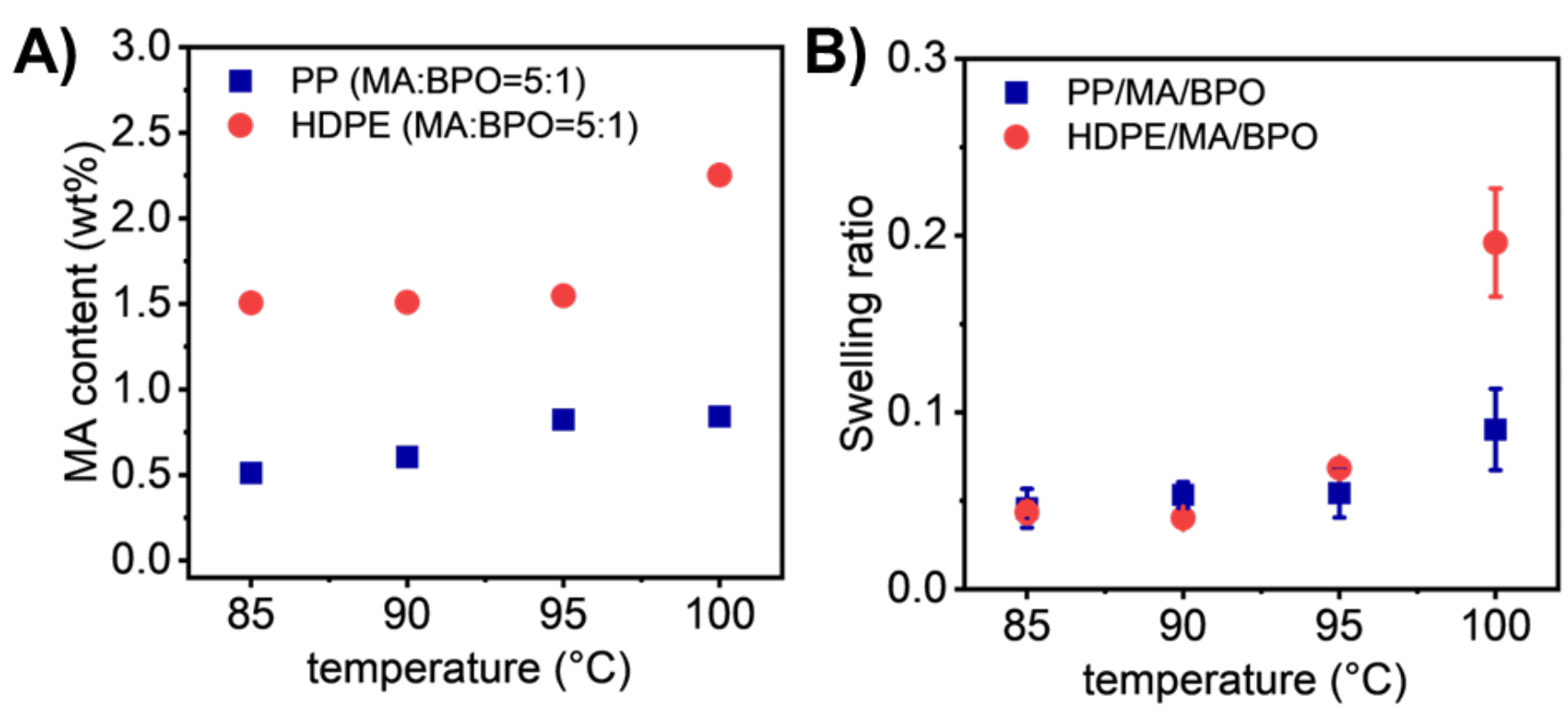

Figure S27. A) MA content and B) swelling ratio as a function of temperature for MA functionalization of PP and HDPE with 0.2g/mL BPO and 1g/ mL of MA for 24h.

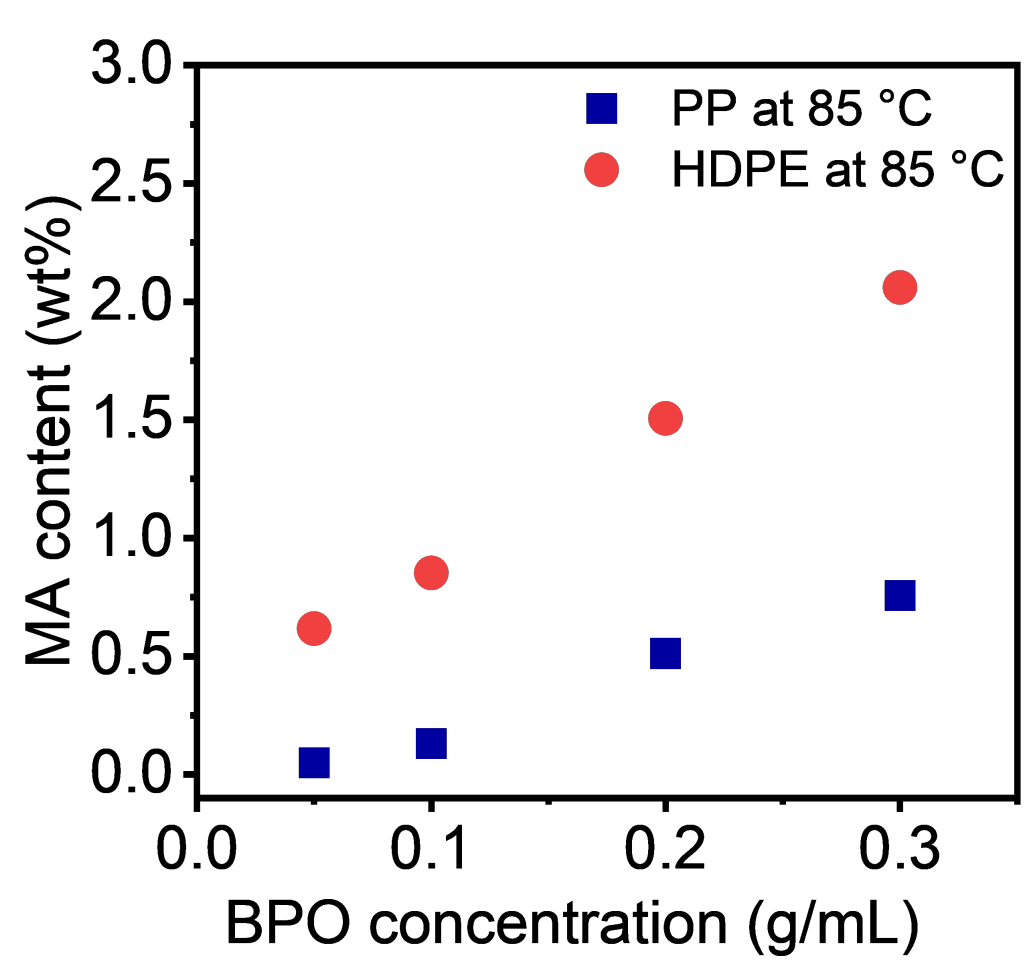

Figure S28 MA content of PP-g-MA and HDPE-g-MA as a function of BPO concentration.

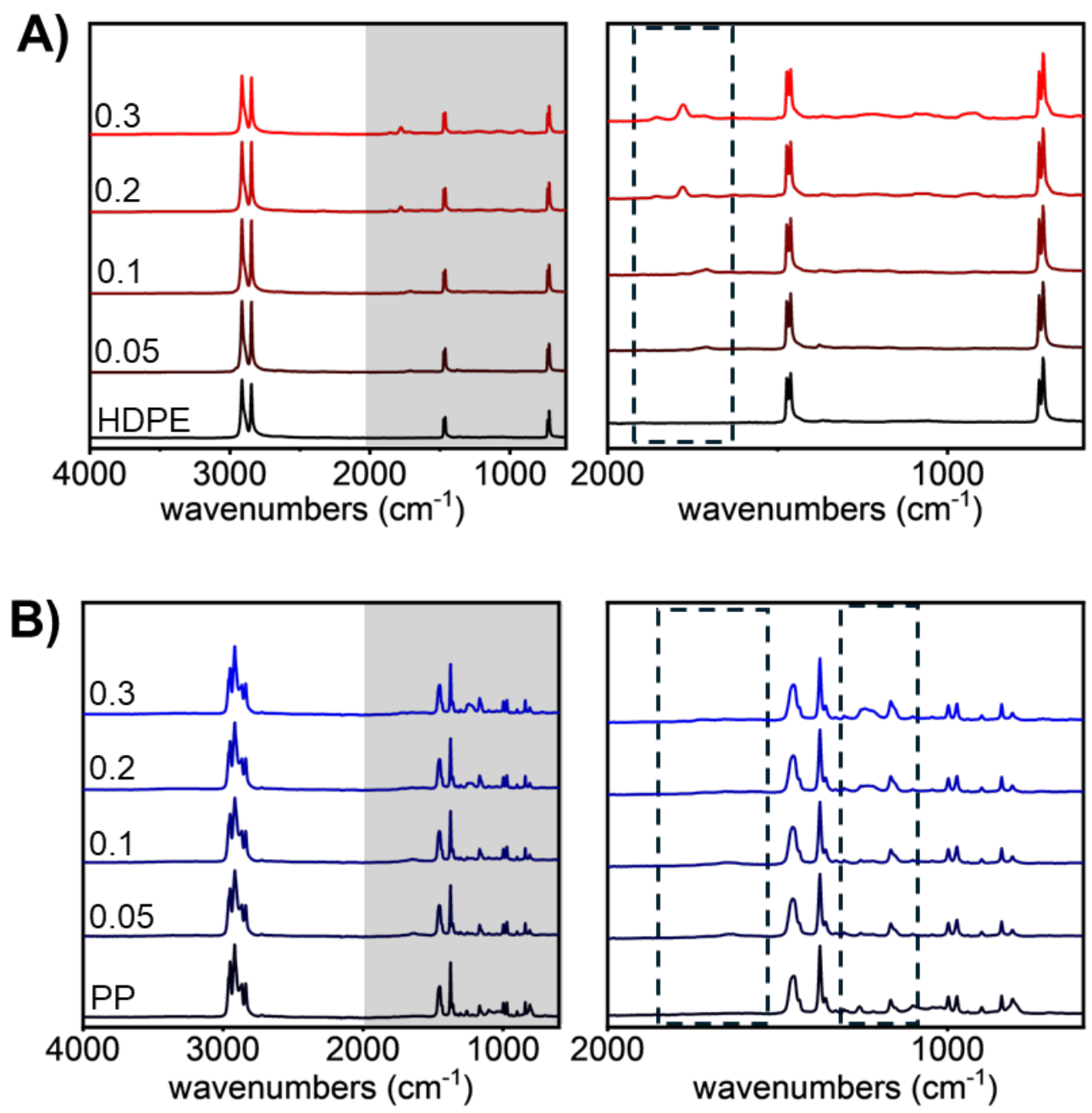

Figure S29. FT-IR of MA functionalized A) HDPE and B) PP at BPO concentration from 0.05 g/mL to 0.3 g/mL (labeled on the left of figures).

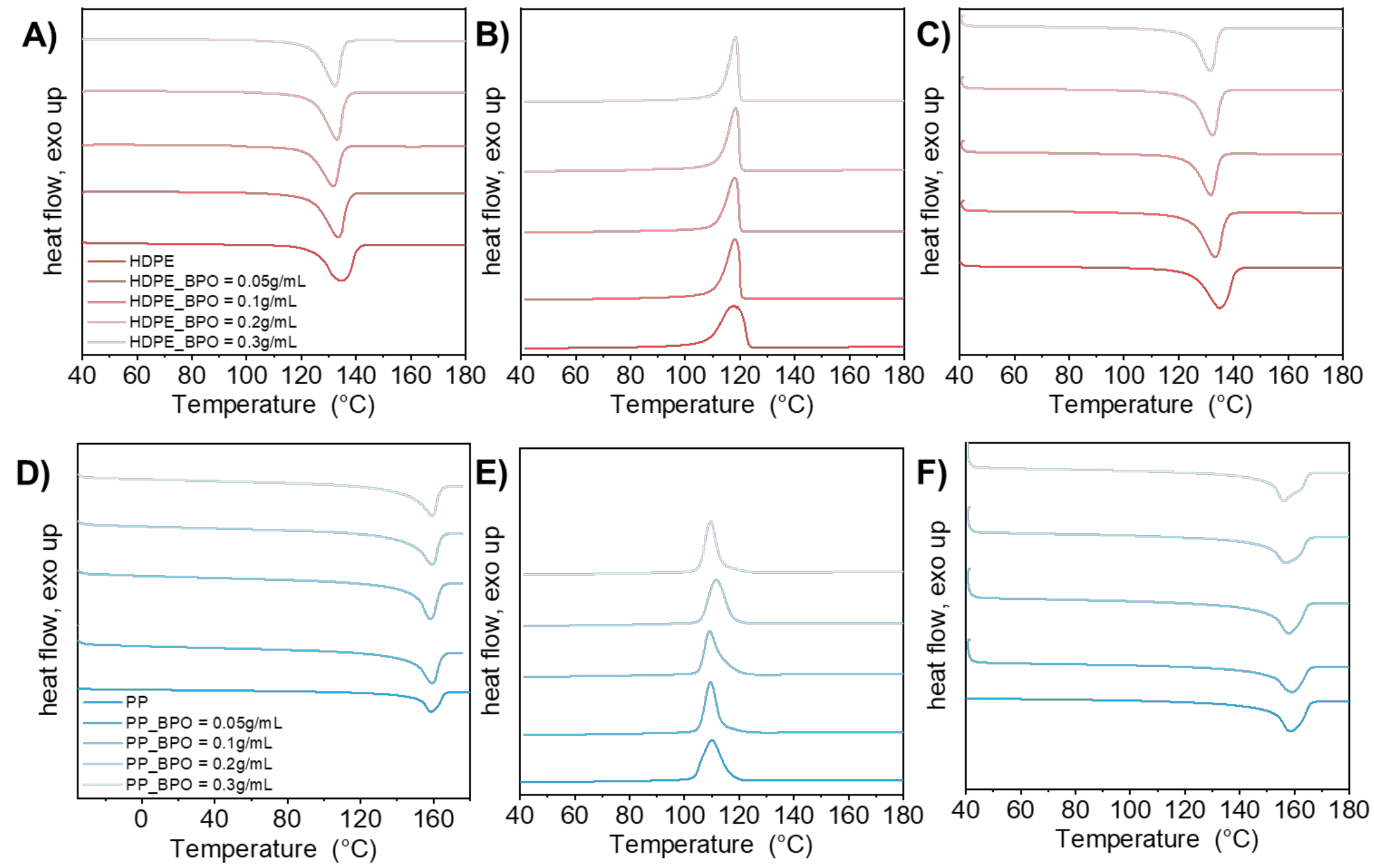

Figure S30. DSC thermograms of A) first heating, B) cooling, and C) second heating of MA functionalization of HDPE as a function of BPO concentrations; D) first heating, E) cooling, and F) second heating of MA functionalization of PP as a function of BPO concentrations.

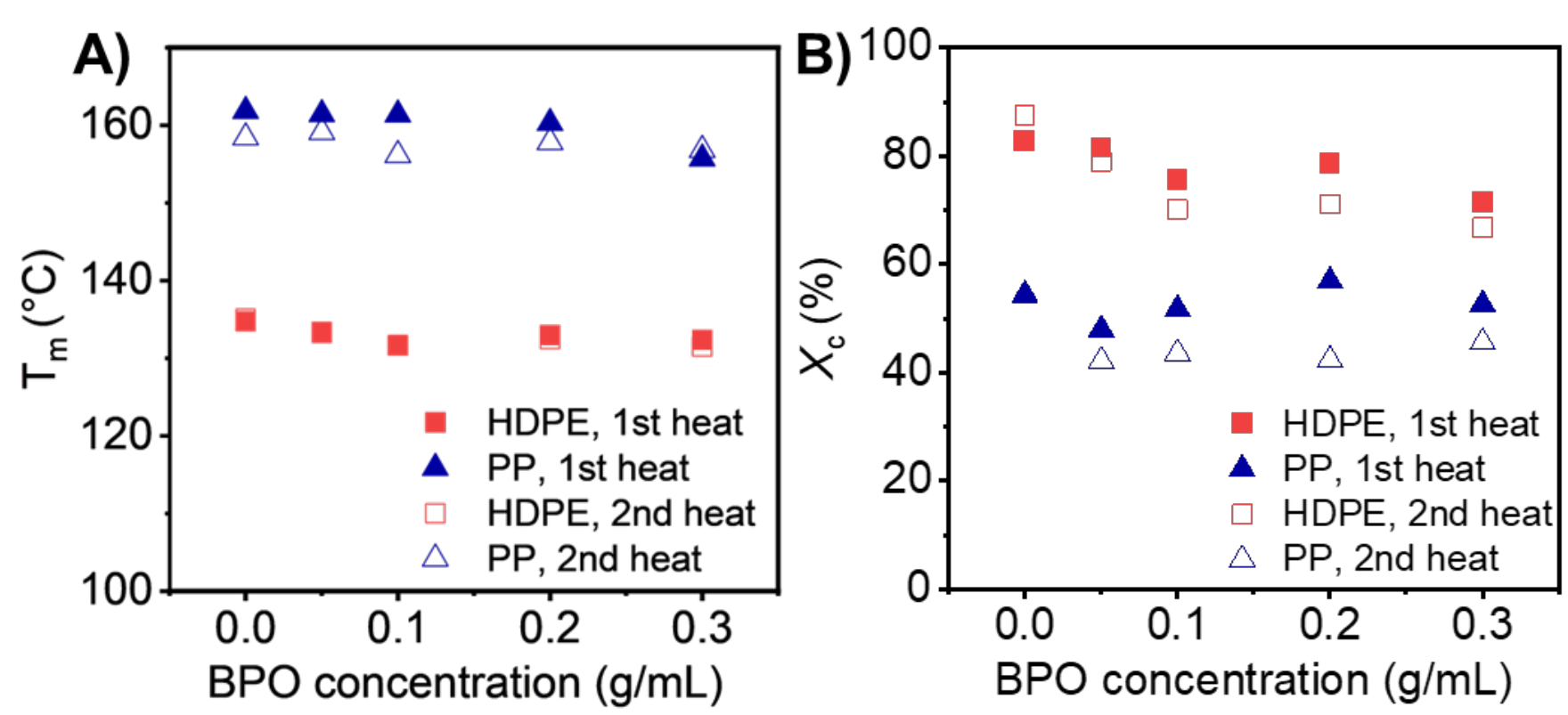

Figure S31. A) T_m and B) crystallinity of the MA functionalization of PP and HDPE as a function of BPO concentrations.

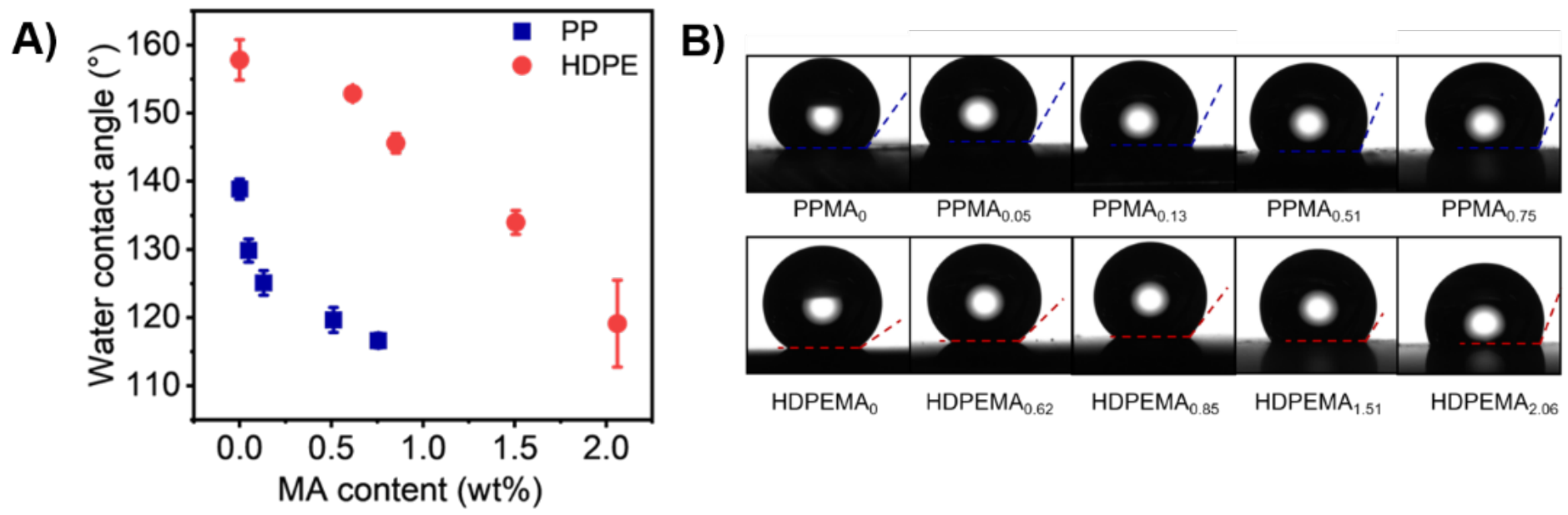

Figure S32. A) water contact angle of PP-g-MA and HDPE-g-MA as a function of MA content, and B) representative images of water contact angle measurements.

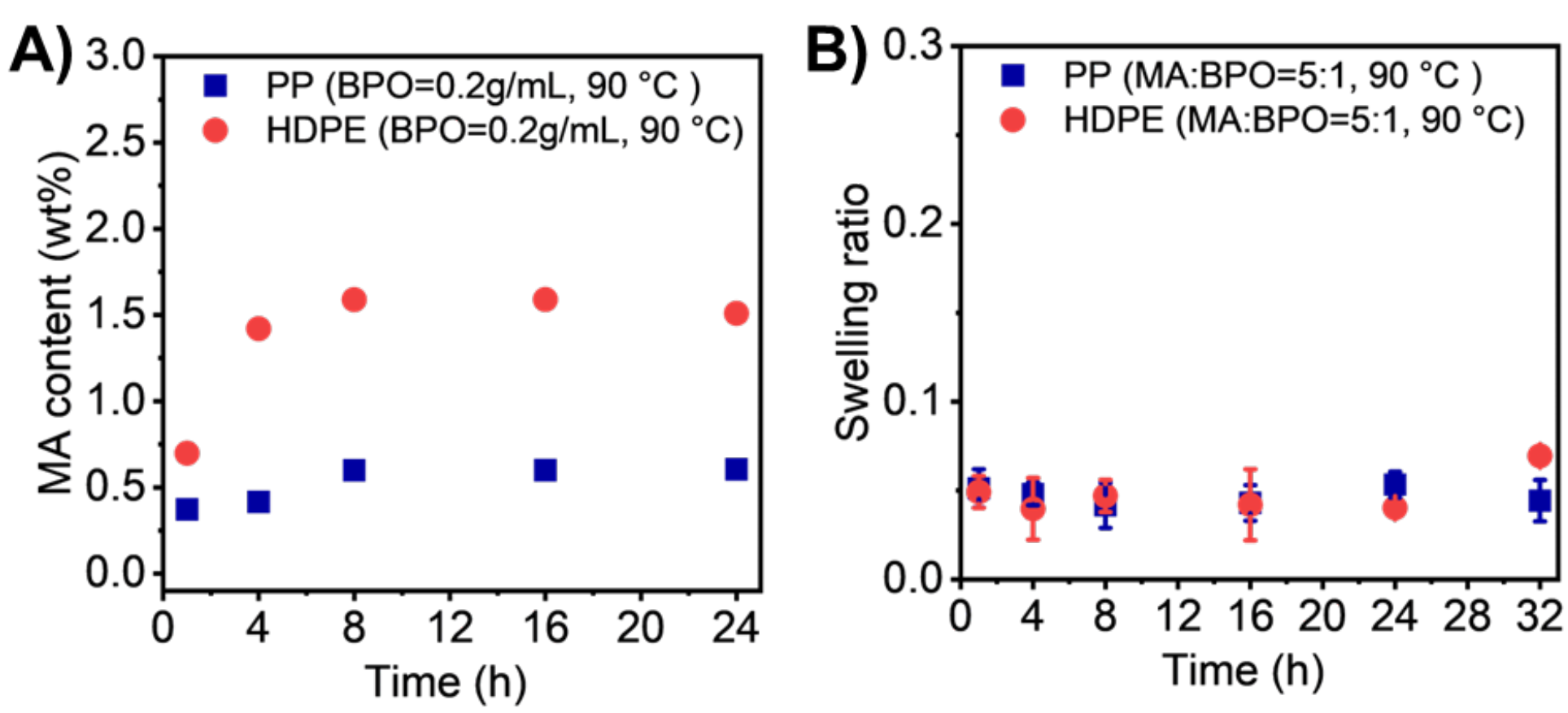

Figure S33. A) MA content and B) swelling ratio of the kinetic study for MA functionalization of PP and HDPE with 0.2g/mL of BPO and 1g/ mL of MA at 90 °C.

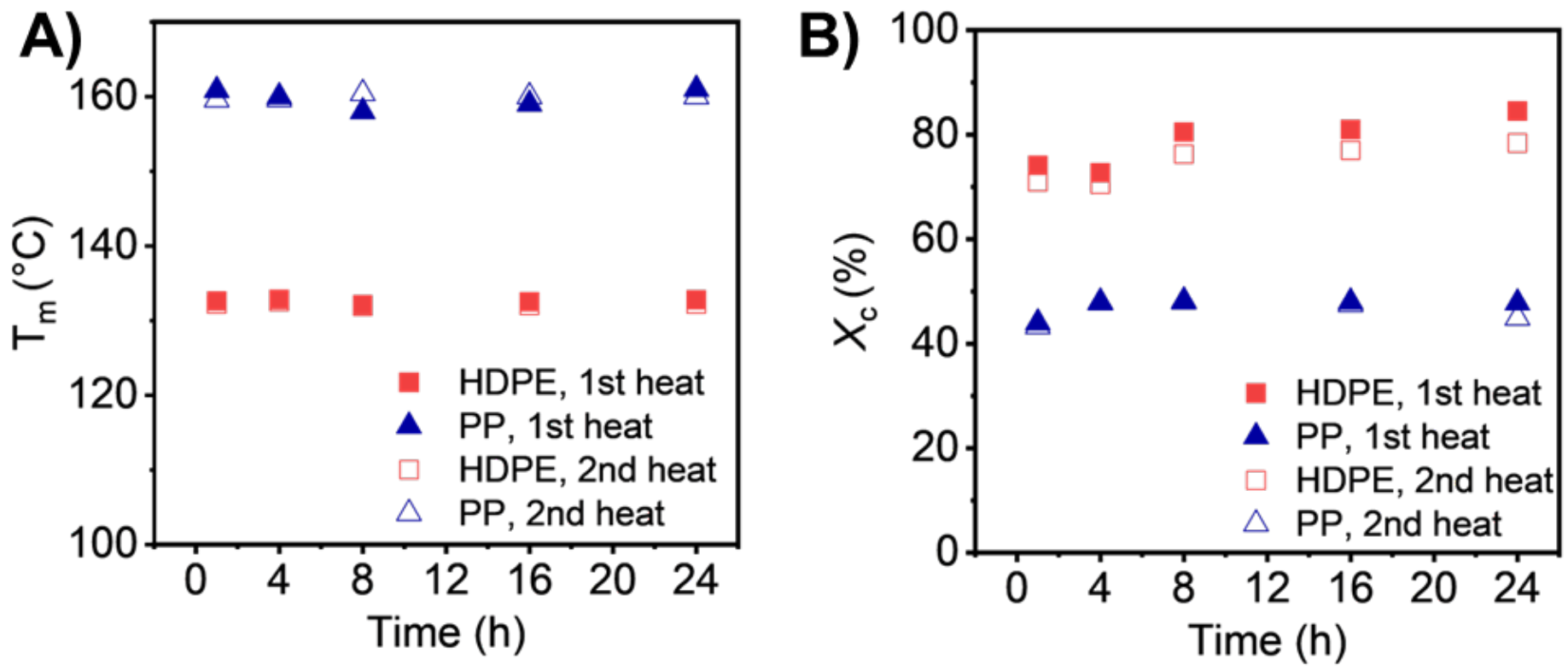

Figure S34. A) T_m and B) crystallinity of the MA functionalization of PP and HDPE (with 0.2g/mL of BPO and 1g/ mL of MA at 90 °C) as a function of time.

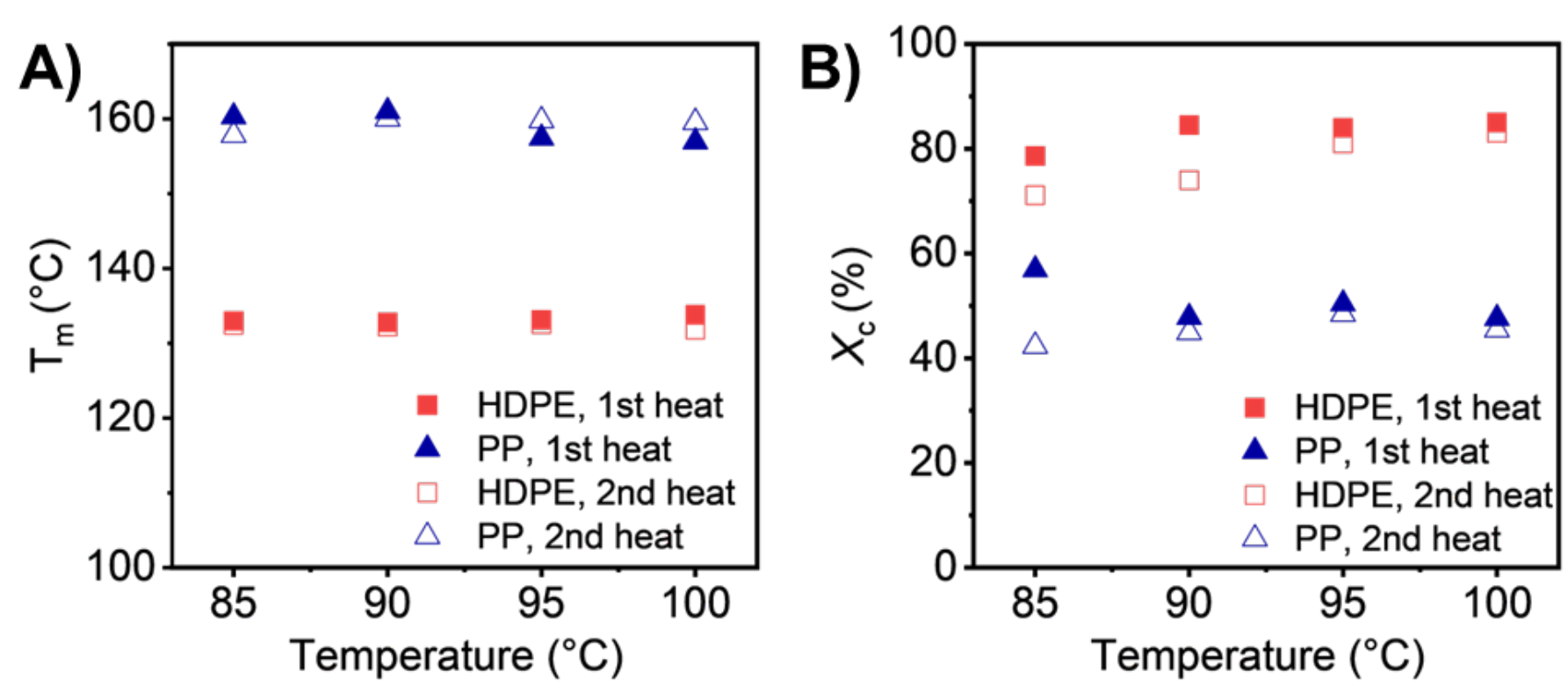

Figure S35. A) T_m and B) crystallinity of the MA functionalization of PP and HDPE (with 0.2g/mL of BPO and 1g/ mL of MA for 24h) as a function of temperature.

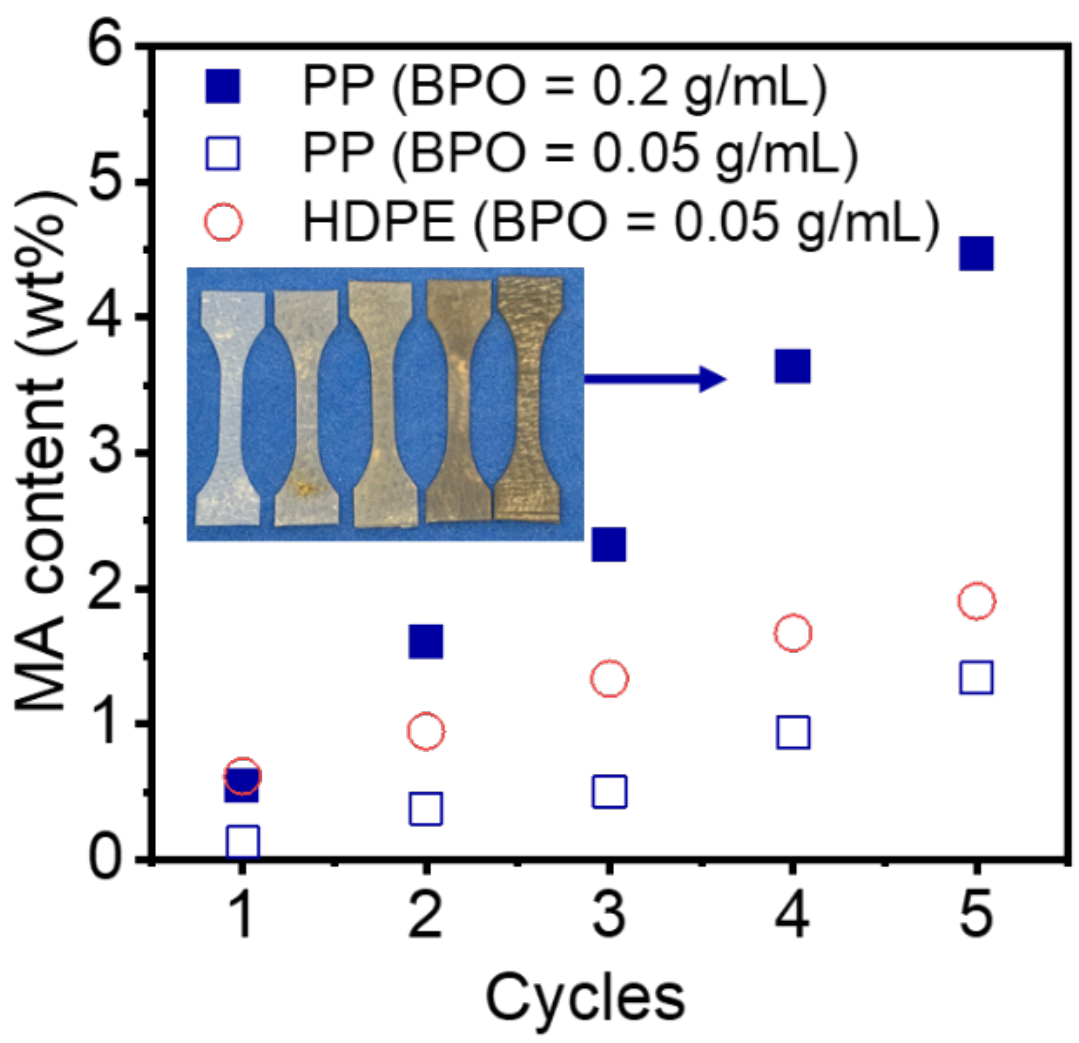

Figure S36. Incremental MA content as a function of reaction cycle numbers by sequential swelling (at 85 °C, 24 h per cycle), and a photograph of samples after each swelling cycle (left to right: 1st to the 5th cycle).

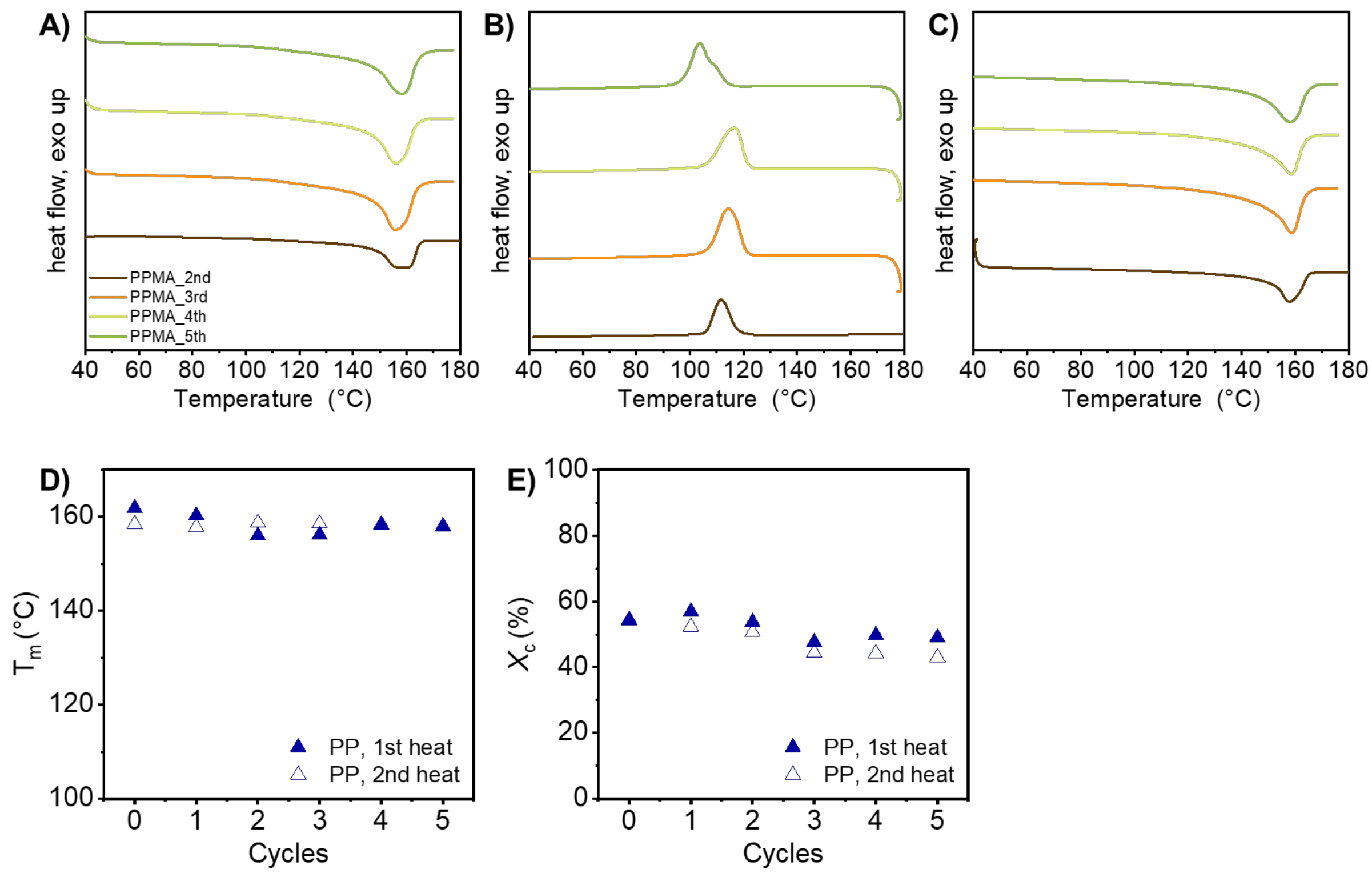

Figure S37. DSC thermograms of A) first heating, B) cooling, and C) second heating of PP-g-MA functionalized by sequential swelling with BPO=0.2g/mL, D) T_m and E) crystallinity of PP-g-MA as a function of swelling cycles.

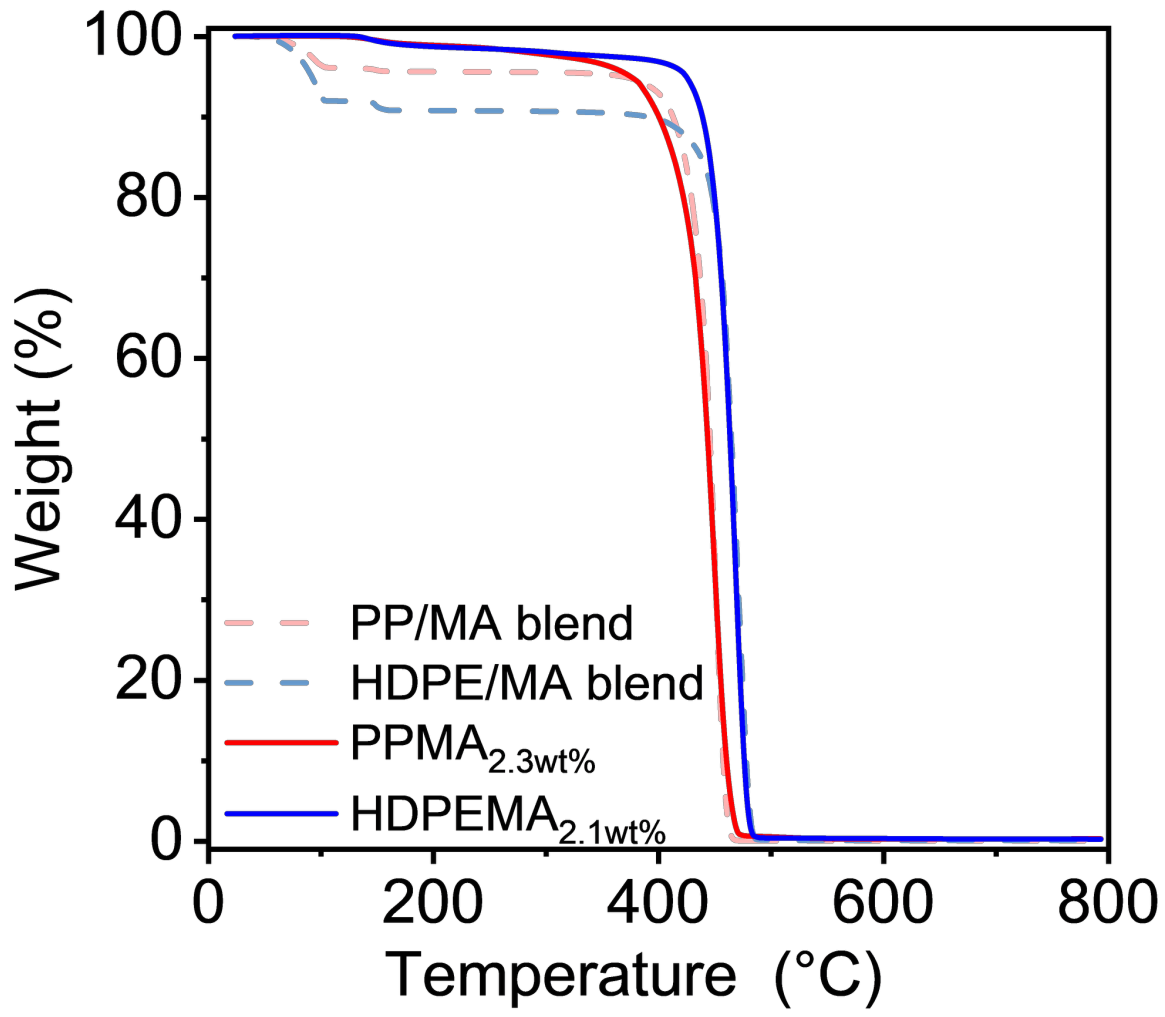

Figure S38. TGA of PP, PPMA obtained from 3 times of swelling with BPO = 0.2 g/ml (MA content = 2.3 wt%), PP/MA blend (MA ≈ 3 wt%). HDPE, HDPEMA obtained from BPO = 0.3 g/ml (MA content = 2.1 wt%), HDPE/MA blend (MA = 3 wt%)

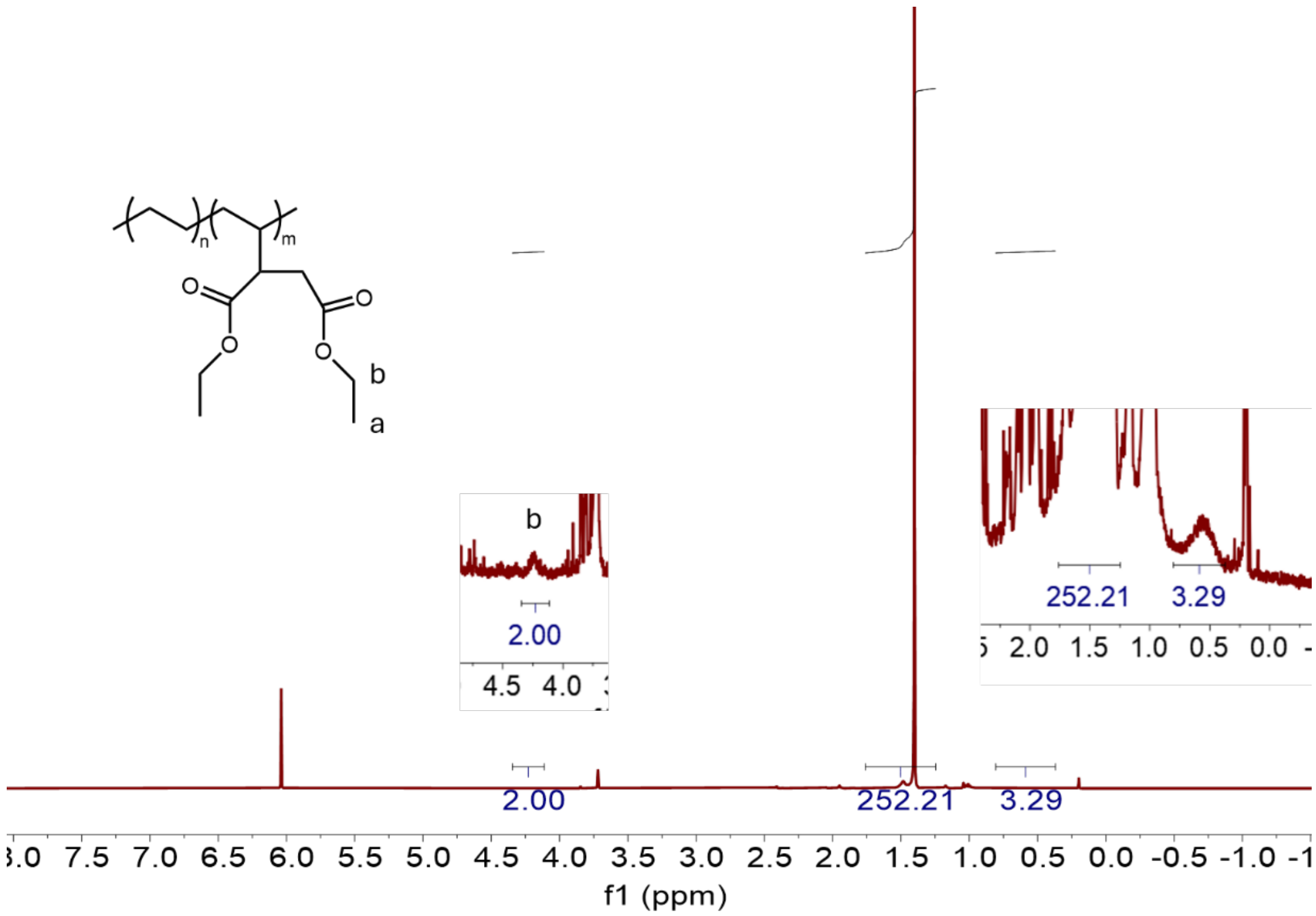

Figure S39. ¹H-NMR of diethyl maleate functionalized HDPE (HDPE-g-DM).

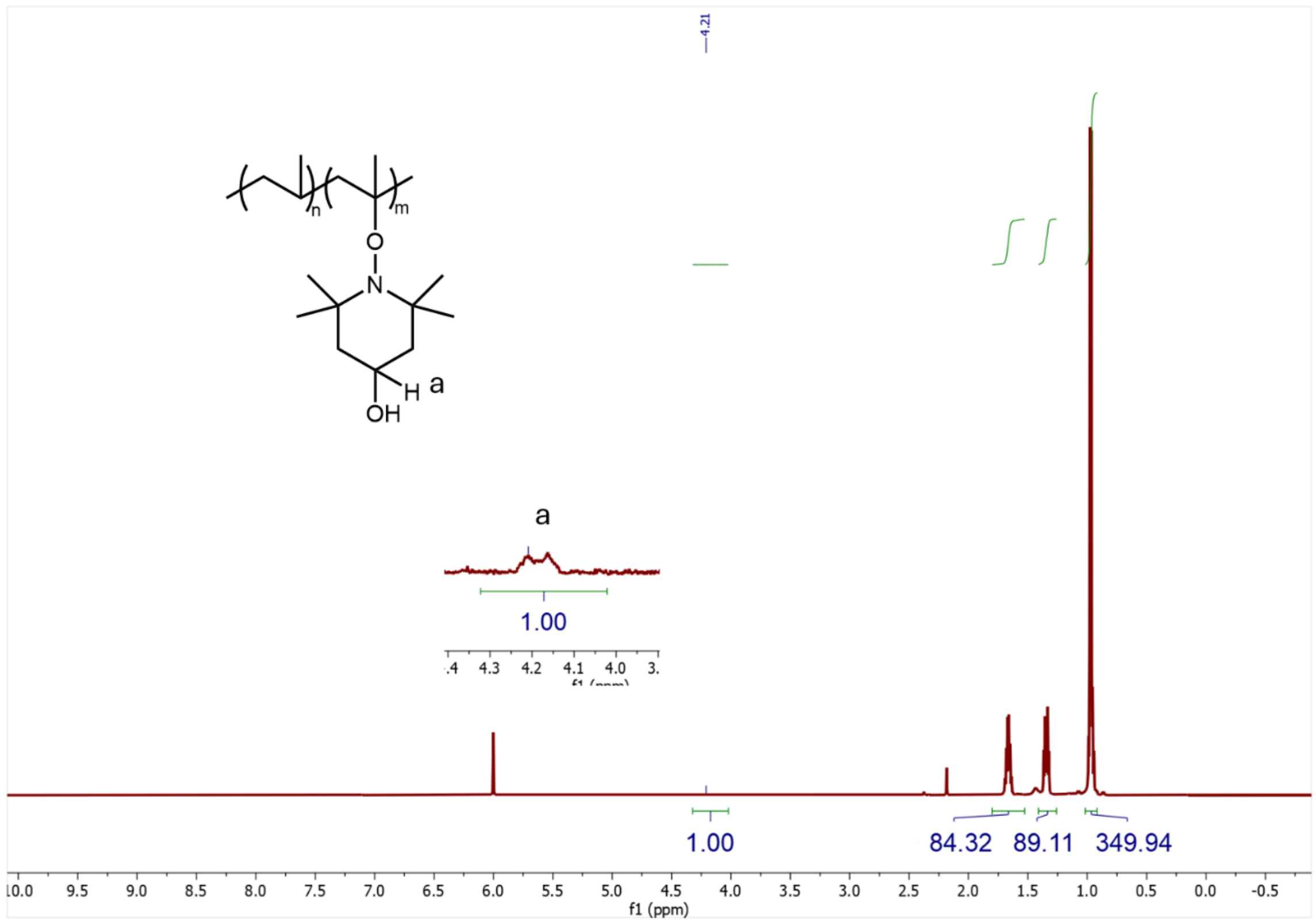

Figure S40. ^{1}H-NMR of 4-hydroxy-TEMPO functionalized PP (PP-g-TEMPO).

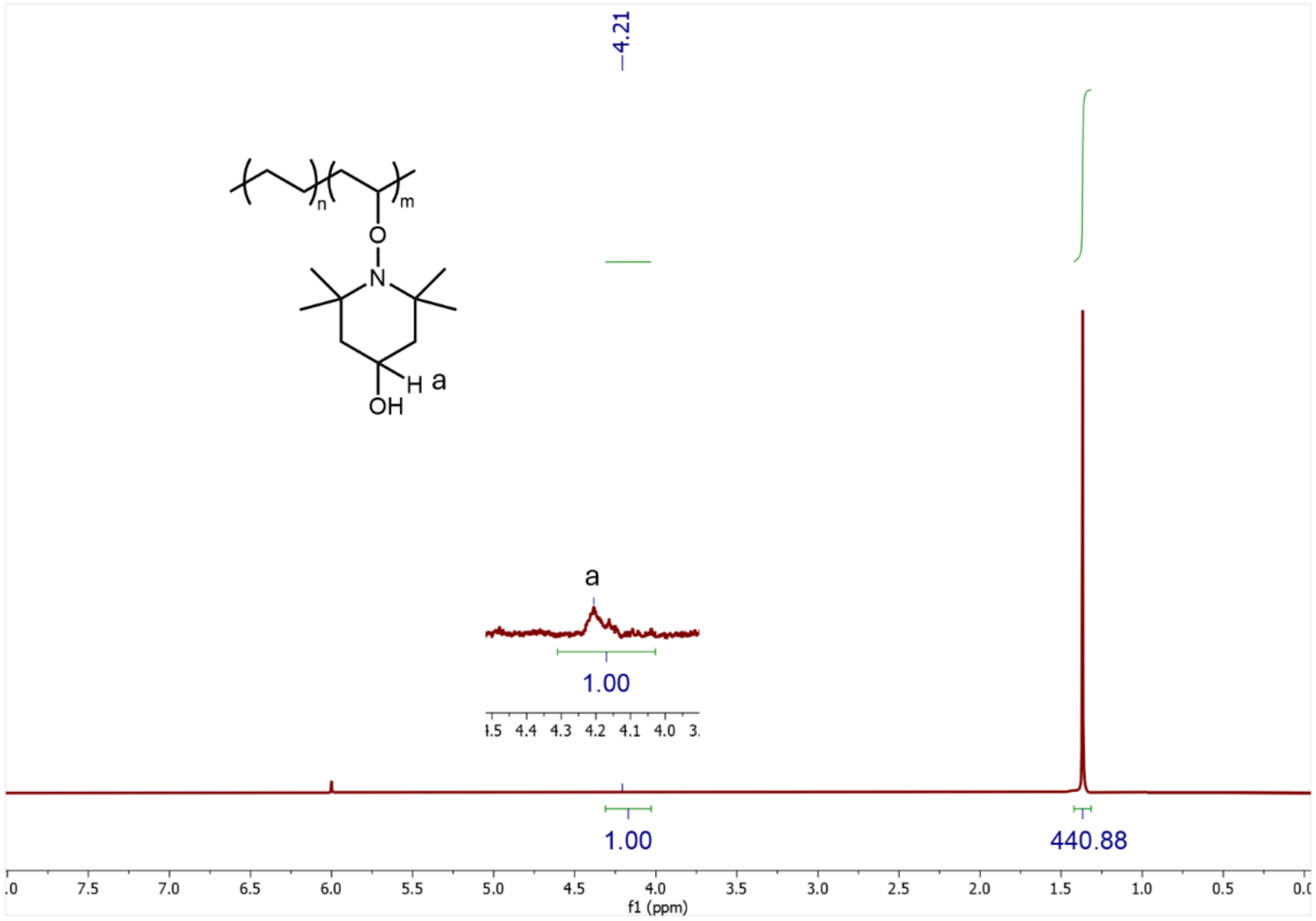

Figure S41. ^{1}H-NMR of 4-hydroxy-TEMPO functionalized HDPE (HDPE-g-TEMPO).

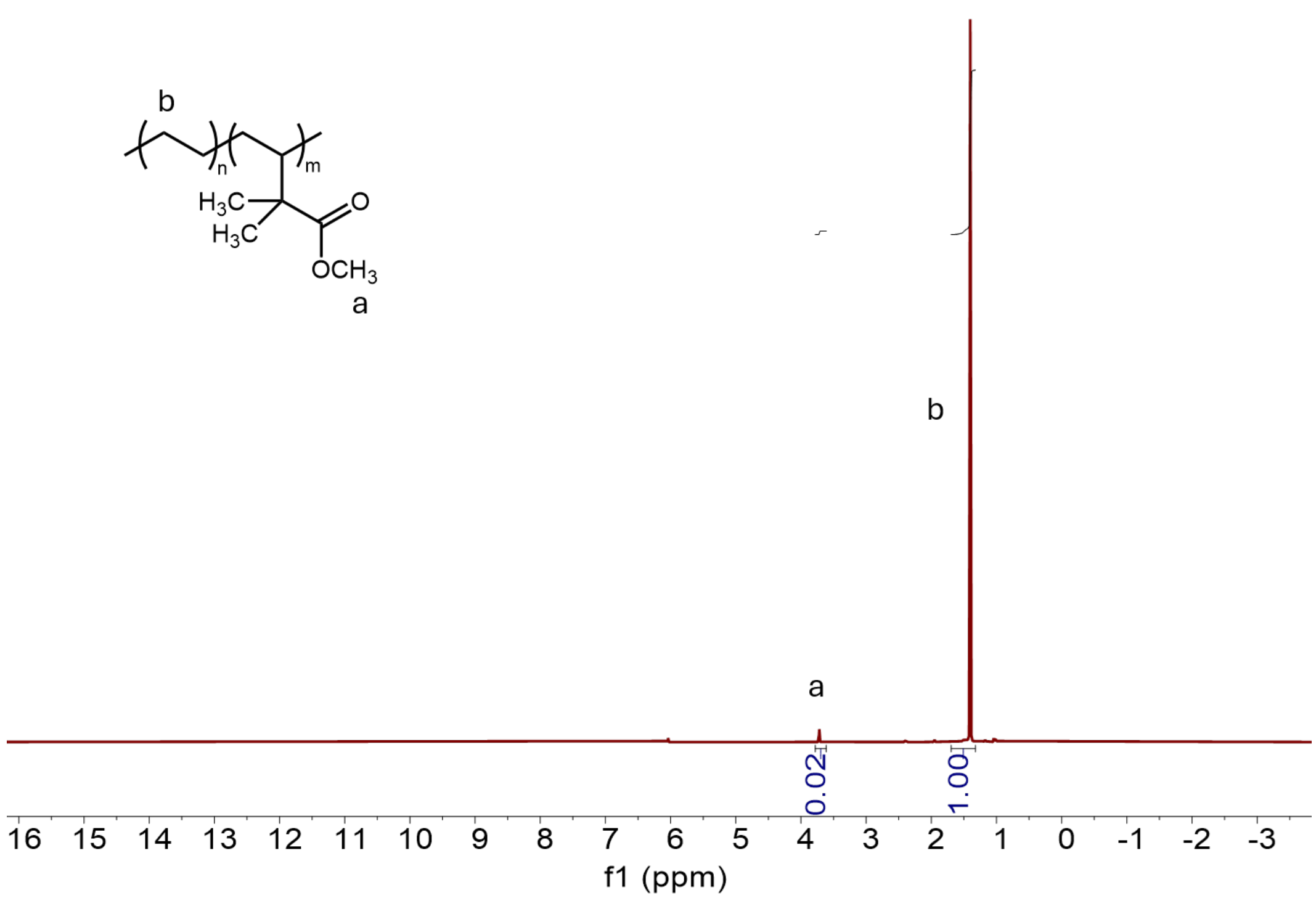

Figure S42. ^{1}H-NMR of methyl methacrylate functionalized HDPE (HDPE-g-MMA).

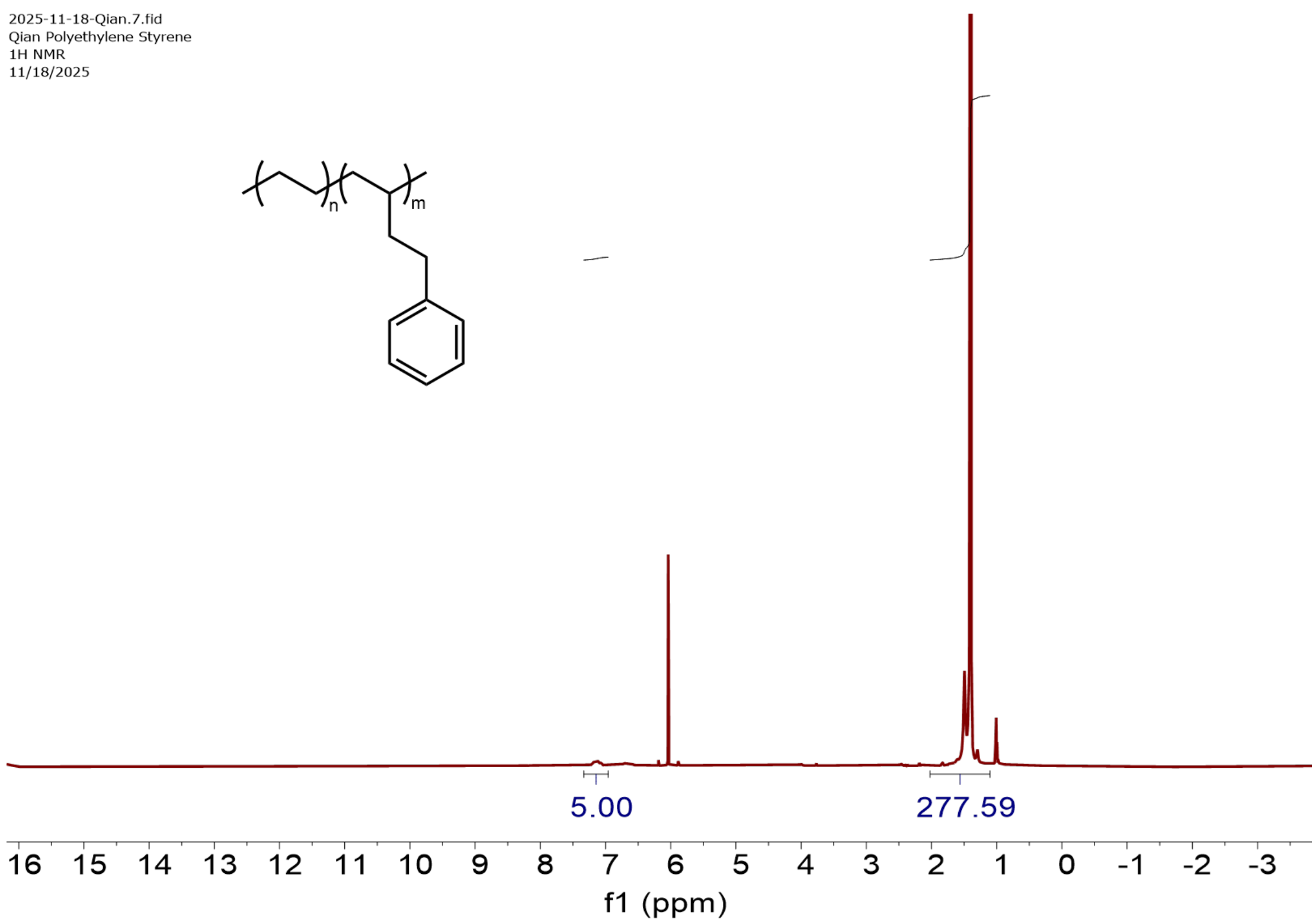

Figure S43. ^{1}H-NMR of styrene functionalized HDPE (HDPE-g-St).

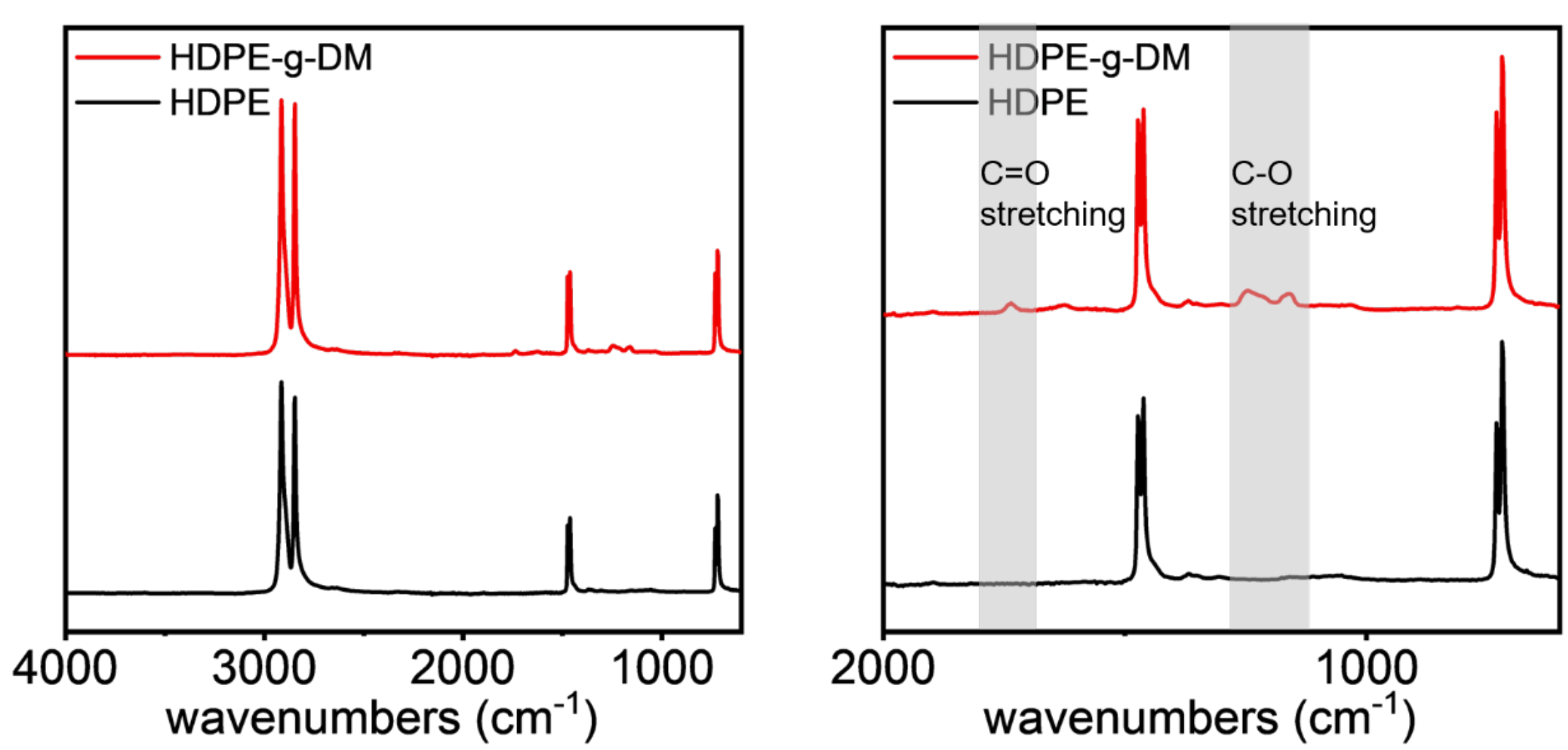

Figure S44. FT-IR of diethyl maleate functionalized HDPE (HDPE-g-DM).

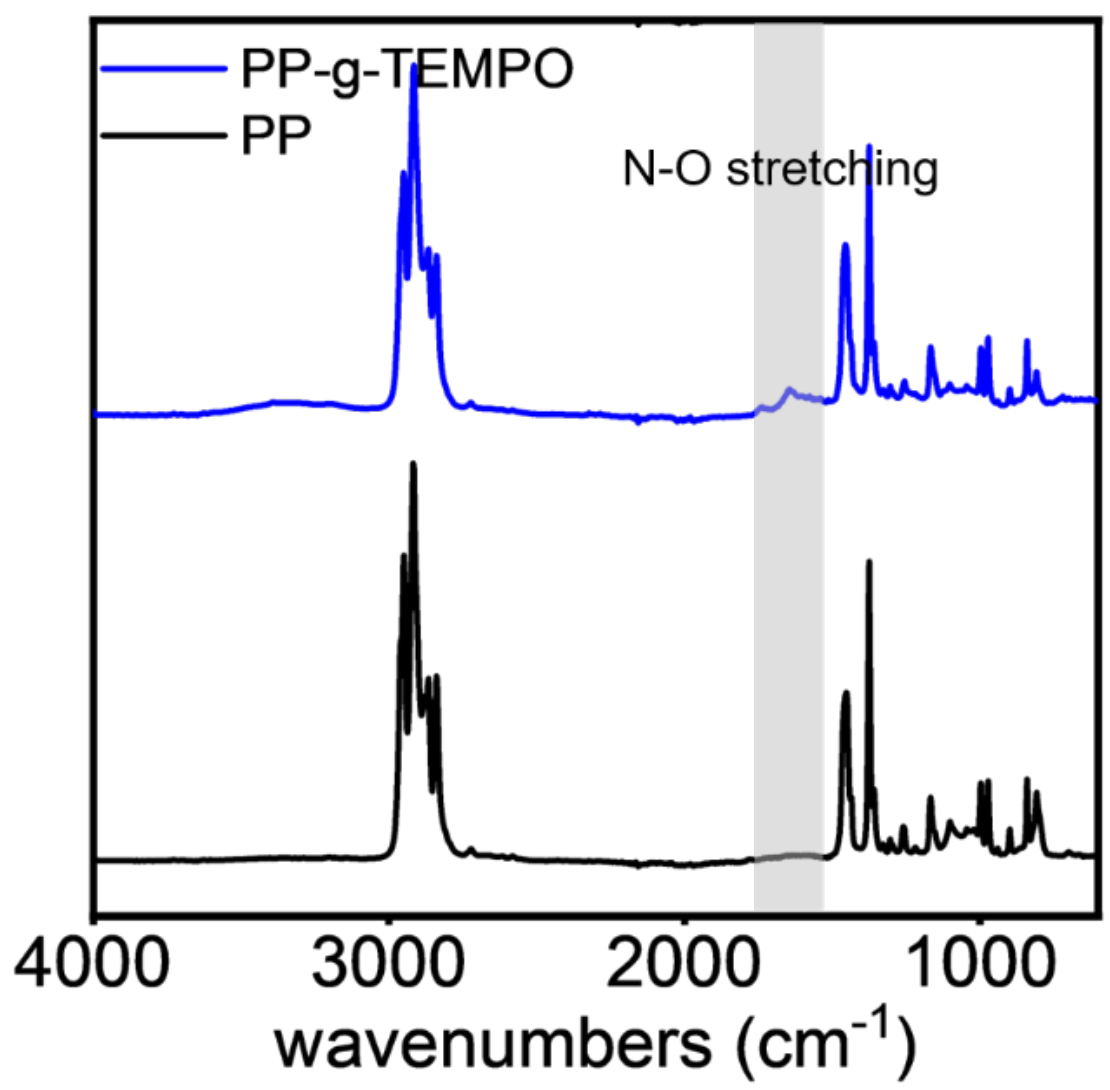

Figure S45. FT-IR of 4-hydroxy-TEMPO functionalized PP (PP-g-TEMPO).

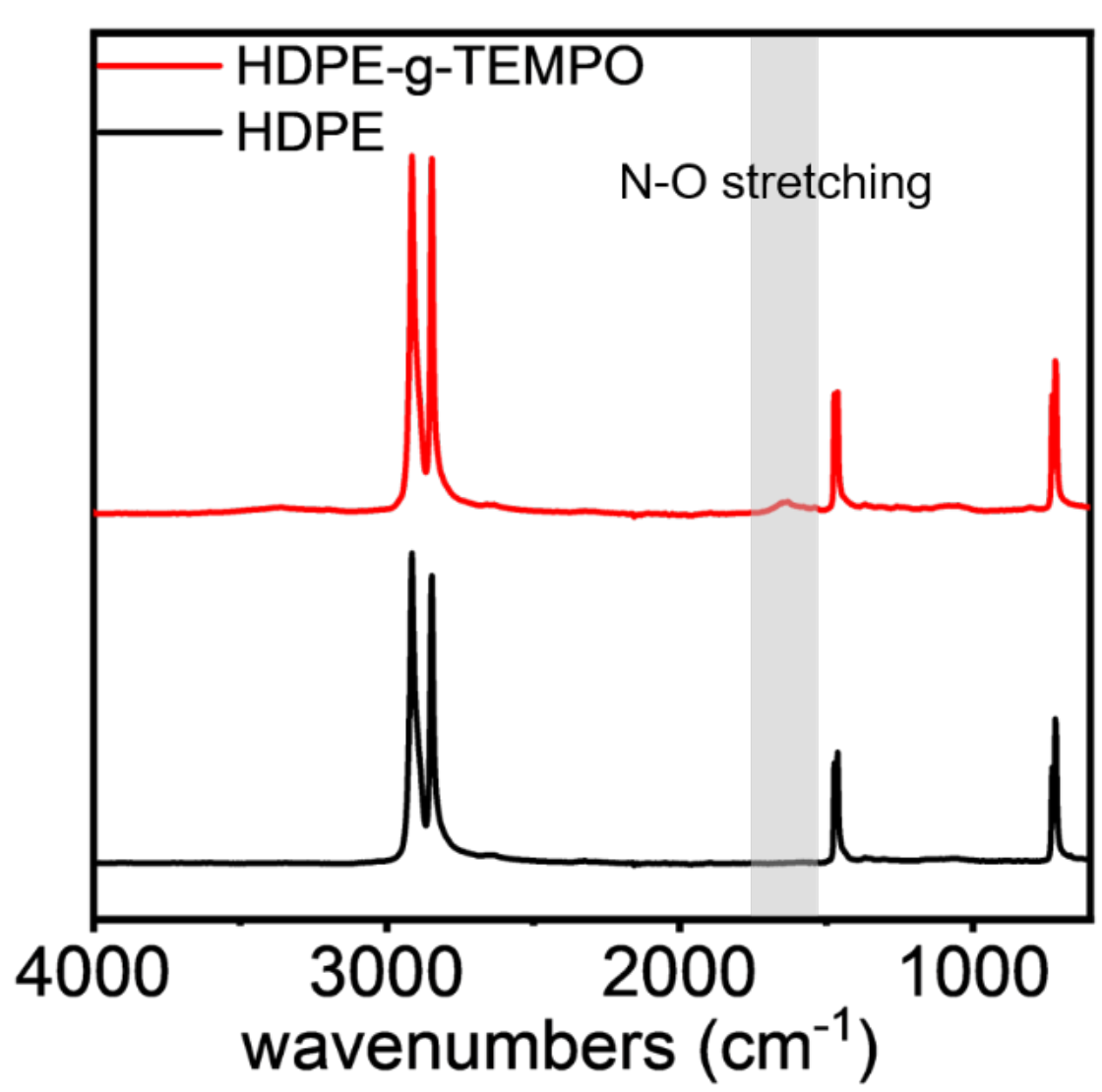

Figure S46. FT-IR of 4-hydroxy-TEMPO functionalized HDPE (HDPE-g-TEMPO).

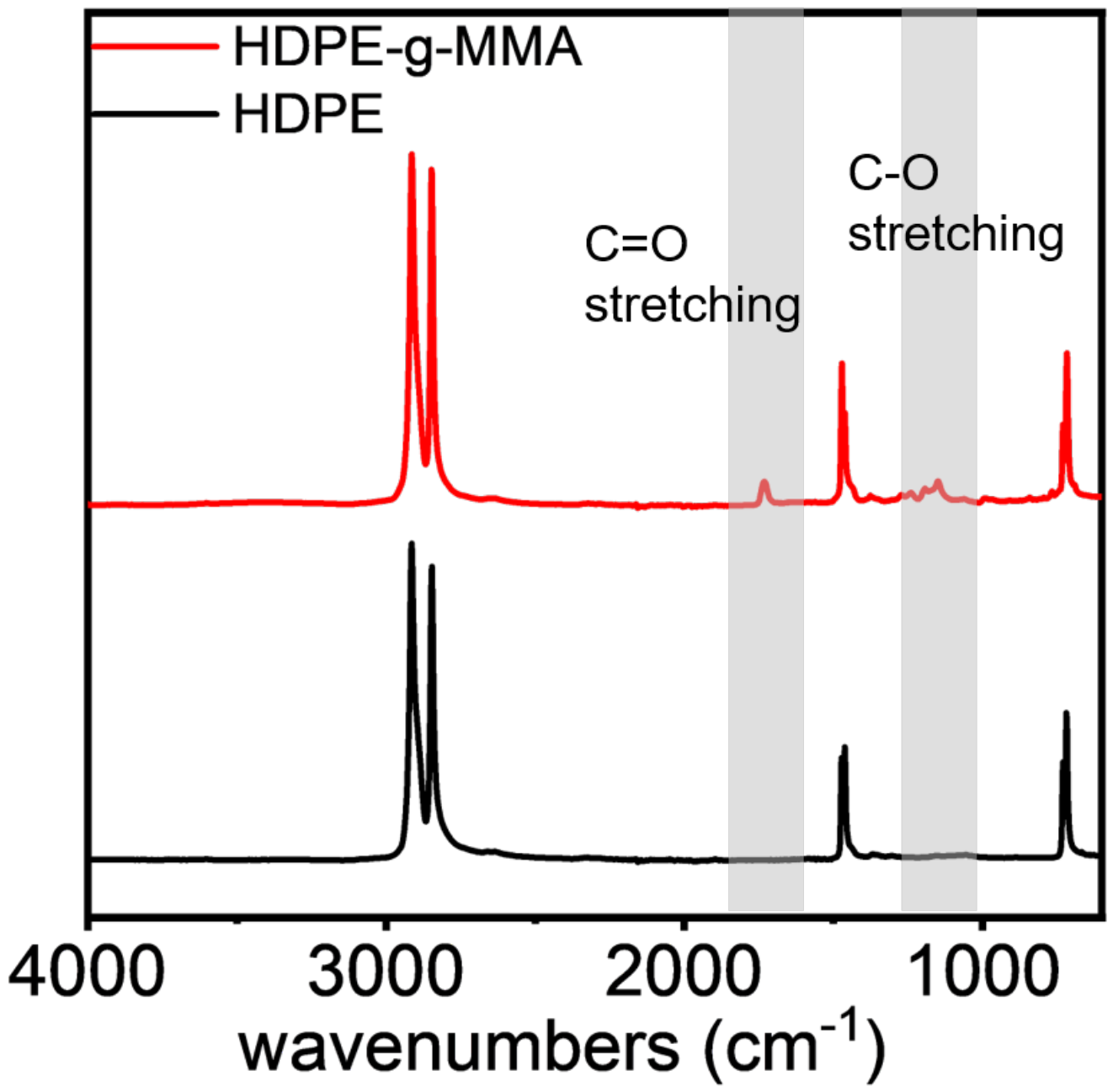

Figure S47. FT-IR of methyl methacrylate functionalized HDPE (HDPE-g-MMA).

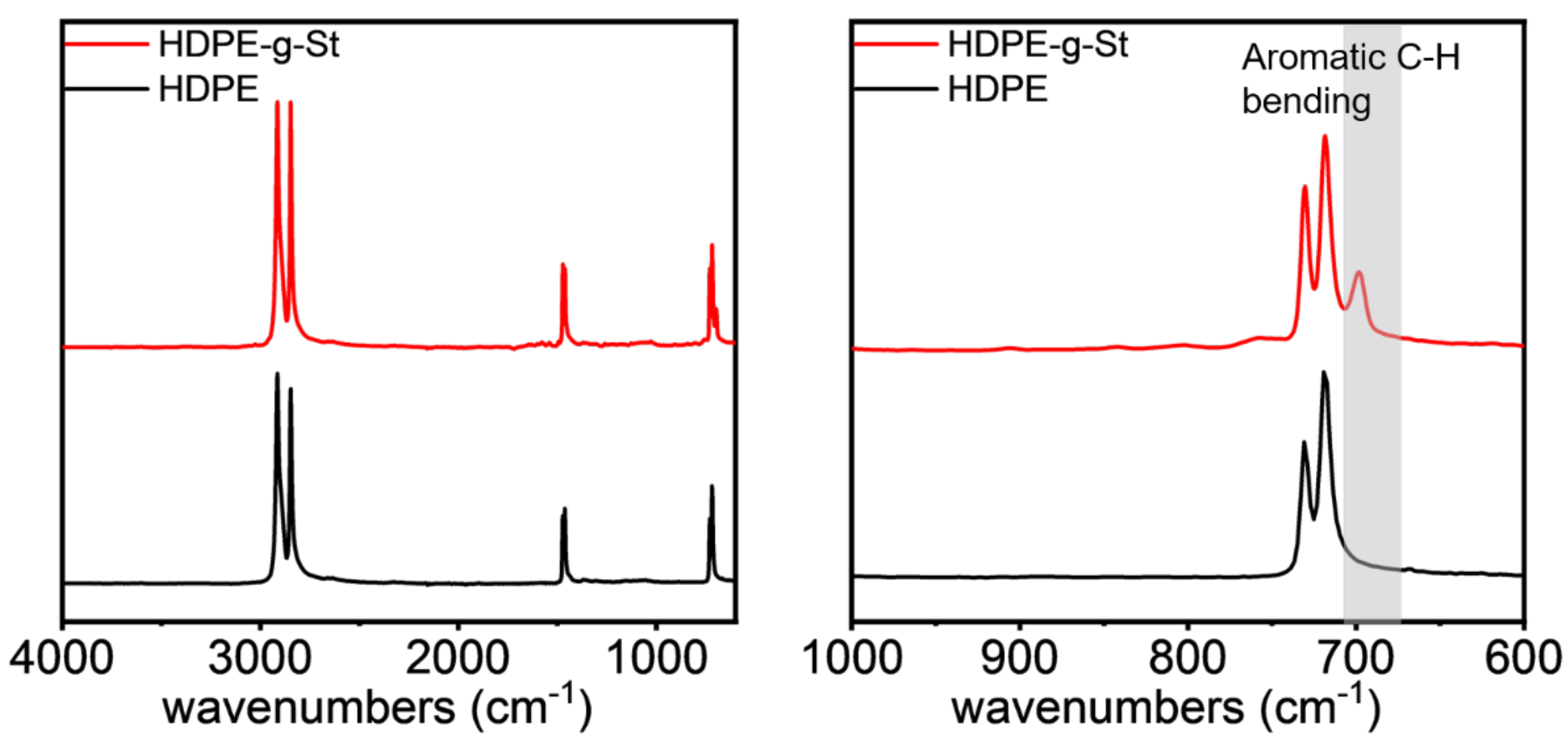

Figure S48. FT-IR of styrene functionalized HDPE (HDPE-g-St).
